# Data in support of enhancing metabolomics research through data mining

**DOI:** 10.1016/j.dib.2015.02.008

**Published:** 2015-02-27

**Authors:** Ibon Martínez-Arranz, Rebeca Mayo, Miriam Pérez-Cormenzana, Itziar Mincholé, Lorena Salazar, Cristina Alonso, José M. Mato

**Affiliations:** a OWL, Parque Tecnológico de Bizkaia, Derio, Bizkaia, Spain; b Osarten kooperatiba elkartea, Mondragón, Guipúzcoa, Spain; c CIC bioGUNE, CIBERehd, Parque Tecnológico de Bizkaia, Derio, Bizkaia, Spain

## Abstract

Metabolomics research has evolved considerably, particularly during the last decade. Over the course of this evolution, the interest in this ‘omic’ discipline is now more evident than ever. However, the future of metabolomics will depend on its capability to find biomarkers. For that reason, data mining constitutes a challenging task in metabolomics workflow. This work has been designed in support of the research article entitled “Enhancing metabolomics research through data mining”, which proposed a methodological data handling guideline. An aging research in healthy population was used as a guiding thread to illustrate this process. Here we provide a further interpretation of the obtained statistical results. We also focused on the importance of graphical visualization tools as a clue to understand the most common univariate and multivariate data analyses applied in metabolomics.

**Specifications Table**

**Table:** 

Subject area	*Chemistry/Biology*

More specific subject area	*Human metabolomics.*
Type of data	*Table, R code files, graph, figure.*
How data was acquired	*Mass spectrometry, clinical laboratory.*
Data format	*Comma-separated values (⁎.csv) tables.*
Experimental factors	*Serum samples from healthy male and female, collected under fasting conditions.*
Experimental features	Methanol and chloroform/methanol serum extracts were analyzed with three separate ultra-performance liquid chromatography-mass spectrometry based platforms.
Data source location	*Basque Country, Spain.*
Data accessibility	*Data are available here and via a web application (**http://rstudio.owlmetabolomics.com:8031/AgingAnalysis/**)*

**Value of the data**

• Metabolites related to aging in healthy population are highlighted as a result of two different post-acquisition approaches, considering age as a categorical and a continuous variable.
• R functions are provided for different statistical test, including graphical visualization tools.
• Data are presented through a web application. This is expected to help with the visualization and interpretation of univariate and multivariate data analyses.

## 1 Data

Serum samples and anthropometric data from healthy male and female volunteers included in this study were provided by the Basque Biobank for Research-OEHUN (http://www.biobancovasco.org/) and were processed with appropriate approval of the Ethics Committee. Samples were analyzed in a COBAS 6000 (Roche Diagnostics GmbH, Germany) and hematological parameters in a GEN-S (Beckman COULTER Inc., USA) at OSARTEN K.E. laboratory.

Metabolomics profiling data acquired by ultra-performance liquid chromatography coupled to mass spectrometry (UPLC-MS) were pre-processed using the TargetLynx application manager for MassLynx 4.1 (Waters Corp., Milford, MA). The peak-picking process included 466 metabolic features, identified prior to the analysis.

Then, all calculations were performed using R v.3.1.1 (R Development Core Team, 2011; http://cran.r-project.org) [1].

## 2 Experimental design, materials and methods

In metabolic profiling, there is no single platform or method to analyze the entire metabolome of a biological sample, mainly due to the wide concentration range of the metabolites coupled to their extensive chemical diversity [2,3]. The current study used multiple UPLC-MS platforms, which were optimized for extensive coverage of the serum metabolome. Metabolite extraction was accomplished by fractionating the samples into pools of species with similar physicochemical properties, using appropriate combinations of organic solvents [4]. Then, three separate UPLC-MS based platforms were used. Briefly, UPLC-single quadrupole-MS amino acid analysis system was combined with two separate UPLC-time-of-flight-MS based platforms analyzing methanol and chloroform/methanol extracts. Identified ion features in the methanol extract platform included non-esterified fatty acids, oxidized fatty acids, acyl carnitines, N-acyl ethanolamines, bile acids, steroids, monoacylglycerophospholipids, and monoetherglycerophospholipids. The chloroform/methanol extract platform provided coverage over glycerolipids, sphingolipids, diacylglycerophospholipids, acyl-ether-glycerophospholipids, cholesteryl esters, and primary fatty acid amides.

**Fig. 1:**
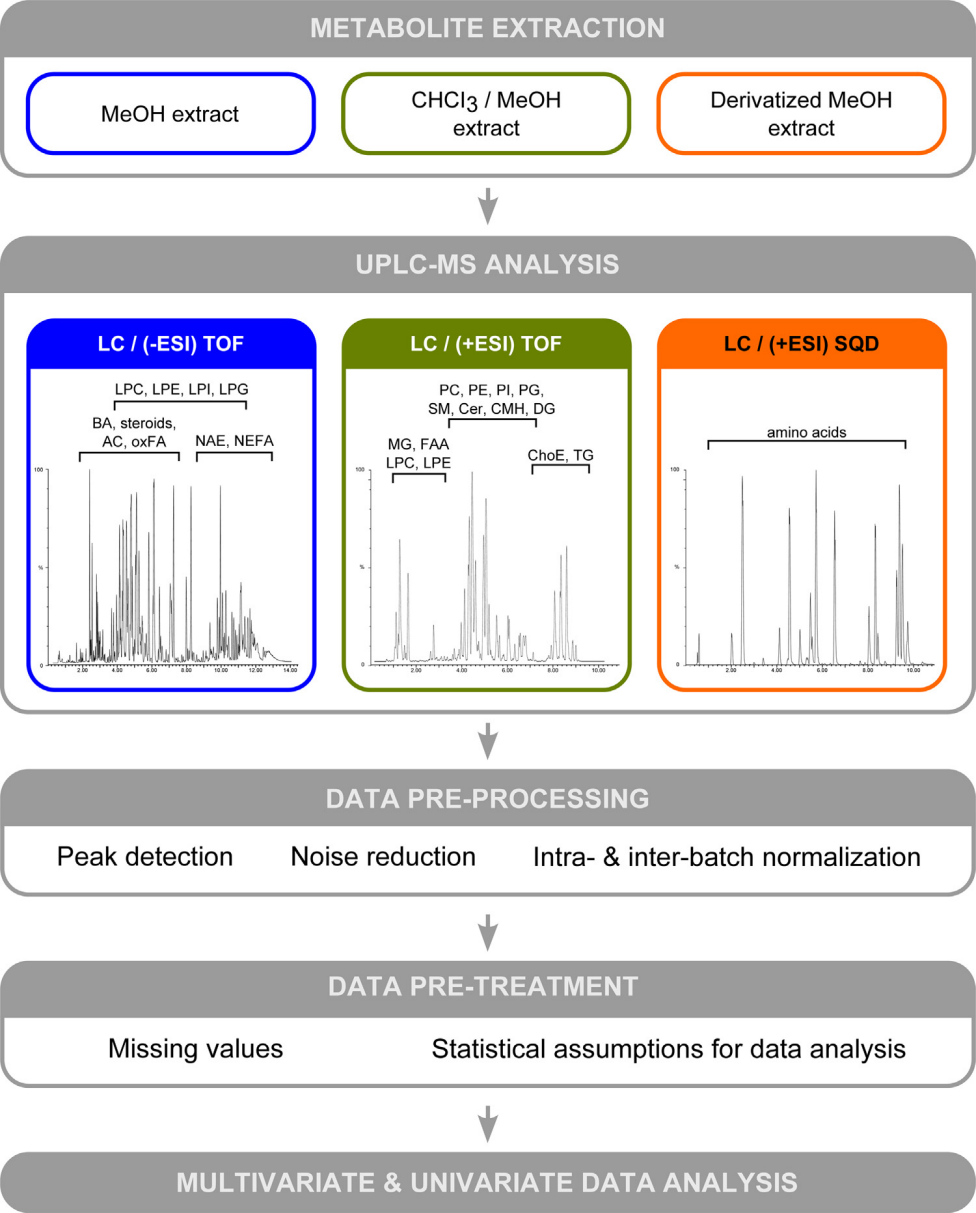
Metabolic profiling workflow applied to an aging research in healthy population. Metabolite extraction was accomplished by fractionating the samples into pools of species with similar physicochemical properties. Three separate UPLC-MS based platforms were optimized for extensive coverage of the serum metabolome. UPLC−TOF base peak ion intensity chromatograms are shown for each platform. Approximate retention time regions corresponding to identified metabolites are indicated on the chromatograms. Non-esterified fatty acids (NEFA), oxidized fatty acids (oxFA), acyl carnitines (AC), N-acyl ethanolamines (NAE), bile acids (BA), steroids, monoacylglycerophospholipids, and monoetherglycerophospholipids (LPC, LPE, LPI and LPG) are detected in the methanol extract. Additionally, mono-, di- and triglycerides (MG, DG and TG), sphingomyelins (SM) ceramides (Cer), monohexosyl ceramides (CMH), cholesteryl esters (ChoE), diacylglycerophospholipids, acyl-ether-glycerophospholipids (PC, PE, PI and PG) and primary fatty acid amides (FAA) are detected in the chloroform/methanol extract platform. Data pre-processing, data pre-treatment and data processing steps are widely described in [5].

Data pre-processing, data pre-treatment and data processing steps have been widely described [5]. A schematic flowchart of this metabolic profiling workflow is shown in Fig. 1.

## 3 Statistical analysis of anthropometric, analytical and hematological parameters

**Fig. 2:**
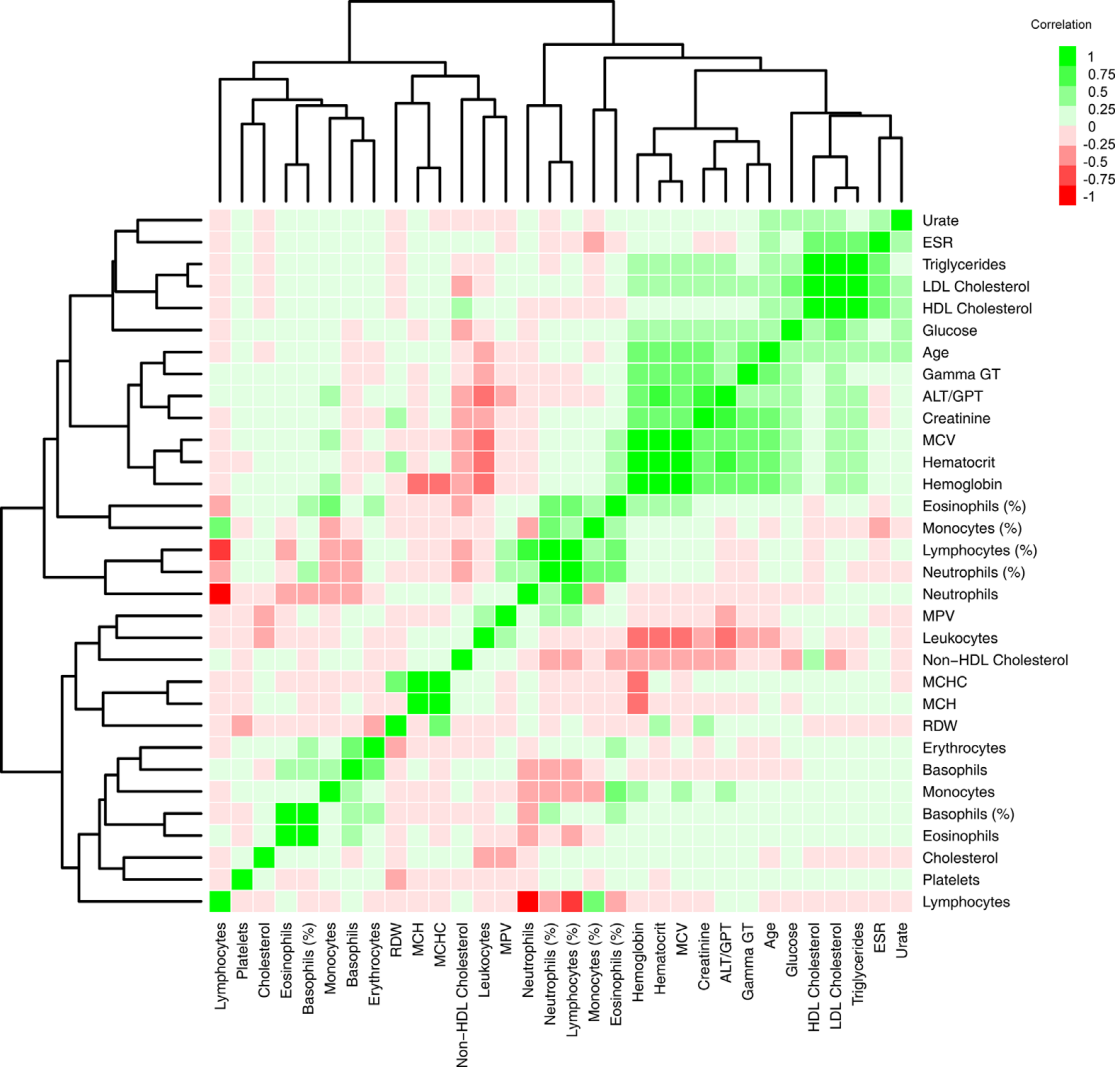
Heatmap for the correlation between age and the anthropometric, analytical and hematological parameters. Scale is based on colors from red to green representing negative and positive Spearman׳s rank correlations, respectively. Hierarchical clustering using Euclidean distance has been applied.

A heatmap for the correlation between age and the anthropometric, analytical and hematological parameters is included in Fig. 2. Variations in age and gender of each variable were evaluated by a two-way ANOVA (Table 1). The analysis per variable was completed with a boxplot and a table indicating the mean value and standard deviation per group. Those results are presented in Supplementary Material 1.

## 4 Statistical analysis and visualization

The advantages of using both univariate and multivariate approaches in data mining have been recently reviewed [6]. Both approaches are complementary and their results do not necessarily coincide. Following the advice to combine the use of both univariate and multivariate approaches, we have developed a web application. This is expected to help with the visualization and interpretation of the data analyses.

### 4.1 AgingAnalysis: an interactive web application

**Fig. 3:**
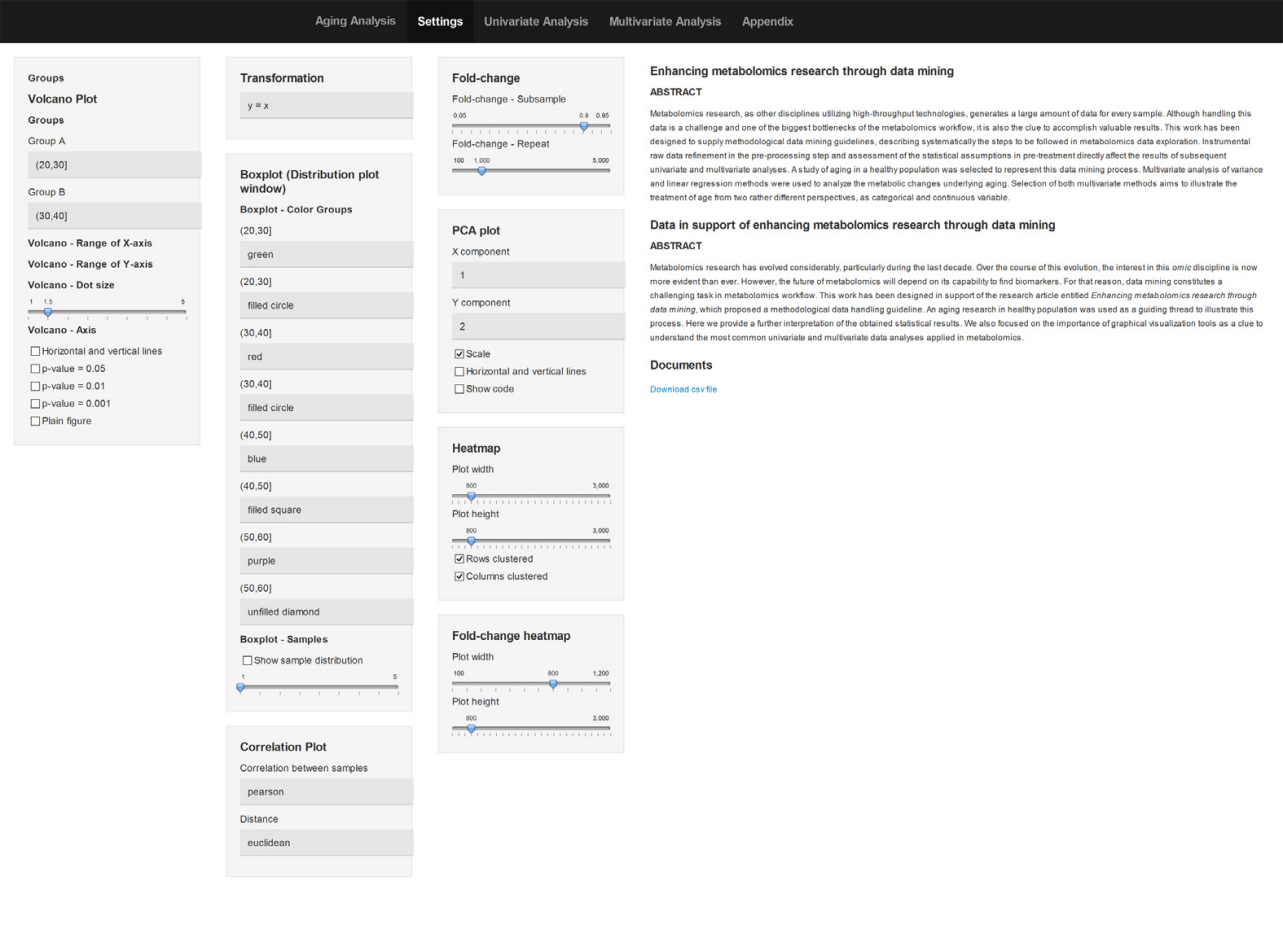
AgingAnalysis: an interactive web application for univariate and multivariate data analysis. (http://rstudio.owlmetabolomics.com:8031/AgingAnalysis/). Aging project׳s data can be downloaded from the application׳s main window.

The AgingAnalysis application has been developed using the R package shiny. This application is accessible from the following link 〈http://rstudio.owlmetabolomics.com:8031/AgingAnalysis/〉. The application itself contains a manual with the description of the different configuration options. This guide is included in the ‘Appendix’ tab. In addition, aging project׳s data can be downloaded from the web site (Fig. 3).

Univariate and multivariate analyses that can be performed through this interactive web site are briefly described.

#### 4.1.1 Univariate analysis:

Univariate data analysis indicates that only one variable is analyzed at a time. The available statistical test and visualization tools are described:

– ‘Volcano Plot’ window: Volcano plot summarizes both fold-change and *t*-test criteria. Metabolites are displayed according to the legend, unless Plain figure is selected in volcano plot settings. The following windows display the results depending on the selection of a metabolite in this plot.
– **‘**Description’ window: By clicking on a metabolite of interest on the volcano plot, this window displays its description according to The Human Metabolome Database (HMDB; http://www.hmdb.ca/)[7], Kyoto Encyclopedia of Genes and Genomes (KEGG; http://www.genome.jp/kegg/) [8] and Metabolomics Standards Initiative (MIS) [9–11].
– **‘**Boxplot’ window: This window provides the boxplot, histograms, density and Normal Q–Q plots, displaying the differences between the distributions of the aging groups for the selected variable in volcano plot. As well, homogeneity of variances test (Levene׳s test) and optimal Box-Cox transformation are presented.
– ‘Outlier Analysis’ window: A summary of the samples found to be outliers following Chauvenet´s criterion; and rebuilding of the same plots as in the ‘Boxplot’ window after removing the outlier/s.
– ‘Fold-change’ window: Histogram and fold-change of the selected metabolite, together with the criteria chosen to calculate it.
– ‘Fold-change heatmap’ window: Heatmap represents metabolomic signatures associated to aging. For each comparison, log transformed ion abundance ratios are depicted, as represented by the scale. Darker green and red colors indicate higher drops or elevations of the metabolite levels with age, respectively. Gray lines correspond to significant fold-changes of individual metabolites, darker gray colors have been used to highlight higher significances (Student׳s *t*-test *p*-value *p*<0.05, *p*<0.01 or *p*<0.001). It is relevant to highlight that metabolites present in this heatmap are ordered according to the carbon number and unsaturation degree of their esterified chains.

#### 4.1.2 Multivariate analysis:

Multivariate data approaches analyze two or more variables at once. The application provides the results of several multivariate analyses, in which the 466 metabolites are included:

– ‘PCA analysis’ window: Principal component analysis (PCA) enables easy visualization of any metabolic clustering of the different groups of samples. The scores plot displays the samples as situated on the projection planes described by the principal components; while loadings plot shows the influence of the metabolites on the clustering in the scores plot. Interpretation of the scores plot is facilitated by the loadings plot which indicates which spectral variables are responsible for the patterns and trends found. The standard deviations of the principal components are also represented; this is, the variance explained by each principal component.
– ‘Heatmap’ window: It shows the relationship among the samples and the groups according to the metabolite levels. Metabolite data are scaled, mean=0 and standard deviation=1; negative values indicate smaller amounts while positive ones indicate higher amounts of the metabolite.
– ‘Correlation plot’ window: The study of the correlation between samples according to the metabolites selected in the study. Green color indicates positive correlation, while red one denotes negative correlation. The higher the color intensity, the stronger the correlation.

### 4.2 Statistical analysis using R functions

R is a strongly functional language and an environment for statistical computing and graphical techniques [12,13]. With a freely-distributed system, R is a popular tool due to the extremely easy to learn R programming syntax, its powerful graphics facilities and the wide range of available statistical techniques.

Here, we provide R functions for three statistical tests, which include an easier determination of optimal lambda in Box-Cox transformations (tboxcox) and the determination of homoscedasticity through Levene׳s and Barlett׳s tests (levene_test and bartlett_test, respectively). These functions also include graphical visualization tools.

#### 4.2.1 Box-Cox transformations using tboxcox R function

Normal distribution of the data is one of the most important assumptions in multivariate analysis. If violated, Box-Cox transformation provides a systematic procedure for correcting this non-normal distribution. The optimal transformation is achieved by the calculation of a lambda parameter. The proposed R function tboxcox determines the optimal value for lambda, including graphical visualization tools (Supplementary Material 2). Several examples generated with this code are provided in Supplementary Material 3, illustrating the most common transformations. Those are generic examples, created by generating values of a normal distribution and applying the inverse transformations on them.

#### 4.2.2 bartlett_test and levene_test R functions for testing the homogeneity of variances

**Fig. 4:**
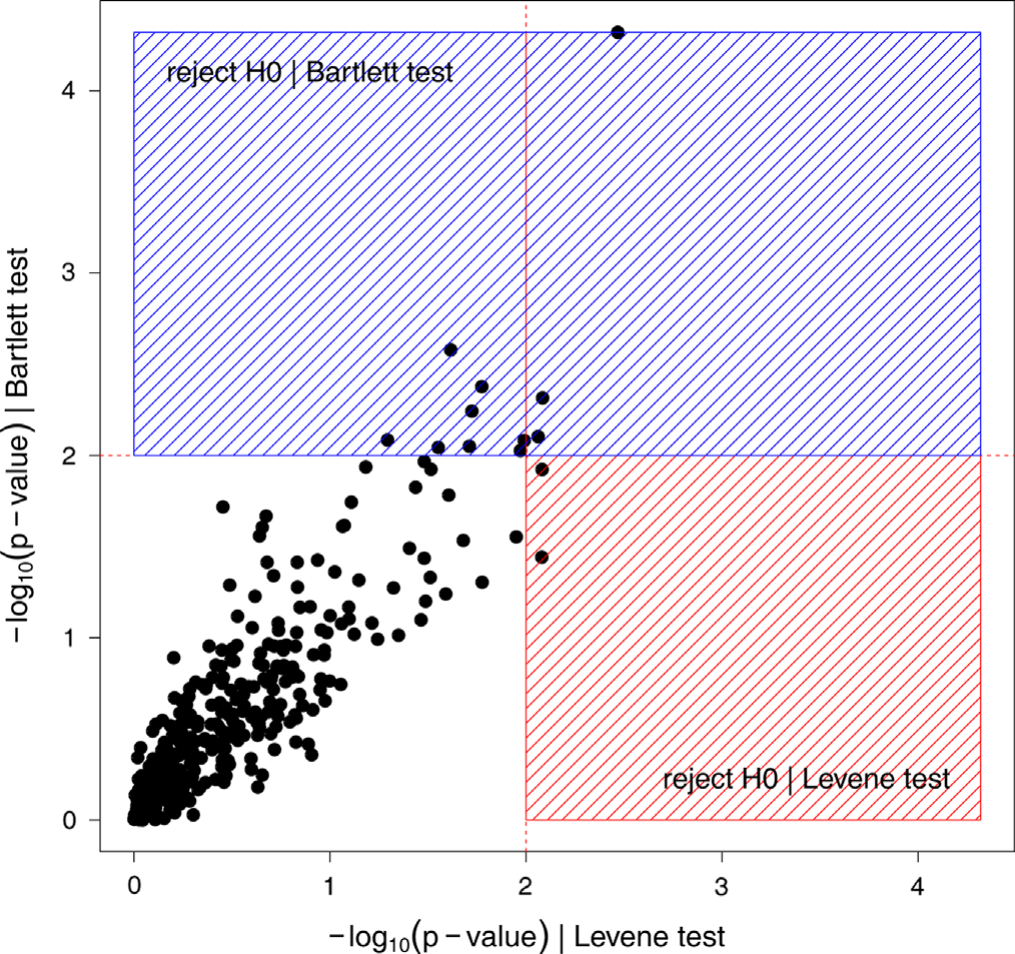
Comparison of Levene׳s and Barlett׳s tests when applied to a metabolomic profiling data.

Levene׳s and Barlett׳s tests are used to verify the homogeneity of variance. Here, R functions of both tests are provided (Supplementary Material 4). The results obtained with Levene׳s and Barlett׳s tests in our aging research data were compared. Homoscedasticity was accepted for 348 and rejected for three out of 361 variables in both cases. However, homogeneity of variance of eight and two additional variables was rejected by Barlett׳s test and Levene׳s test (*p*<0.01), respectively (Fig. 4).

In addition, the importance of the assumption of homogeneity of variance, as well as two examples of acceptance and rejection, is included in Supplementary Material 5.

## 5 MANOVA

A multivariate analysis of variance (MANOVA) was one of the multivariate models selected to decipher an aging metabolic signature [5]. This model was considered for studying age as a categorical variable, establishing the groups according to the age of the volunteers.

### 5.1 Age as independent variable

**Fig. 5:**
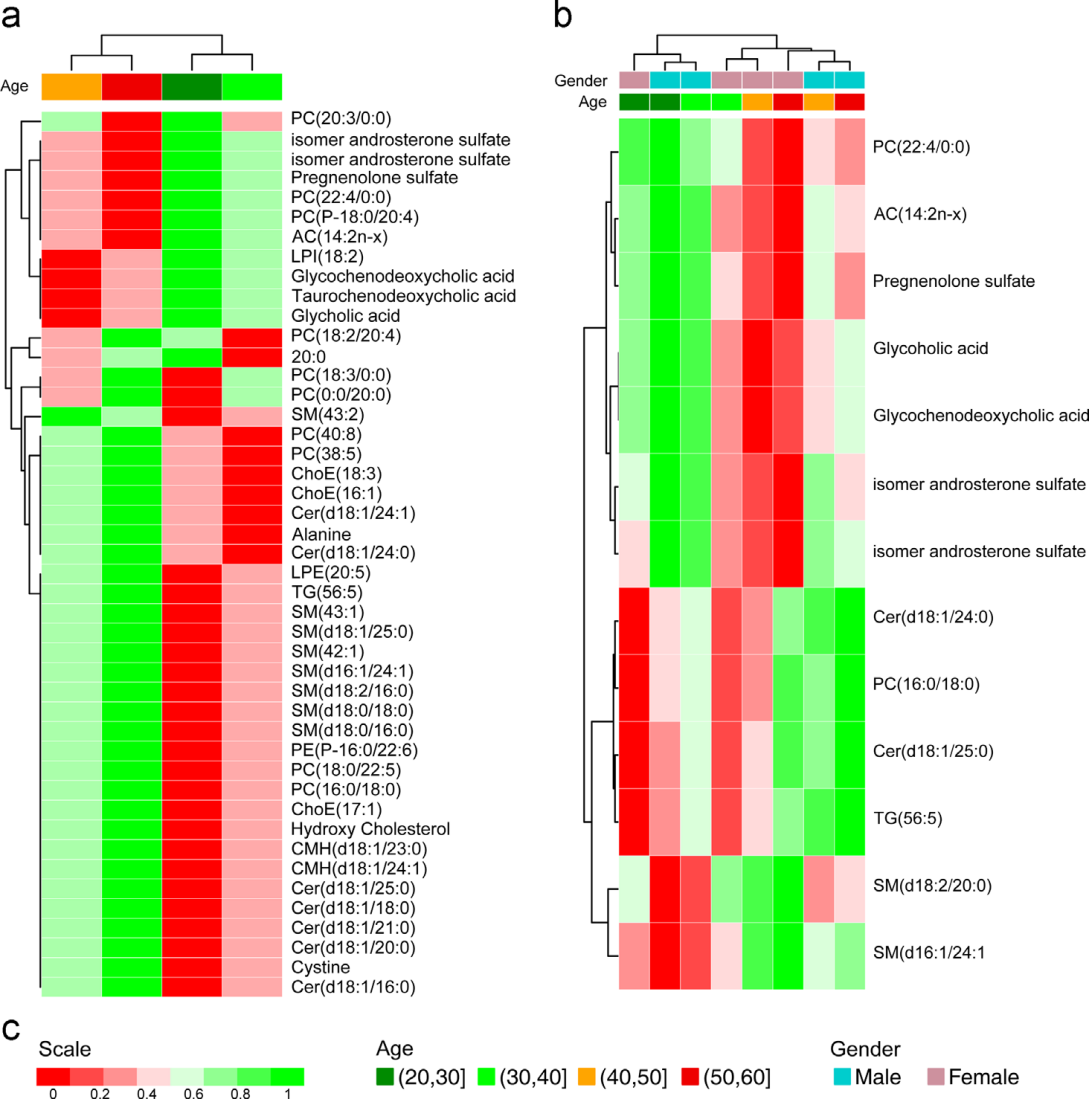
Heatmap representations of the mean vectors obtained in MAVOVA analysis. (a) Age is considered as an independent variable; (b) age and gender are considered as independent variables. Hierarchical clustering using Euclidean distance has been applied to the age groups and metabolites. Mean vectors are scaled for comparable values between metabolites. (c) Scale is based on colors from green to red, indicating higher and lower mean values respectively.

In order to fulfill the sample size requirements of the MANOVA analysis, a screening of the data was performed to find out which variables presented more evident differences among the age groups. The ANOVA test per variable revealed that 45 out of 148 metabolites agreed that *p*<0.01 (Supplementary Material Table 1). A heatmap representation of the mean vectors is depicted in Fig. 5a.

### 5.2 Age and gender as independent variables

As in the previous case, an ANOVA test was applied for each variable (Supplementary Material Table 2). Only 15 out of 141 metabolites agreed that *p*<0.01. A heatmap representation of the mean vectors is displayed in Fig. 5b.

## 6 Linear analysis

A linear least-squares regression analysis was the second multivariate model selected. In this case, age was considered as a continuous variable [5]. Previous to model construction, a random division of samples into estimation (80% of the volunteers) and validation (20%) data set was performed. Possible overfitting of the model was assessed by comparison of the residuals of both data sets. Complete information about residuals evaluation is available in Supplementary Material 6.

## 7 Session info

Information on R Session and packages versions that were used in this work:

**Table:** 

print(sessionInfo(), locale = FALSE)				
##	Platform: i386-w64-mingw32/i386 (32-bit)			
##	attached base packages:			
##	[1]	tcltk splines	grid stats	graphics grDevices utils
##	[8]	datasets	methods	base
##				
##	other attached packages:			
##	[1]	biotools_1.2	boot_1.3–11	tkrplot_0.0–23
##	[4]	rpanel_1.1–3	car_2.0–21	HH_3.1–5
##	[7]	multcomp_1.3–7	TH.data_1.0–3	survival_2.37–7
##	[10]	mvtnorm_1.0-0	latticeExtra_0.6–26 RColorBrewer_1.0–5	
##	[13]	lattice_0.20–29	royston_1.0	MVN_3.5
##	[16]	mvoutlier_2.0.5	sgeostat_1.0–25	robustbase_0.91–1
##	[19]	moments_0.13	nortest_1.0–2	mvnormtest_0.1–9
##	[22]	pls_2.4–3	MASS_7.3–33	pheatmap_0.7.7
##	[25]	xlsx_0.5.7	xlsxjars_0.6.1	rJava_0.9–6
##	[28]	knitr_1.7	googleVis_0.5.5	

## Figures and Tables

**Table 1:** Two-way ANOVA analysis of biochemical parameters. Factors: gender and age. Mean difference is significant at the 0.05 level (*p*<0.001^⁎⁎⁎^; *p*<0.01^⁎⁎^; *p*<0.05^⁎^; *p*<0.1).

**Variable**	**Age**	**Gender**	**Age:Gender**
Erythrocyte Sedimentation Rate (ESR)		^⁎⁎⁎^	
Leukocytes			
Neutrophils (%)	^⁎^	^⁎^	
Neutrophils			
Lymphocytes (%)			
Lymphocytes	^⁎⁎^		
Monocytes (%)		^⁎^	^⁎⁎^
Monocytes			
Eosinophils (%)		^⁎^	
Eosinophils		^⁎^	
Basophils (%)		^⁎^	
Basophils			
Erythrocytes	^⁎^	^⁎⁎⁎^	
Hemoglobin	^⁎⁎⁎^	^⁎⁎⁎^	
Hematocrit	^⁎⁎⁎^	^⁎⁎⁎^	
Mean Corpuscular Volumen (MCV)	^⁎^		
Mean Corpuscular Hemoglobin (MCH)			
Mean corpuscular hemoglobin concentration (MCHC)	^⁎⁎^	^⁎⁎⁎^	
Red Cell Distribution Width (RDW)			
Platelets		^⁎⁎⁎^	
Mean platelet volume (MPV)	^⁎^	^⁎^	^⁎⁎^
Cholesterol	^⁎⁎⁎^		
HDL Cholesterol		^⁎⁎⁎^	
Non-HDL Cholesterol	^⁎⁎⁎^	^⁎⁎⁎^	
LDL Cholesterol	^⁎⁎⁎^	^⁎⁎⁎^	
Triglycerides		^⁎⁎⁎^	
Glucose	^⁎⁎⁎^	^⁎^	
Urate		^⁎⁎⁎^	
Creatinine		^⁎⁎⁎^	^⁎⁎^
ALT/GPT	^⁎^	^⁎⁎⁎^	
Gamma GT (GGT)	^⁎⁎⁎^	^⁎⁎⁎^	^⁎⁎^
