# Supplementary material for: Data in support of enhancing metabolomics research through data mining
Source: Data Brief. 2015 Feb 27;3:155–64. doi: 10.1016/j.dib.2015.02.008 (PMC4510074; doi:10.1016/j.dib.2015.02.008)
Supplement: Supplementary file 1 — Supplementary Material [file mmc1.doc]

## Supplemetary Material 1

#### Analysis of variable Body Mass Index (BMI)

**Table**: Mean value and standard deviation of Body Mass Index per age and gender.

|  | Male | Female |
| --- | --- | --- |
| (20,30] | 23.6$\pm$ (2.56) | 23.2$\pm$ (2.68) |
| (30,40] | 24.4$\pm$ (2.38) | 22.1$\pm$ (2.55) |
| (40,50] | 25.1$\pm$ (2.61) | 23.0$\pm$ (2.02) |
| (50,60] | 24.9$\pm$ (2.36) | 24.0$\pm$ (2.8) |


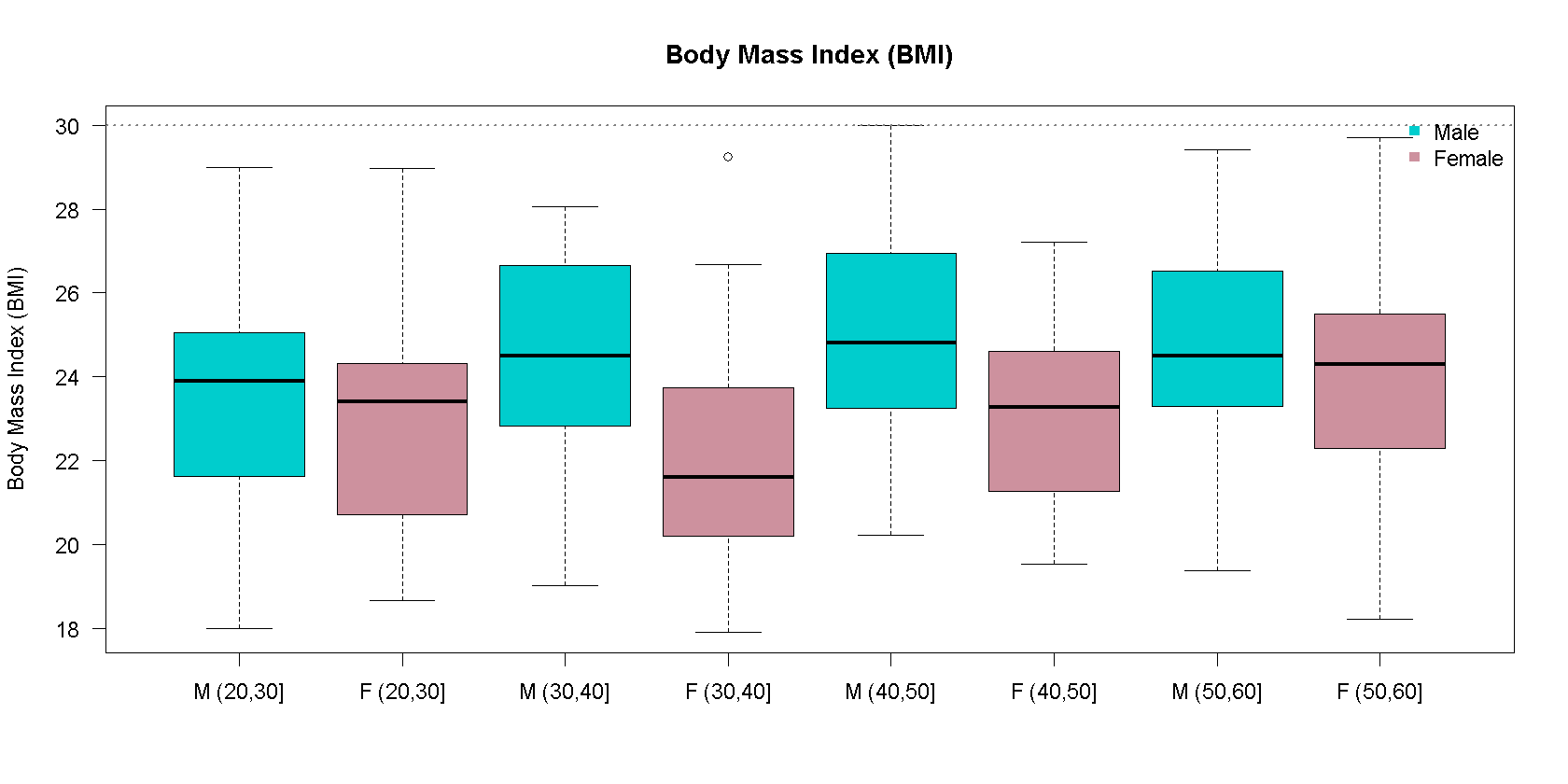


**Figure**: Boxplot displaying the distribution of Body Mass Index of subjects by age and gender.

## Collected clinical data

For each biochemical variable a boxplot a table indicating the mean value and standard deviation and a two-way ANOVA per age and gender are presented.

#### Analysis of variable Erythrocyte Sedimentation Rate (ESR)


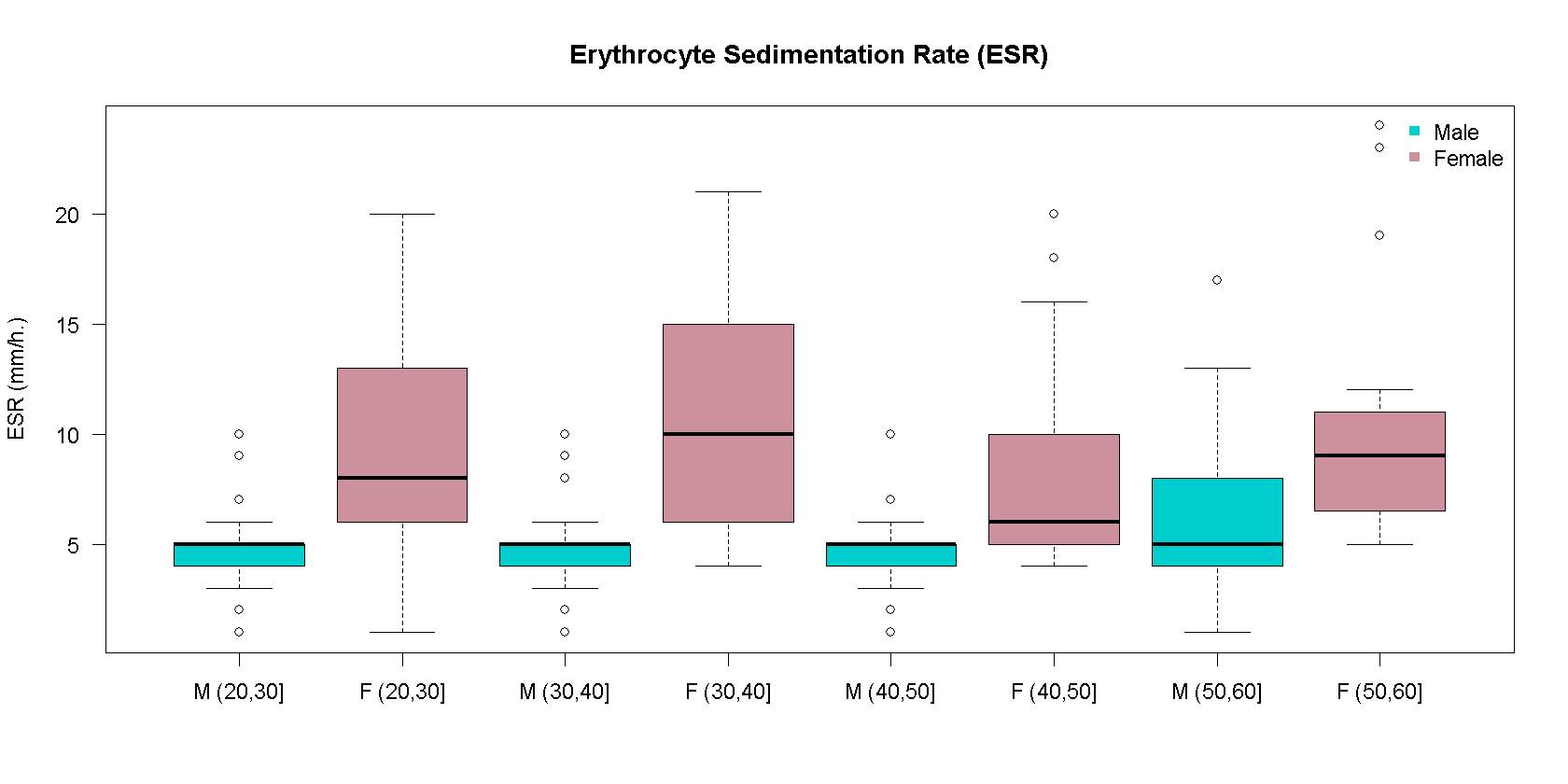


**Figure.** Erythrocyte Sedimentation Rate (ESR). Boxplot

**Table.** Mean value and standard deviation of Erythrocyte Sedimentation Rate (ESR) variable. ESR (<15 mm/h.)

|  | Male | Female |
| --- | --- | --- |
| (20,30] | 4.65$\pm$ (1.87) | 9.83$\pm$ (6.05) |
| (30,40] | 4.58$\pm$ (1.93) | 10.6$\pm$ (5.1) |
| (40,50] | 4.72$\pm$ (1.67) | 8.88$\pm$ (5.26) |
| (50,60] | 5.94$\pm$ (3.46) | 10.3$\pm$ (5.14) |

**Table.** Two-way ANOVA analysis for Erythrocyte Sedimentation Rate (ESR) variable by gender and age. ESR (<15 mm/h.)

|  | Df | Sum Sq | Mean Sq | F value | Pr(>F) |
| --- | --- | --- | --- | --- | --- |
| Age | 3 | 80.14222 | 26.71407 | 1.7317145 | 0.1609318 |
| Gender | 1 | 1547.26737 | 1547.26737 | 100.3001397 | 0.0000000 |
| Age:Gender | 3 | 40.97977 | 13.65992 | 0.8854917 | 0.4491535 |
| Residuals | 255 | 3933.72512 | 15.42637 |  |  |

#### Analysis of variable Leukocytes


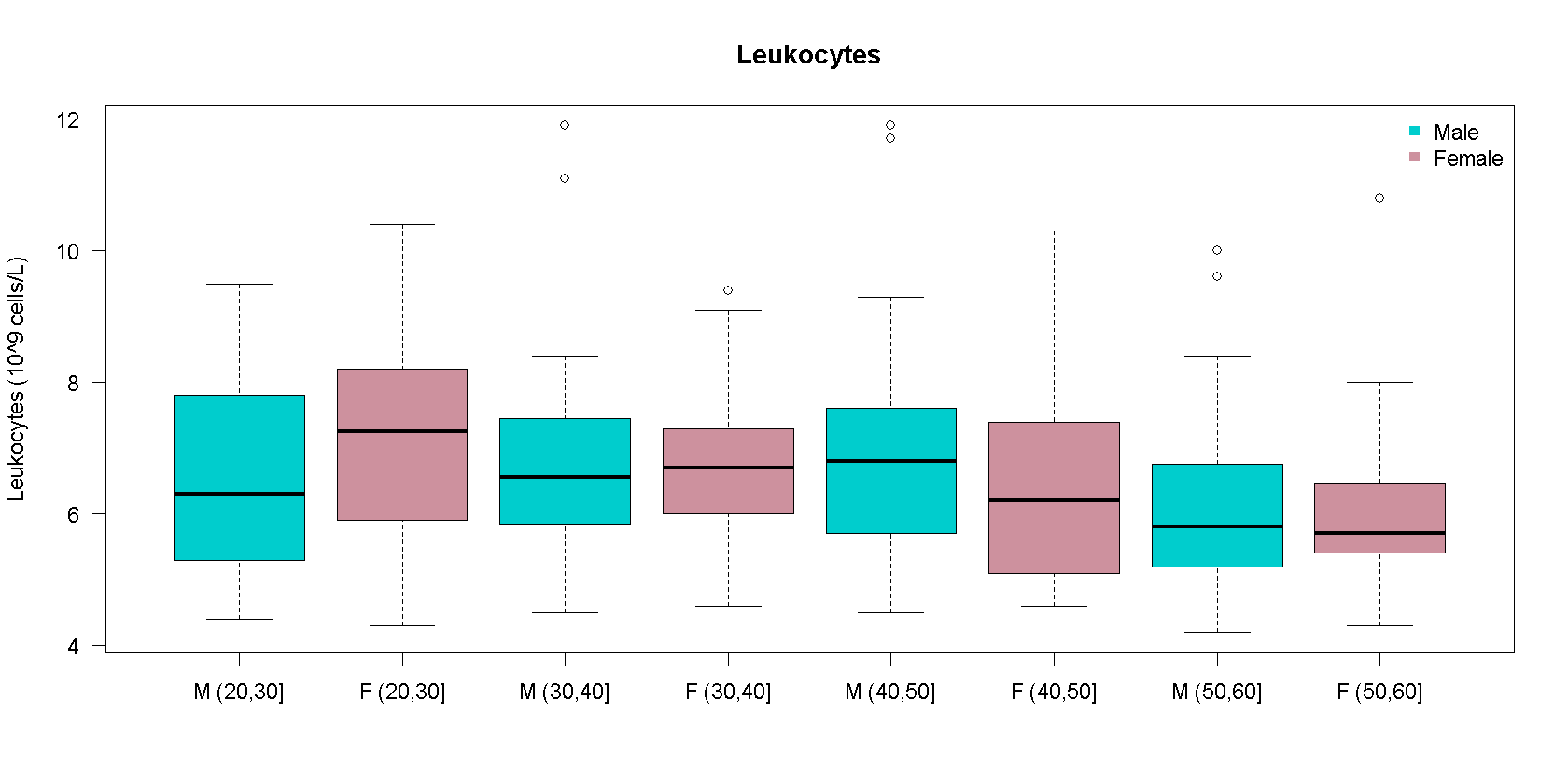


**Figure.** Leukocytes. Boxplot

**Table.** Mean value and standard deviation of Leukocytes variable. Leukocytes (4-11 10^9 cells/L)

|  | Male | Female |
| --- | --- | --- |
| (20,30] | 6.62$\pm$ (1.42) | 7.08$\pm$ (1.68) |
| (30,40] | 6.75$\pm$ (1.56) | 6.74$\pm$ (1.12) |
| (40,50] | 6.93$\pm$ (1.66) | 6.4$\pm$ (1.41) |
| (50,60] | 6.2$\pm$ (1.39) | 6.11$\pm$ (1.39) |

**Table.** Two-way ANOVA analysis for Leukocytes variable by gender and age. Leukocytes (4-11 10^9 cells/L)

|  | Df | Sum Sq | Mean Sq | F value | Pr(>F) |
| --- | --- | --- | --- | --- | --- |
| Age | 3 | 14.4616533 | 4.8205511 | 2.2880796 | 0.0790160 |
| Gender | 1 | 0.5718996 | 0.5718996 | 0.2714527 | 0.6028111 |
| Age:Gender | 3 | 7.3633468 | 2.4544489 | 1.1650068 | 0.3236171 |
| Residuals | 255 | 537.2367791 | 2.1068109 |  |  |

#### Analysis of variable Neutrophils (%)


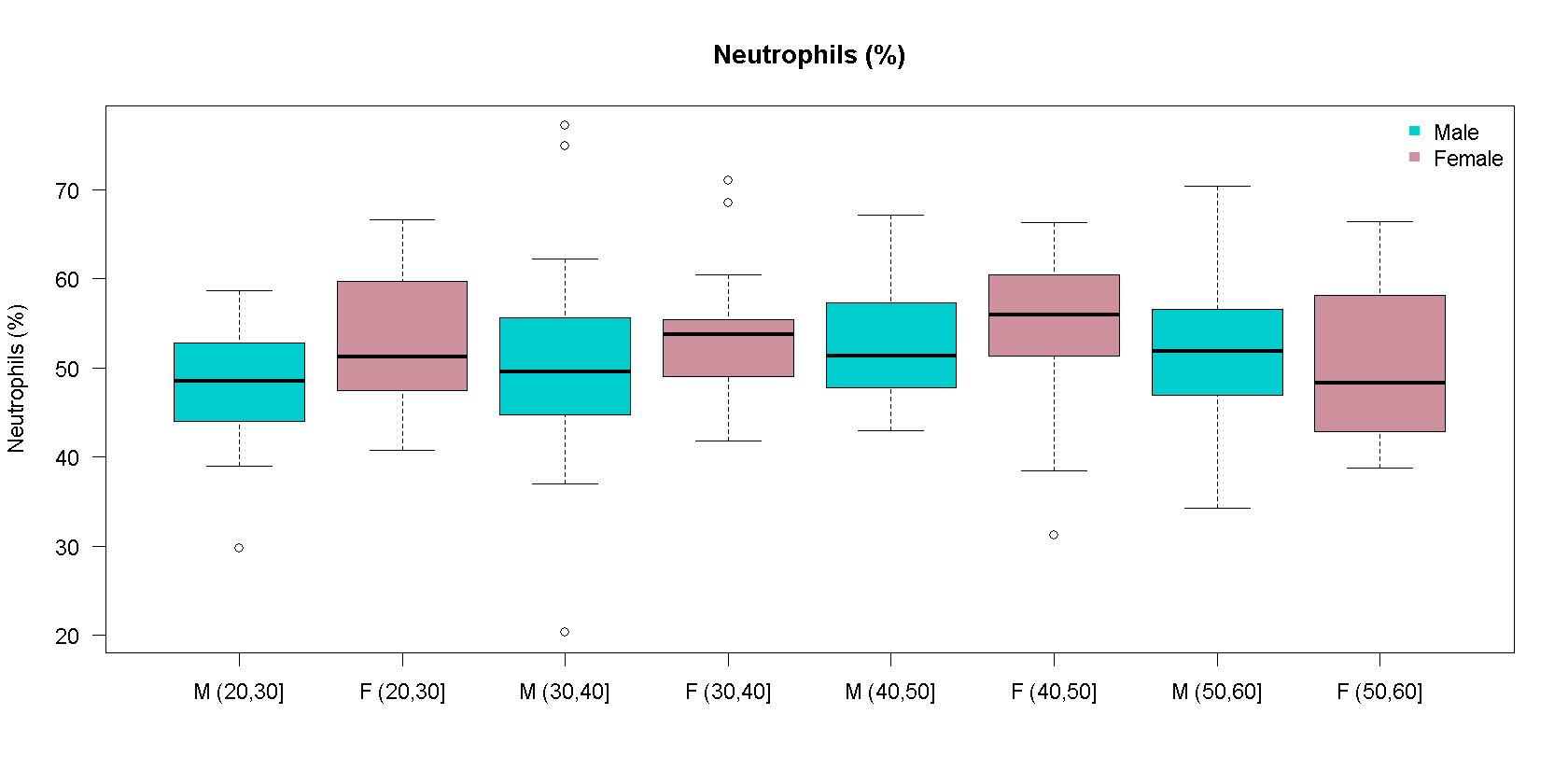


**Figure. Neutrophils (%)**. Boxplot

**Table.** Mean value and standard deviation of Neutrophils (%) variable. Neutrophils % (37-70%)

|  | Male | Female |
| --- | --- | --- |
| (20,30] | 48$\pm$ (6.29) | 53.2$\pm$ (8.24) |
| (30,40] | 50.2$\pm$ (10.1) | 53.1$\pm$ (6.46) |
| (40,50] | 52.5$\pm$ (6.33) | 54.3$\pm$ (8.06) |
| (50,60] | 51.6$\pm$ (7.26) | 50.1$\pm$ (8.18) |

**Table.** Two-way ANOVA analysis for Neutrophils (%) variable by gender and age. Neutrophils % (37-70%)

|  | Df | Sum Sq | Mean Sq | F value | Pr(>F) |
| --- | --- | --- | --- | --- | --- |
| Age | 3 | 470.8678 | 156.95593 | 2.696010 | 0.0464694 |
| Gender | 1 | 268.8704 | 268.87039 | 4.618349 | 0.0325732 |
| Age:Gender | 3 | 301.7996 | 100.59988 | 1.727990 | 0.1616890 |
| Residuals | 255 | 14845.5538 | 58.21786 |  |  |

#### Analysis of variable Neutrophils


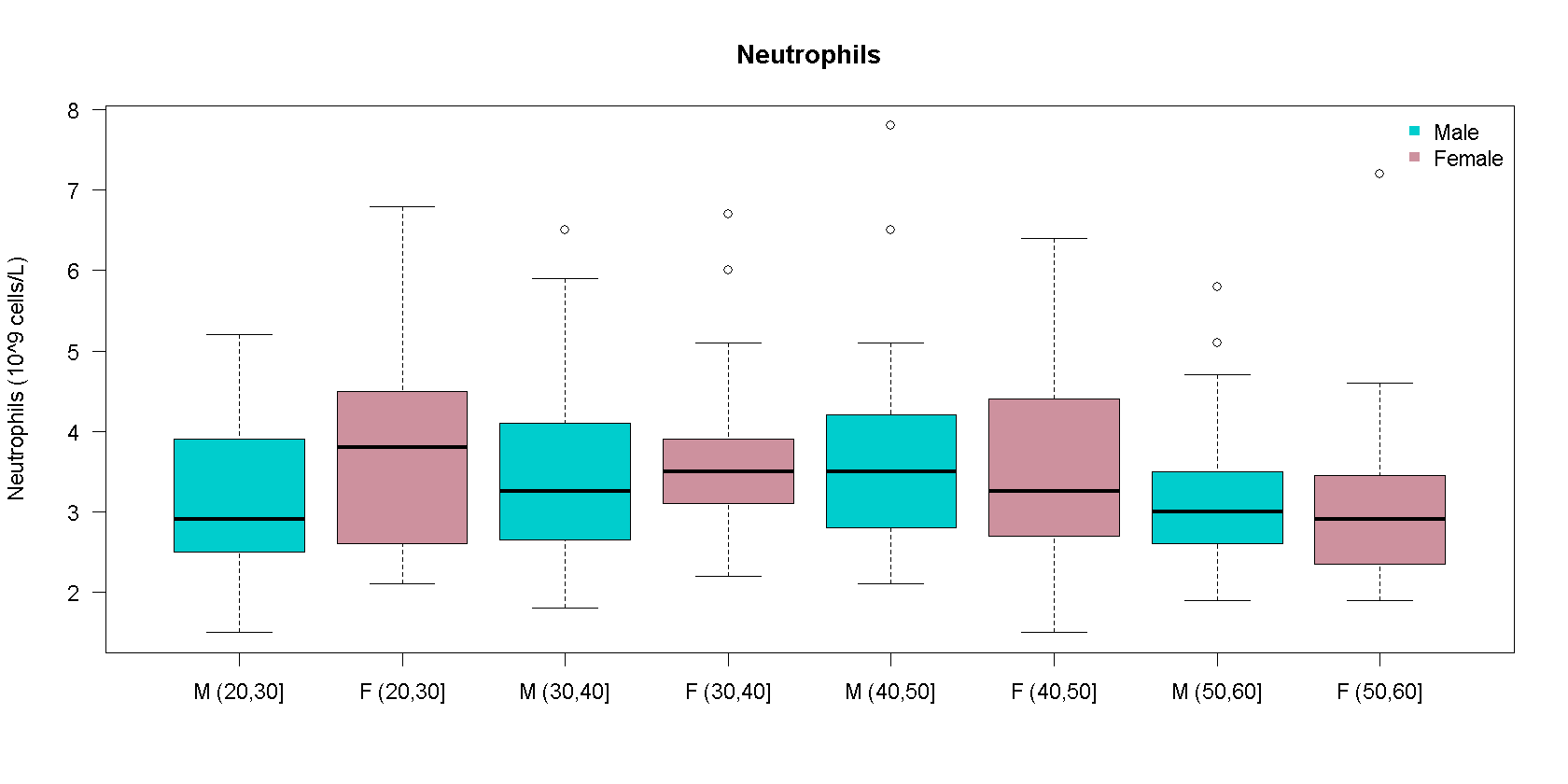


**Figure. Neutrophils**. Boxplot

**Table.** Mean value and standard deviation of Neutrophils variable. Neutrophils (1.9-8 10^9 celss/L)

|  | Male | Female |
| --- | --- | --- |
| (20,30] | 3.22$\pm$ (0.951) | 3.86$\pm$ (1.38) |
| (30,40] | 3.49$\pm$ (1.16) | 3.61$\pm$ (0.951) |
| (40,50] | 3.68$\pm$ (1.17) | 3.54$\pm$ (1.15) |
| (50,60] | 3.19$\pm$ (0.861) | 3.13$\pm$ (1.17) |

**Table.** Two-way ANOVA analysis for Neutrophils variable by gender and age. Neutrophils (1.9-8 10^9 cells/L)

|  | Df | Sum Sq | Mean Sq | F value | Pr(>F) |
| --- | --- | --- | --- | --- | --- |
| Age | 3 | 7.430228 | 2.476743 | 2.1024857 | 0.1003731 |
| Gender | 1 | 0.604095 | 0.604095 | 0.5128111 | 0.4745798 |
| Age:Gender | 3 | 4.974352 | 1.658117 | 1.4075617 | 0.2410335 |
| Residuals | 255 | 300.391761 | 1.178007 |  |  |

#### Analysis of variable Lymphocytes (%)


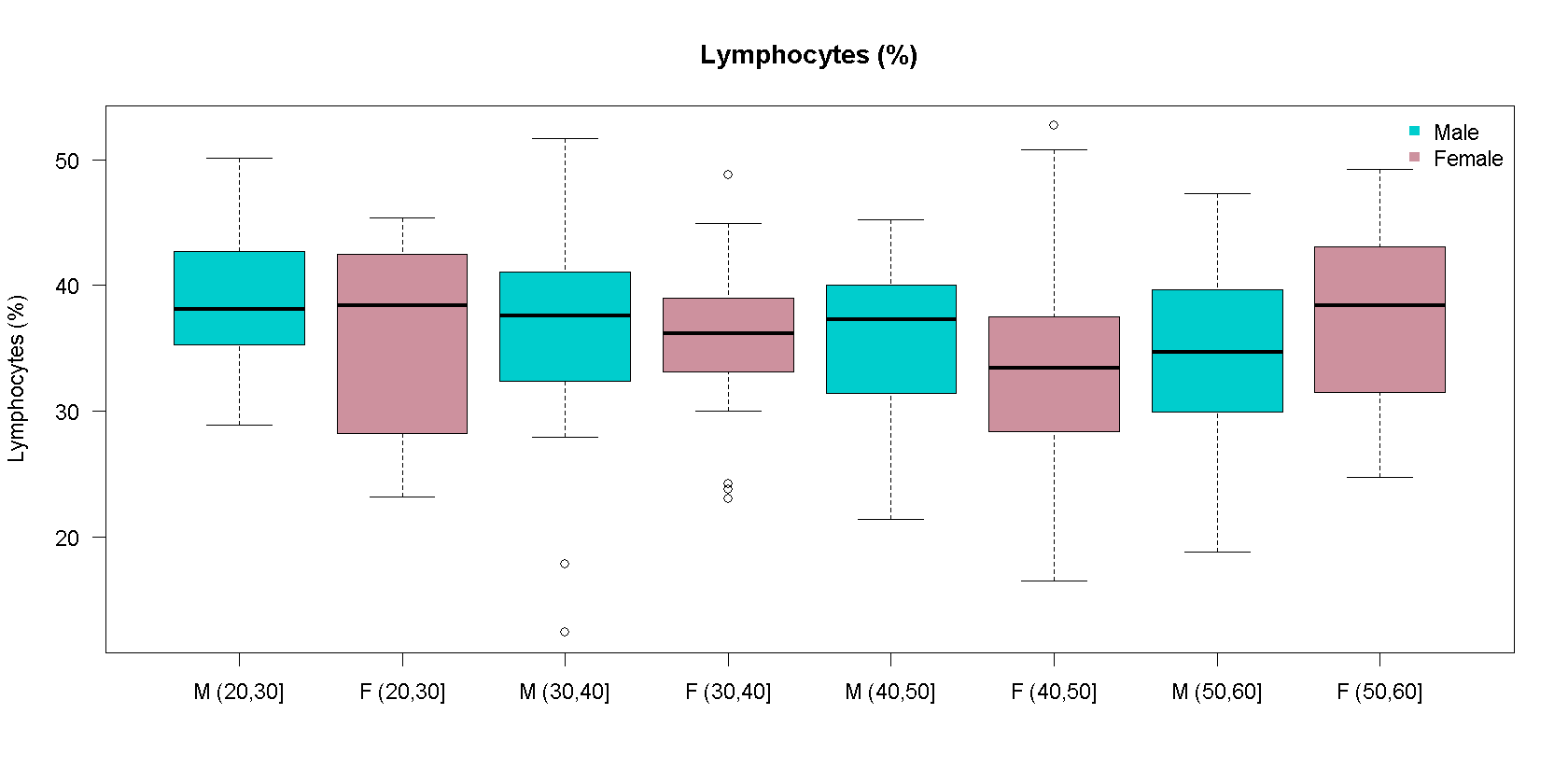


**Figure. Lymphocytes (%)**. Boxplot

**Table.** Mean value and standard deviation of Lymphocytes (%) variable. Lymphocytes % (20-49)

|  | Male | Female |
| --- | --- | --- |
| (20,30] | 38.6$\pm$ (5.46) | 35.9$\pm$ (7.64) |
| (30,40] | 36.2$\pm$ (7.72) | 35.8$\pm$ (5.55) |
| (40,50] | 35.7$\pm$ (5.84) | 33.6$\pm$ (7.62) |
| (50,60] | 34.8$\pm$ (6.68) | 37.3$\pm$ (7.26) |

**Table.** Two-way ANOVA analysis for Lymphocytes (%) variable by gender and age. Lymphocytes % (20-49)

|  | Df | Sum Sq | Mean Sq | F value | Pr(>F) |
| --- | --- | --- | --- | --- | --- |
| Age | 3 | 303.99163 | 101.33054 | 2.2700247 | 0.0808824 |
| Gender | 1 | 35.08422 | 35.08422 | 0.7859628 | 0.3761590 |
| Age:Gender | 3 | 235.50428 | 78.50143 | 1.7586028 | 0.1555643 |
| Residuals | 255 | 11382.82266 | 44.63852 |  |  |

#### Analysis of variable Lymphocytes


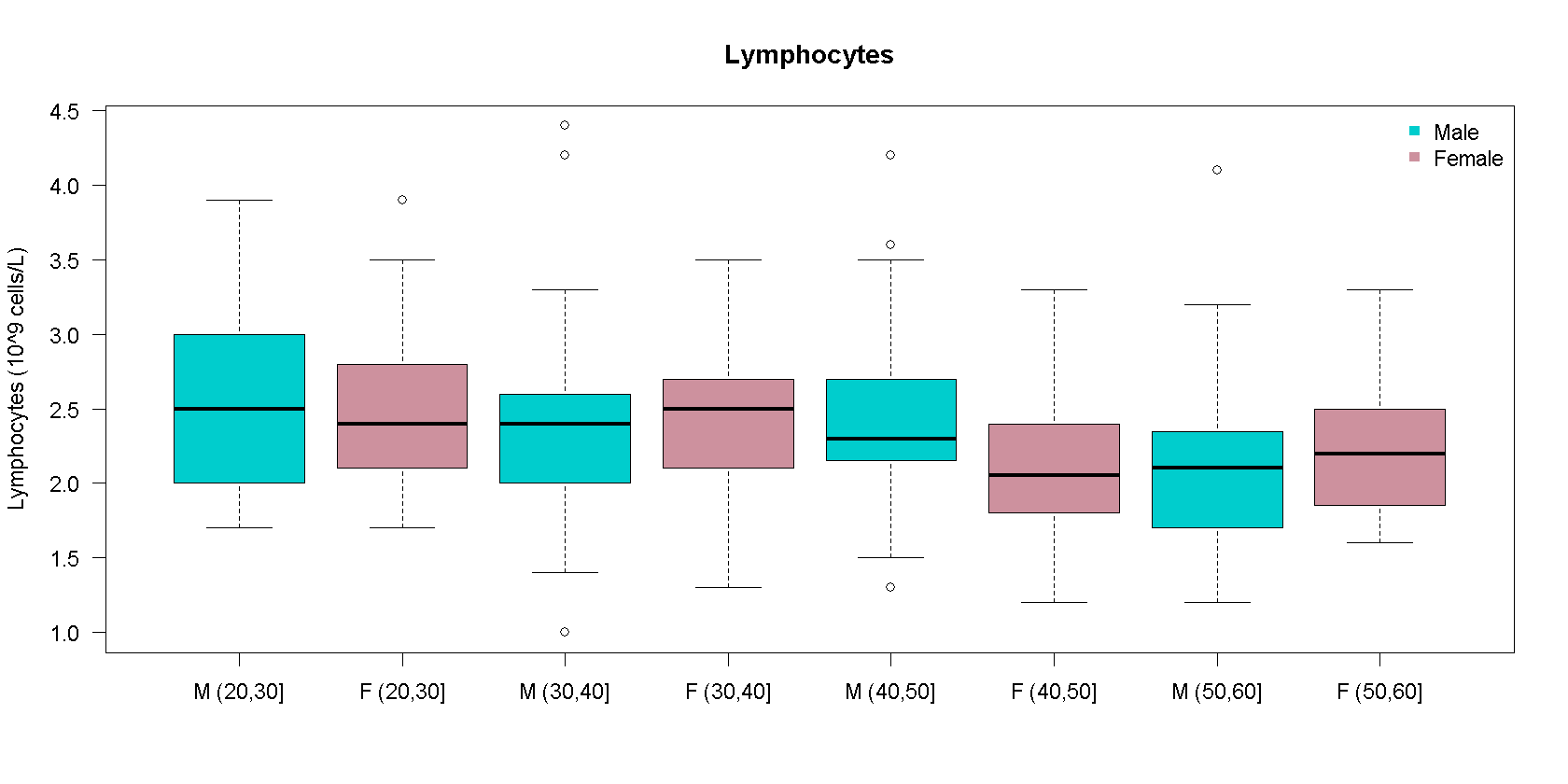


**Figure. Lymphocytes**. Boxplot

**Table.** Mean value and standard deviation of Lymphocytes variable. Lymphocytes (0.9-4 10^9 cells/L)

|  | Male | Female |
| --- | --- | --- |
| (20,30] | 2.54$\pm$ (0.565) | 2.47$\pm$ (0.576) |
| (30,40] | 2.41$\pm$ (0.668) | 2.42$\pm$ (0.462) |
| (40,50] | 2.44$\pm$ (0.572) | 2.1$\pm$ (0.463) |
| (50,60] | 2.17$\pm$ (0.672) | 2.23$\pm$ (0.434) |

**Table.** Two-way ANOVA analysis for Lymphocytes variable by gender and age. Lymphocytes (0.9-4 10^9 cells/L)

|  | Df | Sum Sq | Mean Sq | F value | Pr(>F) |
| --- | --- | --- | --- | --- | --- |
| Age | 3 | 3.7210357 | 1.2403452 | 3.937469 | 0.0090132 |
| Gender | 1 | 0.6142056 | 0.6142056 | 1.949792 | 0.1638236 |
| Age:Gender | 3 | 1.7901260 | 0.5967087 | 1.894248 | 0.1309912 |
| Residuals | 255 | 80.3277605 | 0.3150108 |  |  |

#### Analysis of variable Monocytes (%)


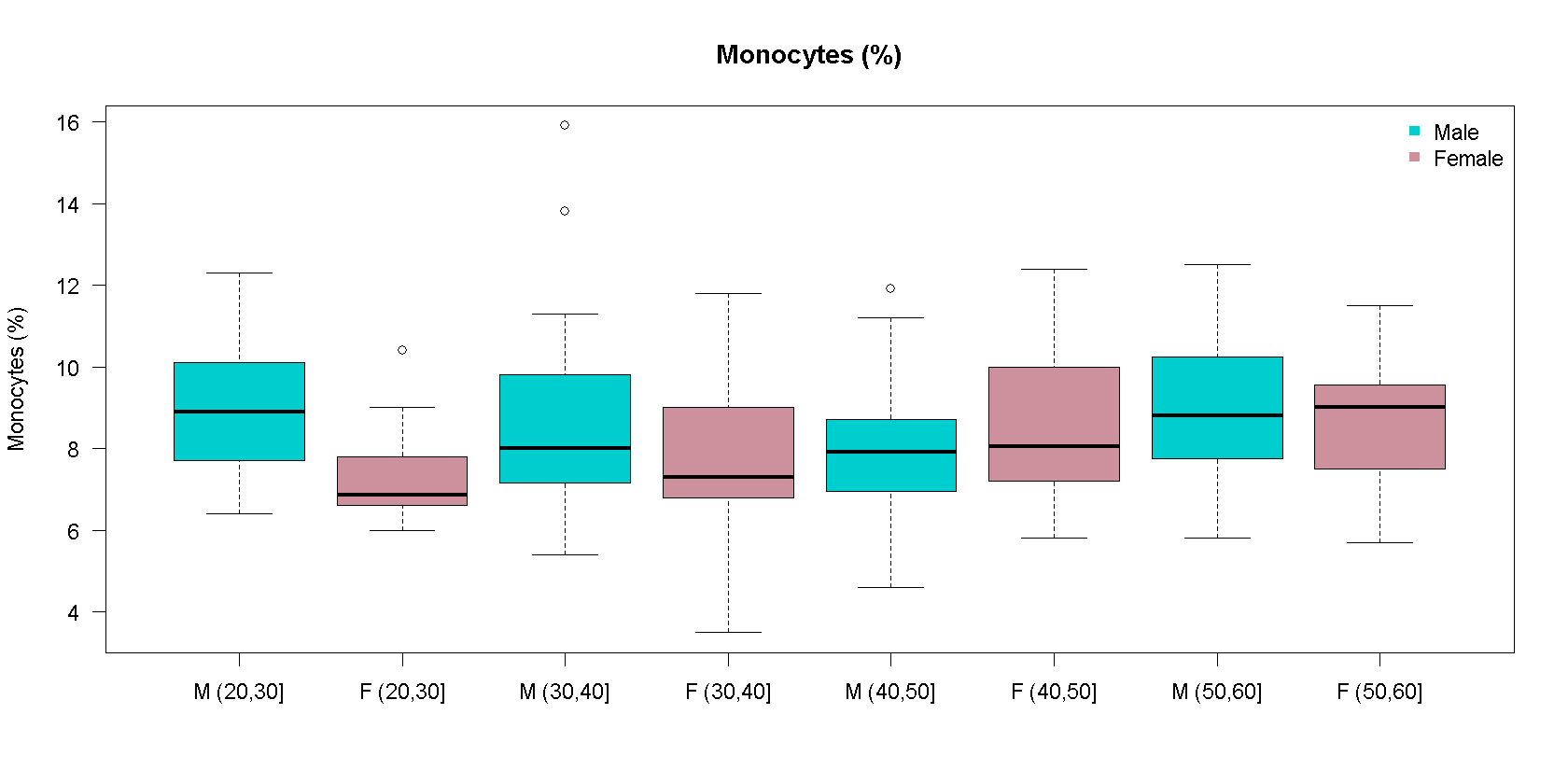


**Figure. Monocytes (%)**. Boxplot

**Table.** Mean value and standard deviation of Monocytes (%) variable. Monocytes % (2-14%)

|  | Male | Female |
| --- | --- | --- |
| (20,30] | 9.07$\pm$ (1.5) | 7.32$\pm$ (1.1) |
| (30,40] | 8.54$\pm$ (2.1) | 7.82$\pm$ (1.91) |
| (40,50] | 8.08$\pm$ (1.81) | 8.53$\pm$ (1.81) |
| (50,60] | 9.07$\pm$ (1.79) | 8.72$\pm$ (1.62) |

**Table.** Two-way ANOVA analysis for Monocytes (%) variable by gender and age. Monocytes % (2-14%)

|  | Df | Sum Sq | Mean Sq | F value | Pr(>F) |
| --- | --- | --- | --- | --- | --- |
| Age | 3 | 20.99679 | 6.998929 | 2.229468 | 0.0852325 |
| Gender | 1 | 14.60549 | 14.605486 | 4.652493 | 0.0319432 |
| Age:Gender | 3 | 37.46521 | 12.488403 | 3.978109 | 0.0085391 |
| Residuals | 255 | 800.51680 | 3.139282 |  |  |

#### Analysis of variable Monocytes


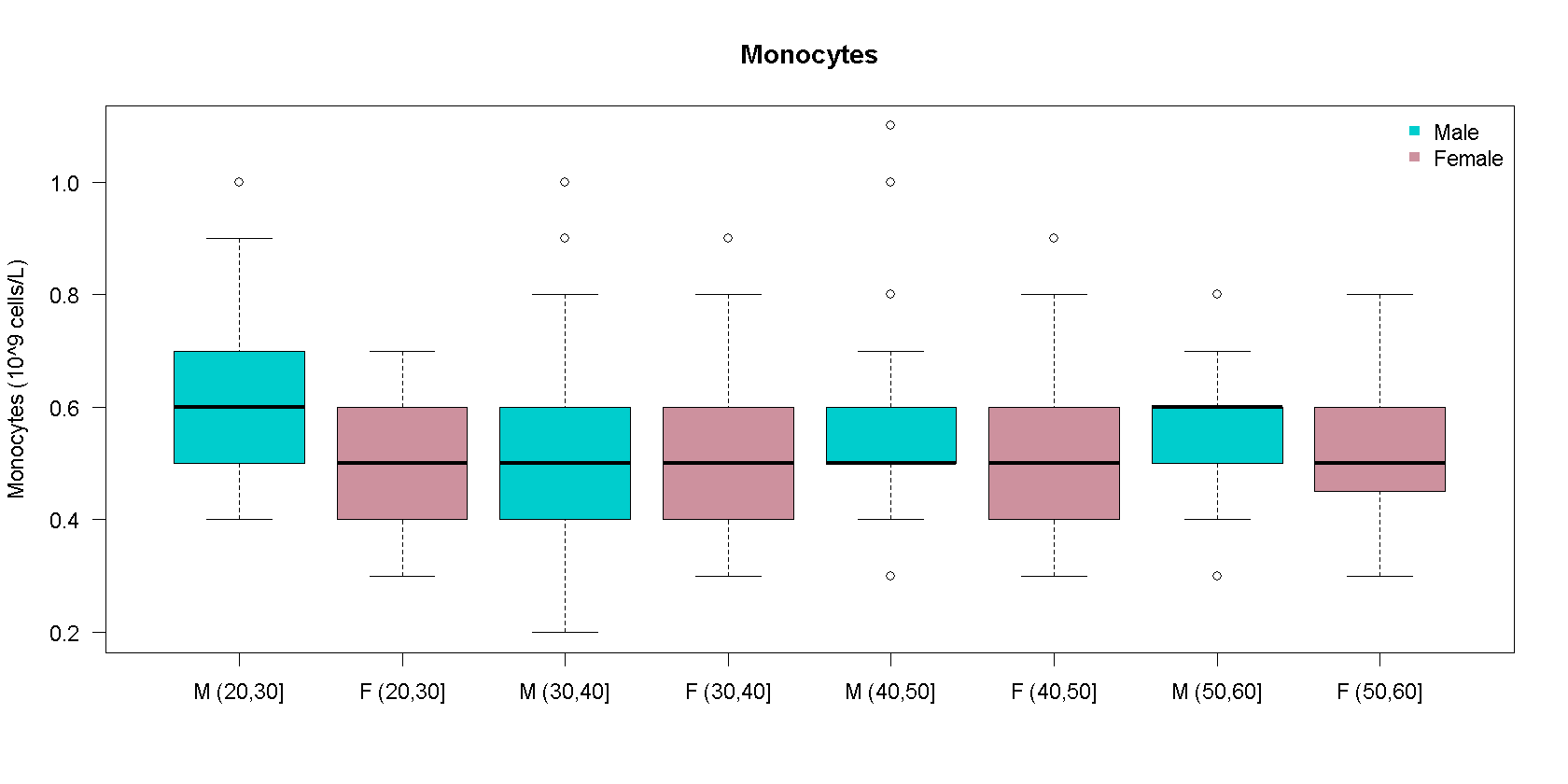


**Figure. Monocytes**. Boxplot

**Table.** Mean value and standard deviation of Monocytes variable. Monocytes (0.1-1 10^9 cells/L)

|  | Male | Female |
| --- | --- | --- |
| (20,30] | 0.603$\pm$ (0.167) | 0.506$\pm$ (0.121) |
| (30,40] | 0.553$\pm$ (0.181) | 0.524$\pm$ (0.138) |
| (40,50] | 0.559$\pm$ (0.18) | 0.544$\pm$ (0.148) |
| (50,60] | 0.557$\pm$ (0.124) | 0.53$\pm$ (0.129) |

**Table.** Two-way ANOVA analysis for Monocytes variable by gender and age. Monocytes (0.1-1 10^9 cells/L)

|  | Df | Sum Sq | Mean Sq | F value | Pr(>F) |
| --- | --- | --- | --- | --- | --- |
| Age | 3 | 0.0346159 | 0.0115386 | 0.4874084 | 0.6913231 |
| Gender | 1 | 0.0879591 | 0.0879591 | 3.7155205 | 0.0550195 |
| Age:Gender | 3 | 0.0555702 | 0.0185234 | 0.7824559 | 0.5046741 |
| Residuals | 255 | 6.0367208 | 0.0236734 |  |  |

#### Analysis of variable Eosinophils (%)


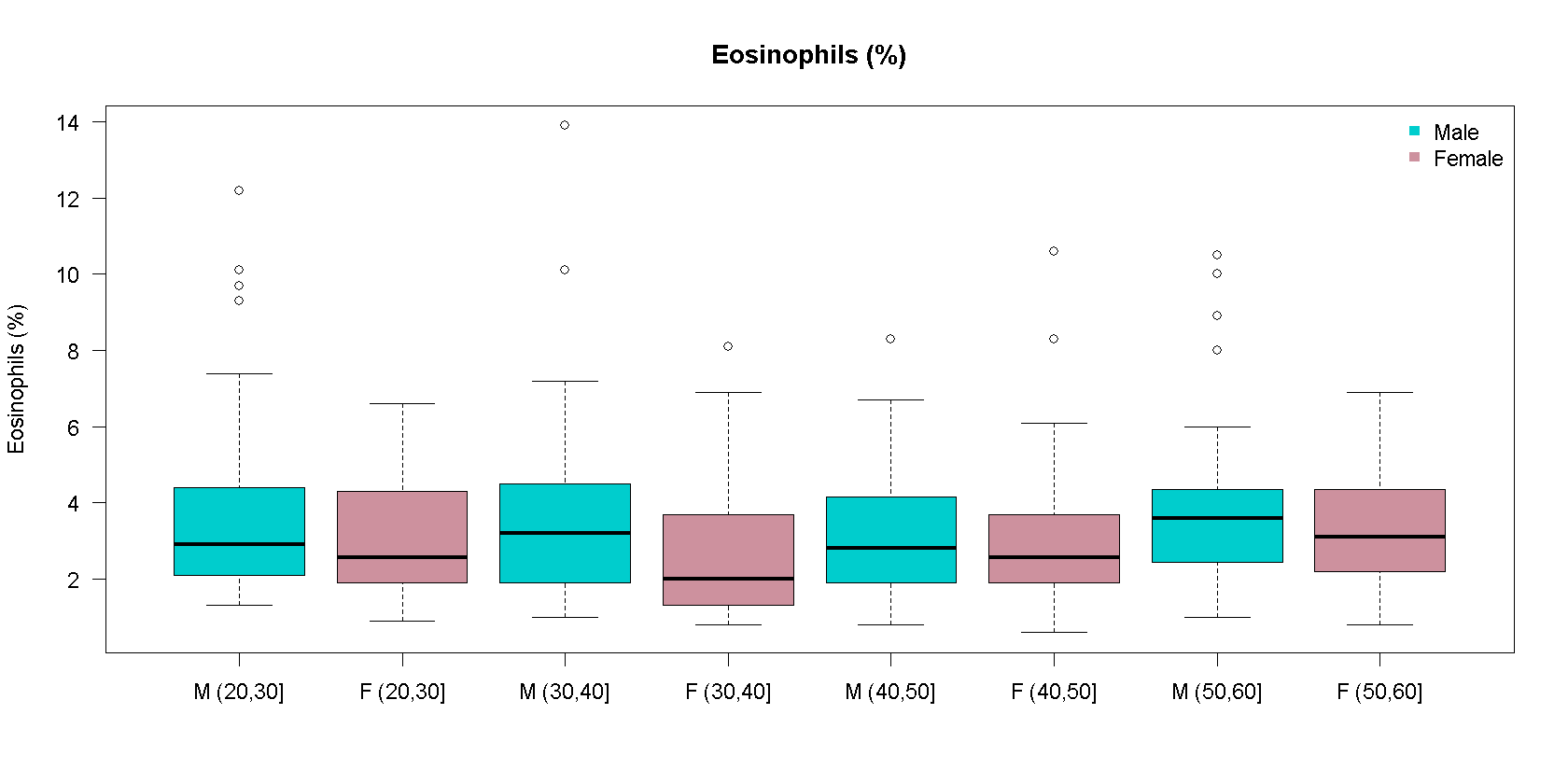


**Figure. Eosinophils (%)**. Boxplot

**Table.** Mean value and standard deviation of Eosinophils (%) variable. Eosinophils % (0-8%)

|  | Male | Female |
| --- | --- | --- |
| (20,30] | 3.84$\pm$ (2.65) | 3.02$\pm$ (1.63) |
| (30,40] | 3.72$\pm$ (2.6) | 2.78$\pm$ (2.01) |
| (40,50] | 3.24$\pm$ (1.86) | 3.07$\pm$ (2.01) |
| (50,60] | 3.99$\pm$ (2.28) | 3.26$\pm$ (1.59) |

**Table.** Two-way ANOVA analysis for Eosinophils (%) variable by gender and age. Eosinophils % (0-8%)

|  | Df | Sum Sq | Mean Sq | F value | Pr(>F) |
| --- | --- | --- | --- | --- | --- |
| Age | 3 | 13.248100 | 4.416033 | 0.9432173 | 0.4202883 |
| Gender | 1 | 26.036925 | 26.036925 | 5.5612076 | 0.0191181 |
| Age:Gender | 3 | 6.308873 | 2.102958 | 0.4491692 | 0.7181008 |
| Residuals | 255 | 1193.880236 | 4.681883 |  |  |

#### Analysis of variable Eosinophils


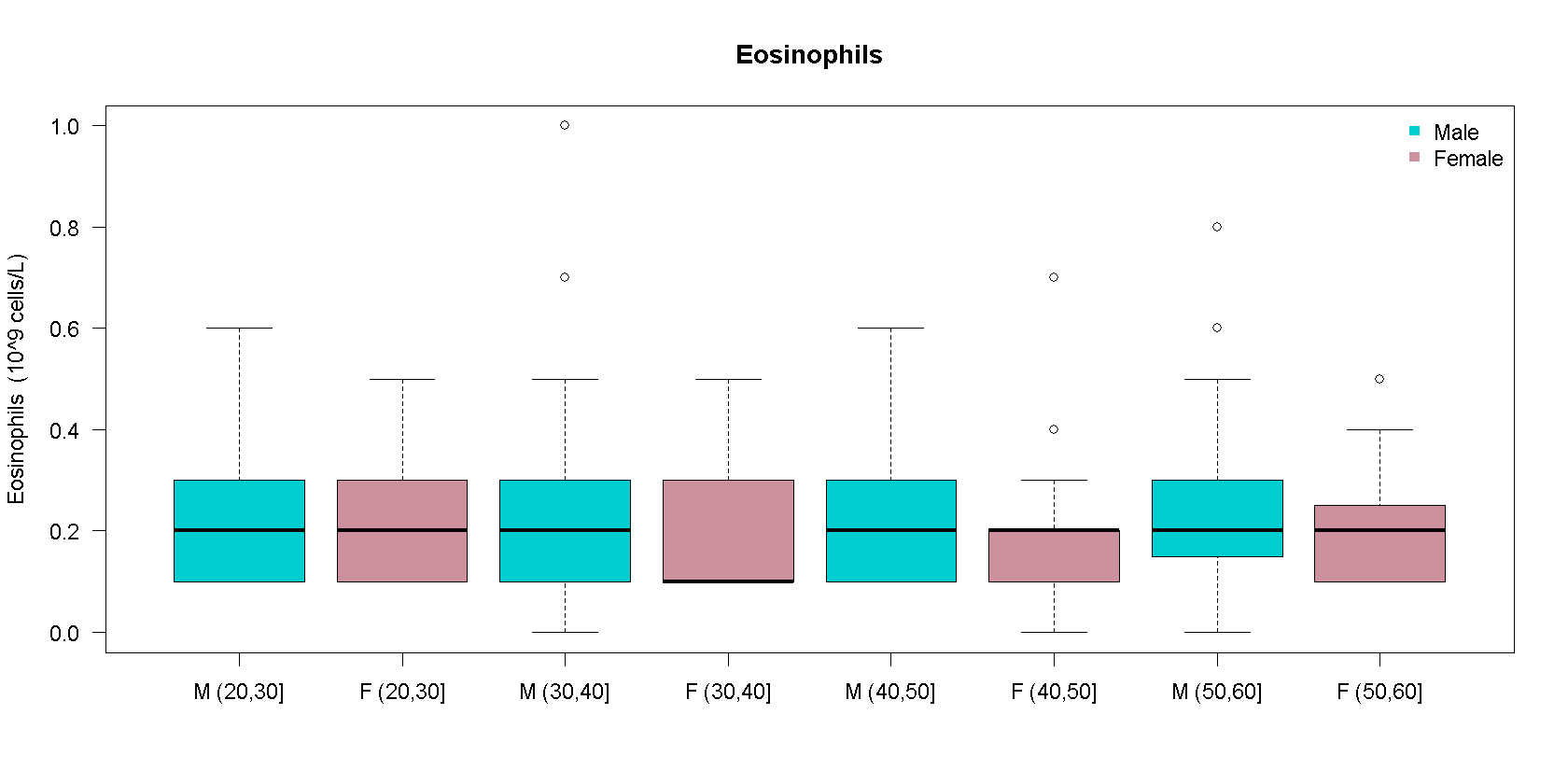


**Figure. Eosinophils**. Boxplot

**Table.** Mean value and standard deviation of Eosinophils variable. Eosinophils (0-0.6 10^9 cells/L)

|  | Male | Female |
| --- | --- | --- |
| (20,30] | 0.241$\pm$ (0.155) | 0.211$\pm$ (0.123) |
| (30,40] | 0.242$\pm$ (0.187) | 0.195$\pm$ (0.127) |
| (40,50] | 0.228$\pm$ (0.126) | 0.195$\pm$ (0.134) |
| (50,60] | 0.249$\pm$ (0.165) | 0.213$\pm$ (0.11) |

**Table.** Two-way ANOVA analysis for Eosinophils variable by gender and age. Eosinophils (0-0.6 10^9 cells/L)

|  | Df | Sum Sq | Mean Sq | F value | Pr(>F) |
| --- | --- | --- | --- | --- | --- |
| Age | 3 | 0.0223162 | 0.0074387 | 0.3522109 | 0.7875810 |
| Gender | 1 | 0.0862873 | 0.0862873 | 4.0855535 | 0.0442961 |
| Age:Gender | 3 | 0.0029070 | 0.0009690 | 0.0458812 | 0.9869335 |
| Residuals | 255 | 5.3856286 | 0.0211201 |  |  |

#### Analysis of variable Basophils (%)


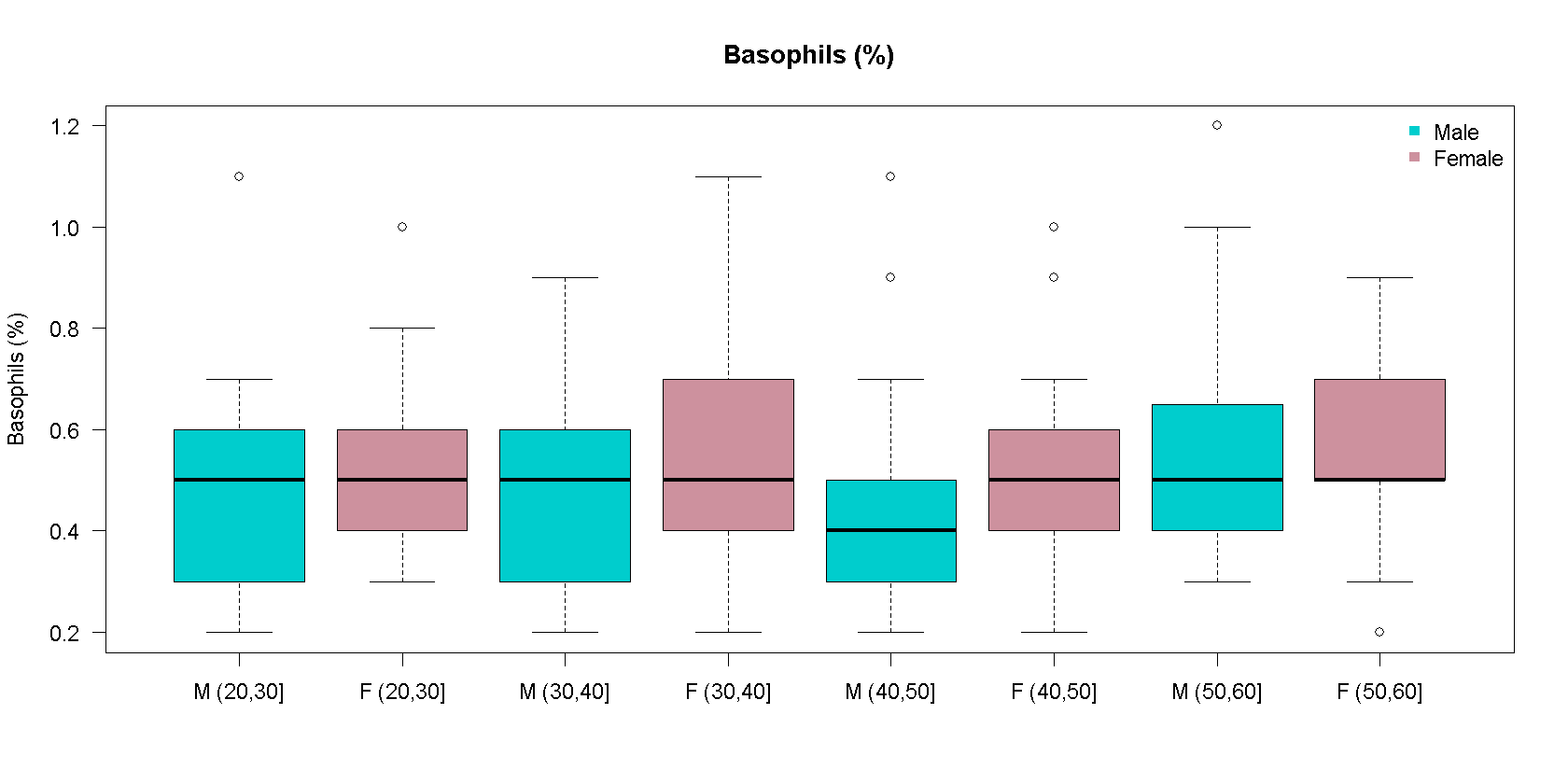


**Figure. Basophils (%)**. Boxplot

**Table.** Mean value and standard deviation of Basophils (%) variable. Basophils % (0-3%)

|  | Male | Female |
| --- | --- | --- |
| (20,30] | 0.473$\pm$ (0.184) | 0.556$\pm$ (0.169) |
| (30,40] | 0.475$\pm$ (0.161) | 0.532$\pm$ (0.229) |
| (40,50] | 0.451$\pm$ (0.18) | 0.511$\pm$ (0.181) |
| (50,60] | 0.571$\pm$ (0.24) | 0.561$\pm$ (0.175) |

**Table.** Two-way ANOVA analysis for Basophils (%) variable by gender and age. Basophils % (0-3%)

|  | Df | Sum Sq | Mean Sq | F value | Pr(>F) |
| --- | --- | --- | --- | --- | --- |
| Age | 3 | 0.2646010 | 0.0882003 | 2.3513105 | 0.0728046 |
| Gender | 1 | 0.1460440 | 0.1460440 | 3.8933495 | 0.0495575 |
| Age:Gender | 3 | 0.0662055 | 0.0220685 | 0.5883186 | 0.6231693 |
| Residuals | 255 | 9.5653409 | 0.0375111 |  |  |

#### Analysis of variable Basophils


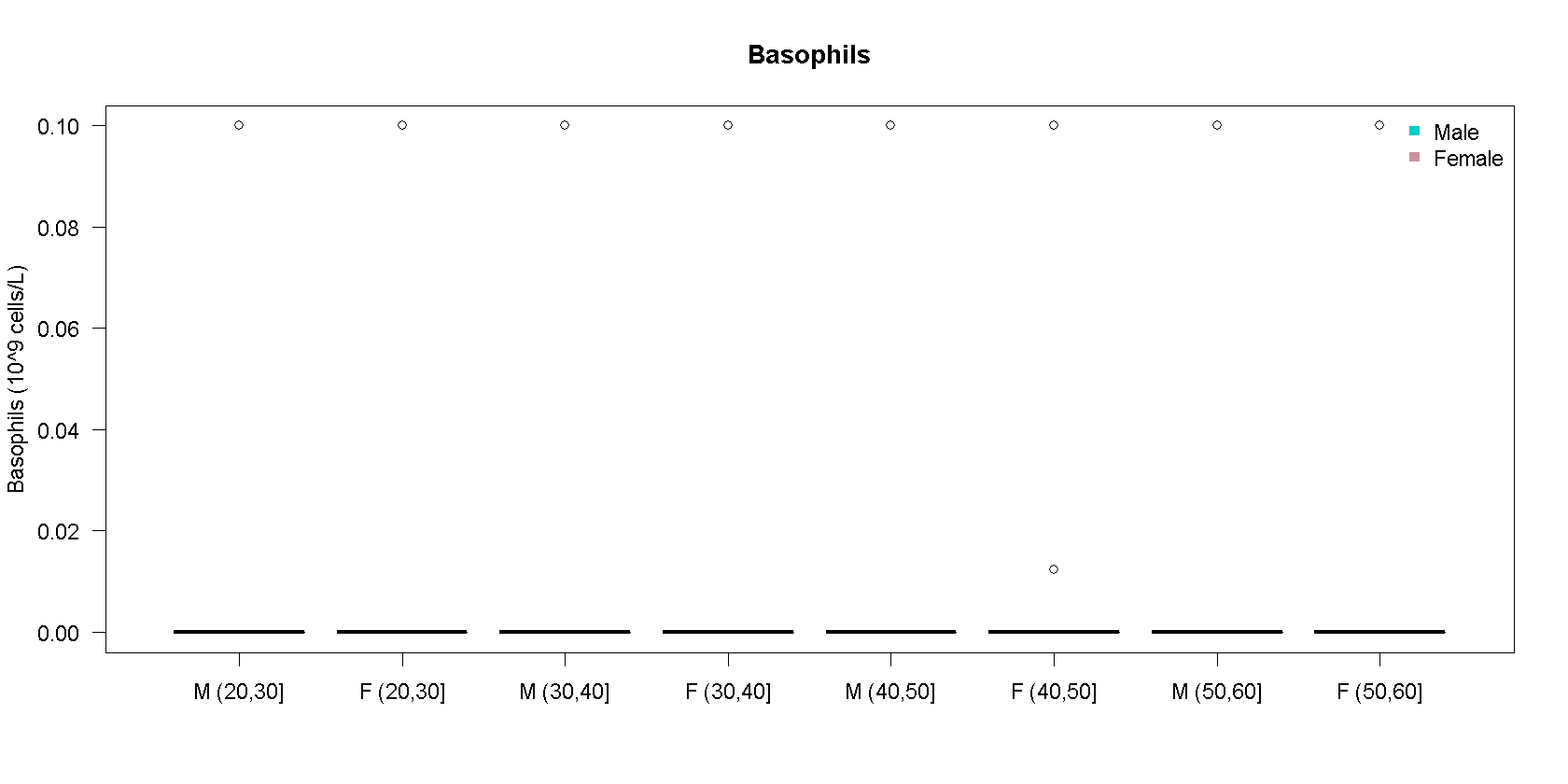


**Figure. Basophils**. Boxplot

**Table.** Mean value and standard deviation of Basophils variable. Basophils (0-0.2 10^9 cells/L)

|  | Male | Female |
| --- | --- | --- |
| (20,30] | 0.0135$\pm$ (0.0347) | 0.0222$\pm$ (0.0428) |
| (30,40] | 0.00833$\pm$ (0.028) | 0.0108$\pm$ (0.0315) |
| (40,50] | 0.00769$\pm$ (0.027) | 0.0108$\pm$ (0.0311) |
| (50,60] | 0.0143$\pm$ (0.0355) | 0.0174$\pm$ (0.0388) |

**Table.** Two-way ANOVA analysis for Basophils variable by gender and age. Basophils (0-0.2 10^9 cells/L)

|  | Df | Sum Sq | Mean Sq | F value | Pr(>F) |
| --- | --- | --- | --- | --- | --- |
| Age | 3 | 0.0027592 | 0.0009197 | 0.8473011 | 0.4691304 |
| Gender | 1 | 0.0010205 | 0.0010205 | 0.9401536 | 0.3331571 |
| Age:Gender | 3 | 0.0003355 | 0.0001118 | 0.1030401 | 0.9582248 |
| Residuals | 255 | 0.2768007 | 0.0010855 |  |  |

#### Analysis of variable Erythrocytes


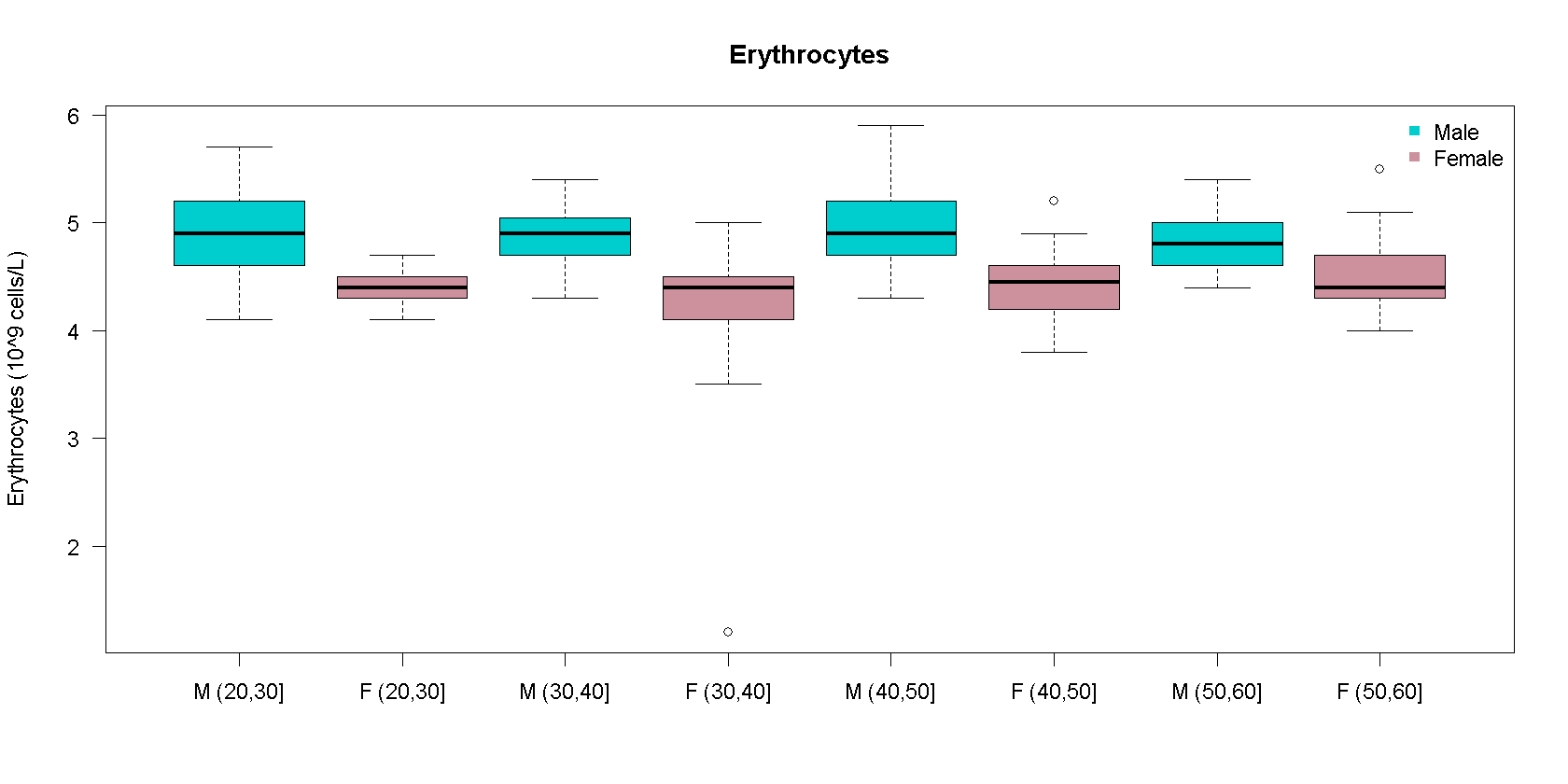


**Figure. Erythrocytes**. Boxplot

**Table.** Mean value and standard deviation of Erythrocytes variable. Erythrocytes (3.9-5.3 10^9 cells/L)

|  | Male | Female |
| --- | --- | --- |
| (20,30] | 4.89$\pm$ (0.401) | 4.38$\pm$ (0.172) |
| (30,40] | 4.86$\pm$ (0.264) | 4.26$\pm$ (0.6) |
| (40,50] | 4.97$\pm$ (0.356) | 4.43$\pm$ (0.295) |
| (50,60] | 4.83$\pm$ (0.274) | 4.51$\pm$ (0.353) |

**Table.** Two-way ANOVA analysis for Erythrocytes variable by gender and age. Erythrocytes (3.9-5.3 10^9 cells/L)

|  | Df | Sum Sq | Mean Sq | F value | Pr(>F) |
| --- | --- | --- | --- | --- | --- |
| Age | 3 | 1.2332791 | 0.4110930 | 2.990123 | 0.0315899 |
| Gender | 1 | 15.9728332 | 15.9728332 | 116.179856 | 0.0000000 |
| Age:Gender | 3 | 0.6744384 | 0.2248128 | 1.635196 | 0.1816836 |
| Residuals | 255 | 35.0583363 | 0.1374837 |  |  |

#### Analysis of variable Hemoglobin


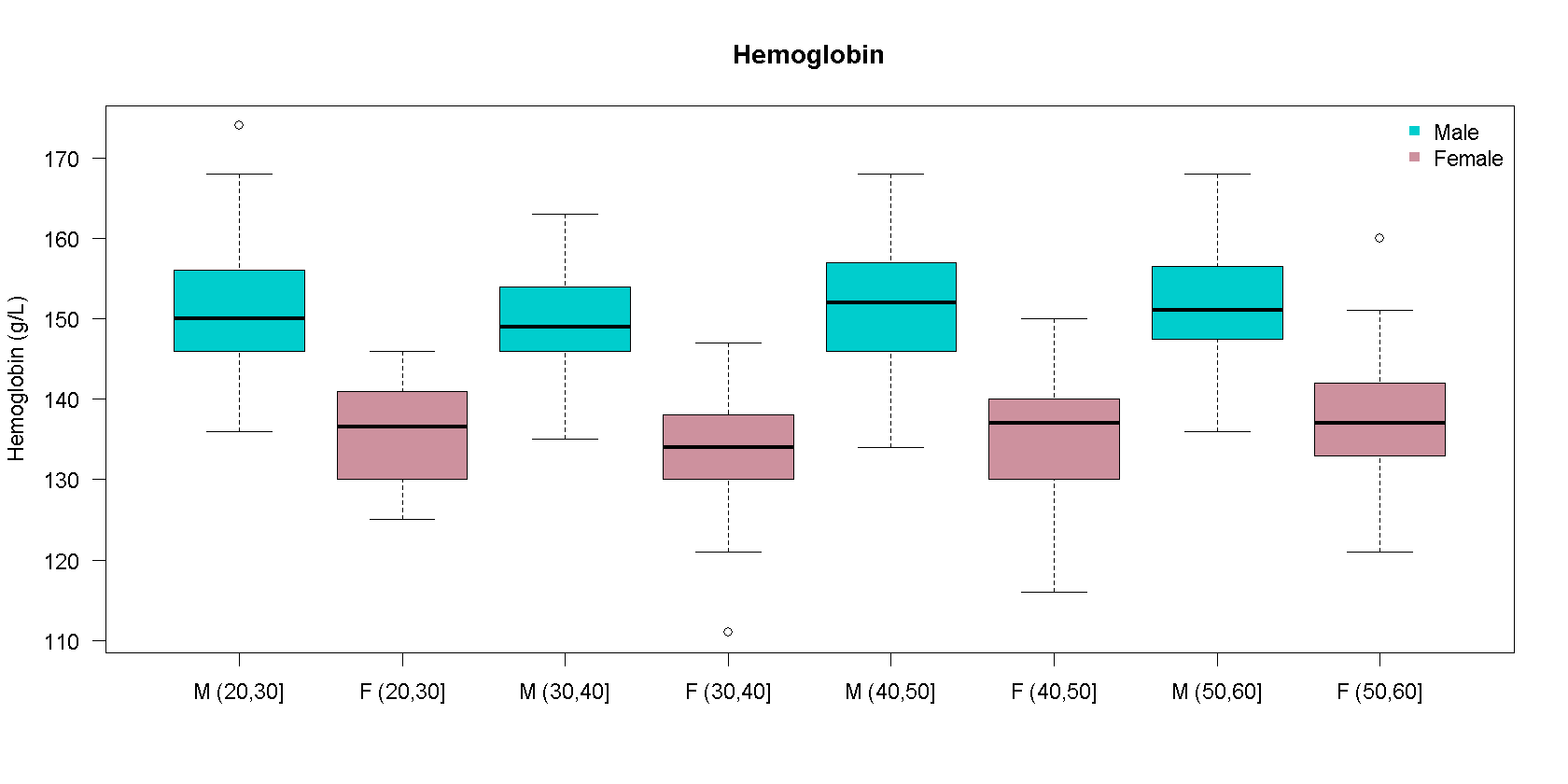


**Figure. Hemoglobin**. Boxplot

**Table.** Mean value and standard deviation of Hemoglobin variable. Hemoglobin Female (118-160 g/L) Male (135-175 g/L)

|  | Male | Female |
| --- | --- | --- |
| (20,30] | 151$\pm$ (8.16) | 136$\pm$ (5.71) |
| (30,40] | 150$\pm$ (6.2) | 133$\pm$ (7.22) |
| (40,50] | 152$\pm$ (8.12) | 135$\pm$ (7.78) |
| (50,60] | 151$\pm$ (7.35) | 138$\pm$ (8.42) |

**Table.** Two-way ANOVA analysis for Hemoglobin variable by gender and age. Hemoglobin Female (118-160 g/L) Male (135-175 g/L)

|  | Df | Sum Sq | Mean Sq | F value | Pr(>F) |
| --- | --- | --- | --- | --- | --- |
| Age | 3 | 1135.1228 | 378.37425 | 6.7391883 | 0.0002161 |
| Gender | 1 | 15476.7434 | 15476.74344 | 275.6548230 | 0.0000000 |
| Age:Gender | 3 | 114.1389 | 38.04631 | 0.6776392 | 0.5664470 |
| Residuals | 255 | 14317.0707 | 56.14538 |  |  |

#### Analysis of variable Hematocrit


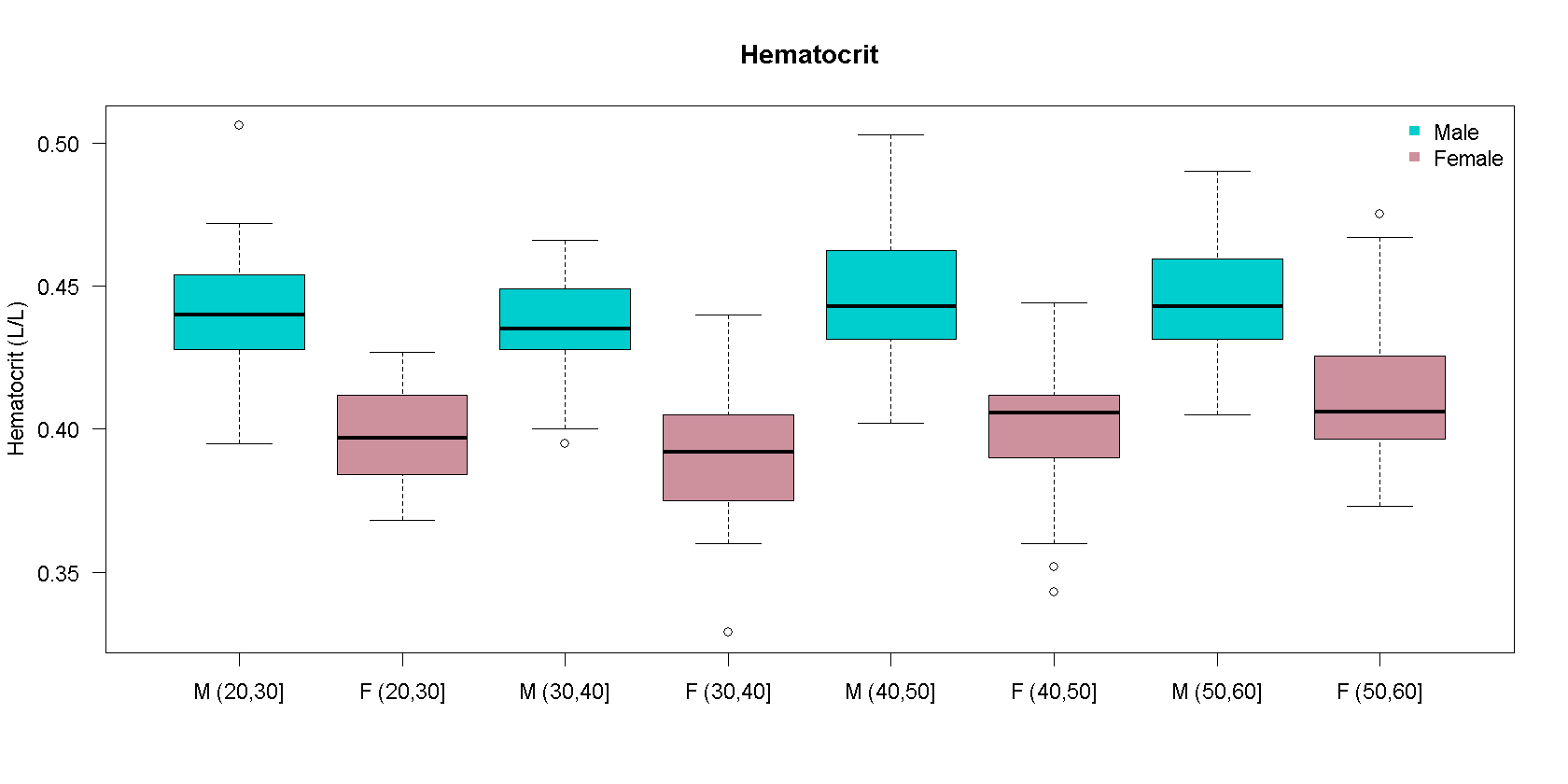


**Figure. Hematocrit**. Boxplot

**Table.** Mean value and standard deviation of Hematocrit variable. Hematocrit Female (0.4-0.53 L/L) Male (0.365-0.47L/L)

|  | Male | Female |
| --- | --- | --- |
| (20,30] | 0.44$\pm$ (0.0242) | 0.399$\pm$ (0.0176) |
| (30,40] | 0.437$\pm$ (0.0171) | 0.391$\pm$ (0.0223) |
| (40,50] | 0.447$\pm$ (0.0243) | 0.401$\pm$ (0.0229) |
| (50,60] | 0.444$\pm$ (0.0218) | 0.414$\pm$ (0.0279) |

**Table.** Two-way ANOVA analysis for Hematocrit variable by gender and age. Hematocrit Female (0.4-0.53 L/L) Male (0.365-0.47L/L)

|  | Df | Sum Sq | Mean Sq | F value | Pr(>F) |
| --- | --- | --- | --- | --- | --- |
| Age | 3 | 0.0112092 | 0.0037364 | 7.359655 | 0.0000949 |
| Gender | 1 | 0.1099111 | 0.1099111 | 216.494860 | 0.0000000 |
| Age:Gender | 3 | 0.0026966 | 0.0008989 | 1.770500 | 0.1532442 |
| Residuals | 255 | 0.1294596 | 0.0005077 |  |  |

#### Analysis of variable Mean Corpuscular Volumen (MCV)


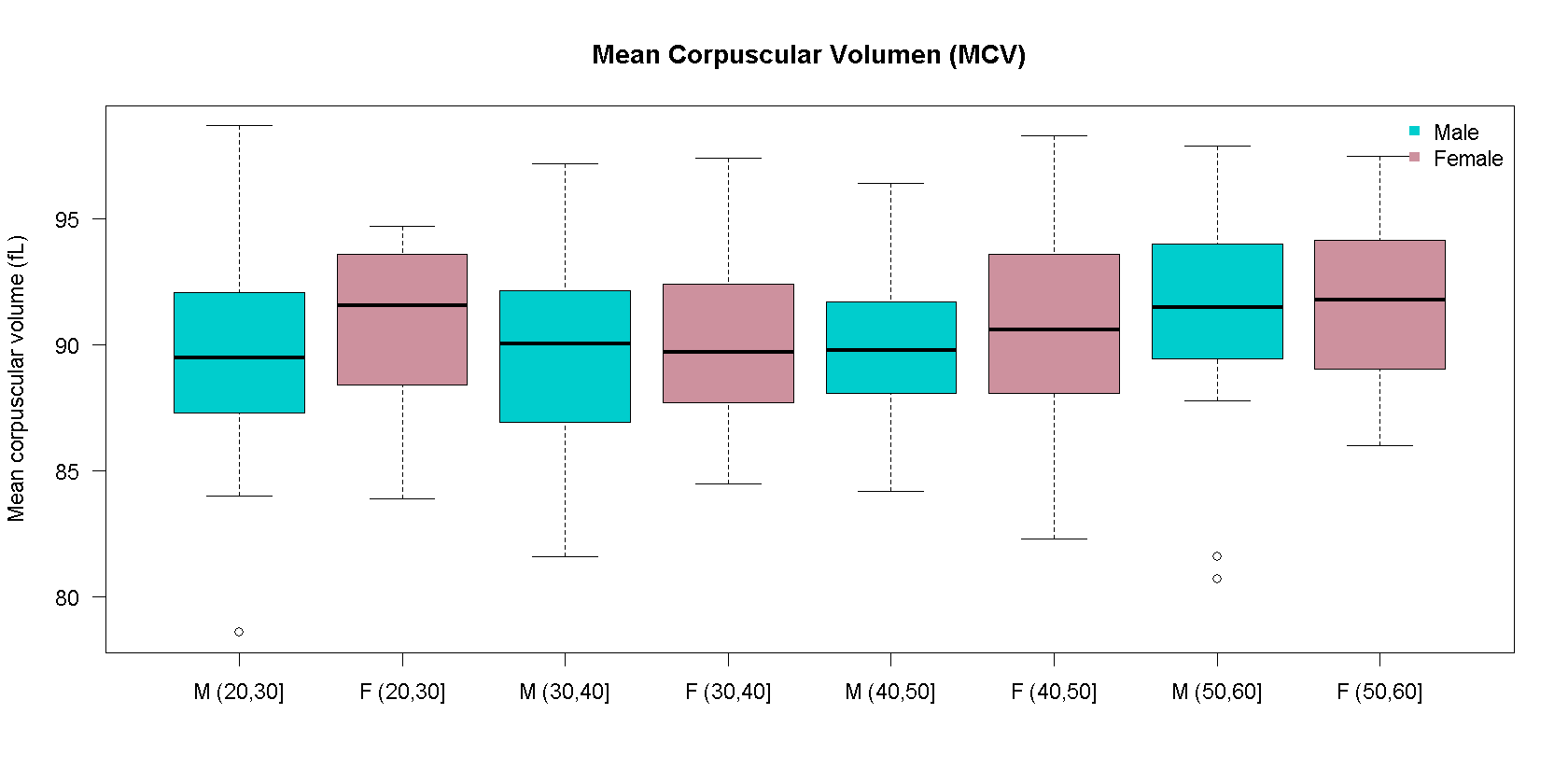


**Figure. Mean Corpuscular Volumen (MCV)**. Boxplot

**Table.** Mean value and standard deviation of Mean Corpuscular Volumen (MCV) variable. Mean corpuscular volume (MCV) (80-99 fL)

|  | Male | Female |
| --- | --- | --- |
| (20,30] | 89.6$\pm$ (4.12) | 90.6$\pm$ (3.47) |
| (30,40] | 89.7$\pm$ (3.41) | 90.1$\pm$ (2.88) |
| (40,50] | 89.8$\pm$ (3.11) | 90.3$\pm$ (3.98) |
| (50,60] | 91.4$\pm$ (3.64) | 91.5$\pm$ (3.34) |

**Table.** Two-way ANOVA analysis for Mean Corpuscular Volumen (MCV) variable by gender and age. Mean corpuscular volume (MCV) (80-99 fL)

|  | Df | Sum Sq | Mean Sq | F value | Pr(>F) |
| --- | --- | --- | --- | --- | --- |
| Age | 3 | 101.743568 | 33.914522 | 2.7267061 | 0.0446396 |
| Gender | 1 | 13.253598 | 13.253598 | 1.0655809 | 0.3029238 |
| Age:Gender | 3 | 6.026193 | 2.008731 | 0.1615007 | 0.9221765 |
| Residuals | 255 | 3171.666802 | 12.437909 |  |  |

#### Analysis of variable Mean Corpuscular Hemoglobin (MCH)


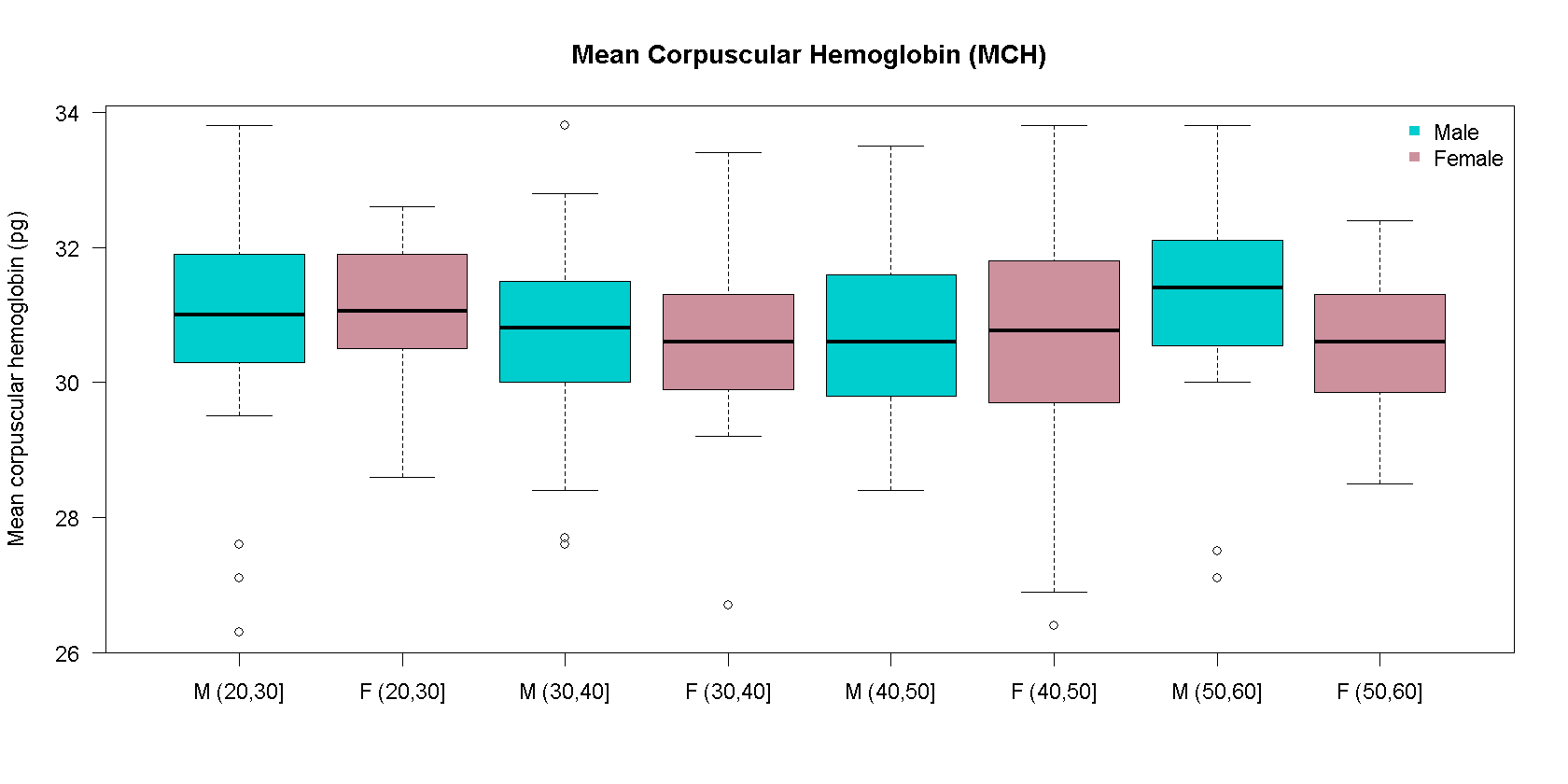


**Figure. Mean Corpuscular Hemoglobin (MCH)**. Boxplot

**Table.** Mean value and standard deviation of Mean Corpuscular Hemoglobin (MCH) variable. Mean corpuscular hemoglobin (MCH) (27-34.5 pg)

|  | Male | Female |
| --- | --- | --- |
| (20,30] | 30.8$\pm$ (1.56) | 30.9$\pm$ (1.27) |
| (30,40] | 30.7$\pm$ (1.35) | 30.6$\pm$ (1.23) |
| (40,50] | 30.7$\pm$ (1.24) | 30.6$\pm$ (1.64) |
| (50,60] | 31.3$\pm$ (1.37) | 30.6$\pm$ (1.12) |

**Table.** Two-way ANOVA analysis for Mean Corpuscular Hemoglobin (MCH) variable by gender and age. Mean corpuscular hemoglobin (MCH) (27-34.5 pg)

|  | Df | Sum Sq | Mean Sq | F value | Pr(>F) |
| --- | --- | --- | --- | --- | --- |
| Age | 3 | 4.883151 | 1.627717 | 0.8604158 | 0.4621907 |
| Gender | 1 | 2.857035 | 2.857035 | 1.5102365 | 0.2202359 |
| Age:Gender | 3 | 4.769851 | 1.589950 | 0.8404522 | 0.4727878 |
| Residuals | 255 | 482.403831 | 1.891780 |  |  |

#### Analysis of variable Mean corpuscular hemoglobin concentration (MCHC)


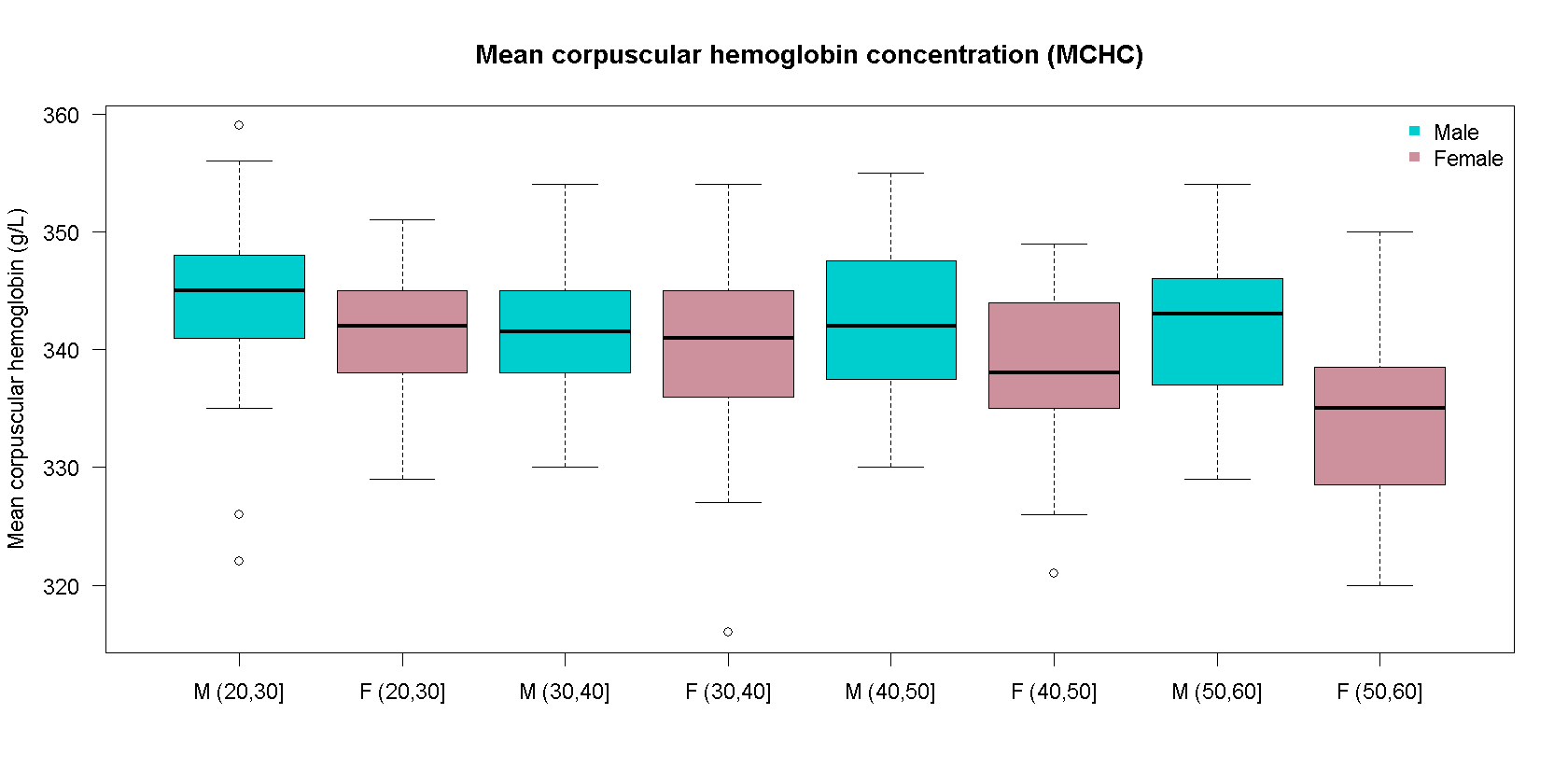


**Figure. Mean corpuscular hemoglobin concentration (MCHC)**. Boxplot

**Table.** Mean value and standard deviation of Mean corpuscular hemoglobin concentration (MCHC). Mean corpuscular hemoglobin concentration (MCHC) (320-360 g/L)

|  | Male | Female |
| --- | --- | --- |
| (20,30] | 344$\pm$ (7.59) | 341$\pm$ (5.76) |
| (30,40] | 342$\pm$ (5.17) | 340$\pm$ (7.55) |
| (40,50] | 342$\pm$ (6.19) | 338$\pm$ (6.51) |
| (50,60] | 342$\pm$ (5.76) | 334$\pm$ (7.9) |

**Table.** Two-way ANOVA analysis for Mean corpuscular hemoglobin concentration (MCHC) variable by gender and age. Mean corpuscular hemoglobin concentration (MCHC) (320-360 g/L)

|  | Df | Sum Sq | Mean Sq | F value | Pr(>F) |
| --- | --- | --- | --- | --- | --- |
| Age | 3 | 568.0349 | 189.34495 | 4.330529 | 0.0053419 |
| Gender | 1 | 1018.5393 | 1018.53930 | 23.295122 | 0.0000024 |
| Age:Gender | 3 | 262.5411 | 87.51372 | 2.001536 | 0.1142375 |
| Residuals | 255 | 11149.4383 | 43.72329 |  |  |

#### Analysis of variable Red Cell Distribution Width (RDW)


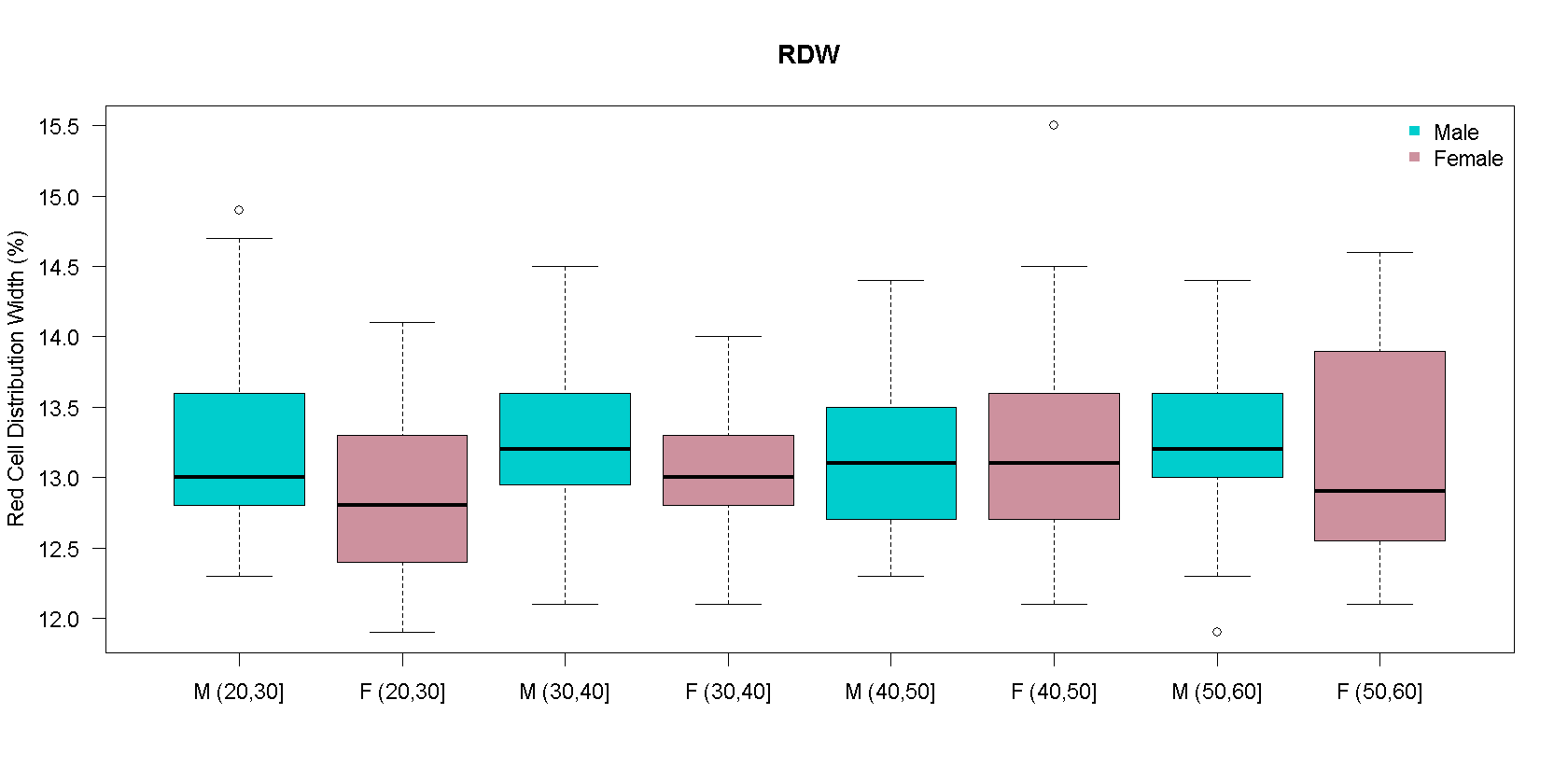


**Figure. Red Cell Distribution Width (RDW)**. Boxplot

**Table.** Mean value and standard deviation of RDW variable. RDW % (11-15%)

|  | Male | Female |
| --- | --- | --- |
| (20,30] | 13.2$\pm$ (0.624) | 12.9$\pm$ (0.652) |
| (30,40] | 13.3$\pm$ (0.587) | 13$\pm$ (0.467) |
| (40,50] | 13.1$\pm$ (0.521) | 13.2$\pm$ (0.696) |
| (50,60] | 13.3$\pm$ (0.575) | 13.2$\pm$ (0.814) |

**Table.** Two-way ANOVA analysis for RDW variable by gender and age. RDW % (11-15%)

|  | Df | Sum Sq | Mean Sq | F value | Pr(>F) |
| --- | --- | --- | --- | --- | --- |
| Age | 3 | 0.8156975 | 0.2718992 | 0.7280684 | 0.5360652 |
| Gender | 1 | 0.8656518 | 0.8656518 | 2.3179683 | 0.1291261 |
| Age:Gender | 3 | 1.0736129 | 0.3578710 | 0.9582763 | 0.4130184 |
| Residuals | 255 | 95.2304640 | 0.3734528 |  |  |

#### Analysis of variable Platelets


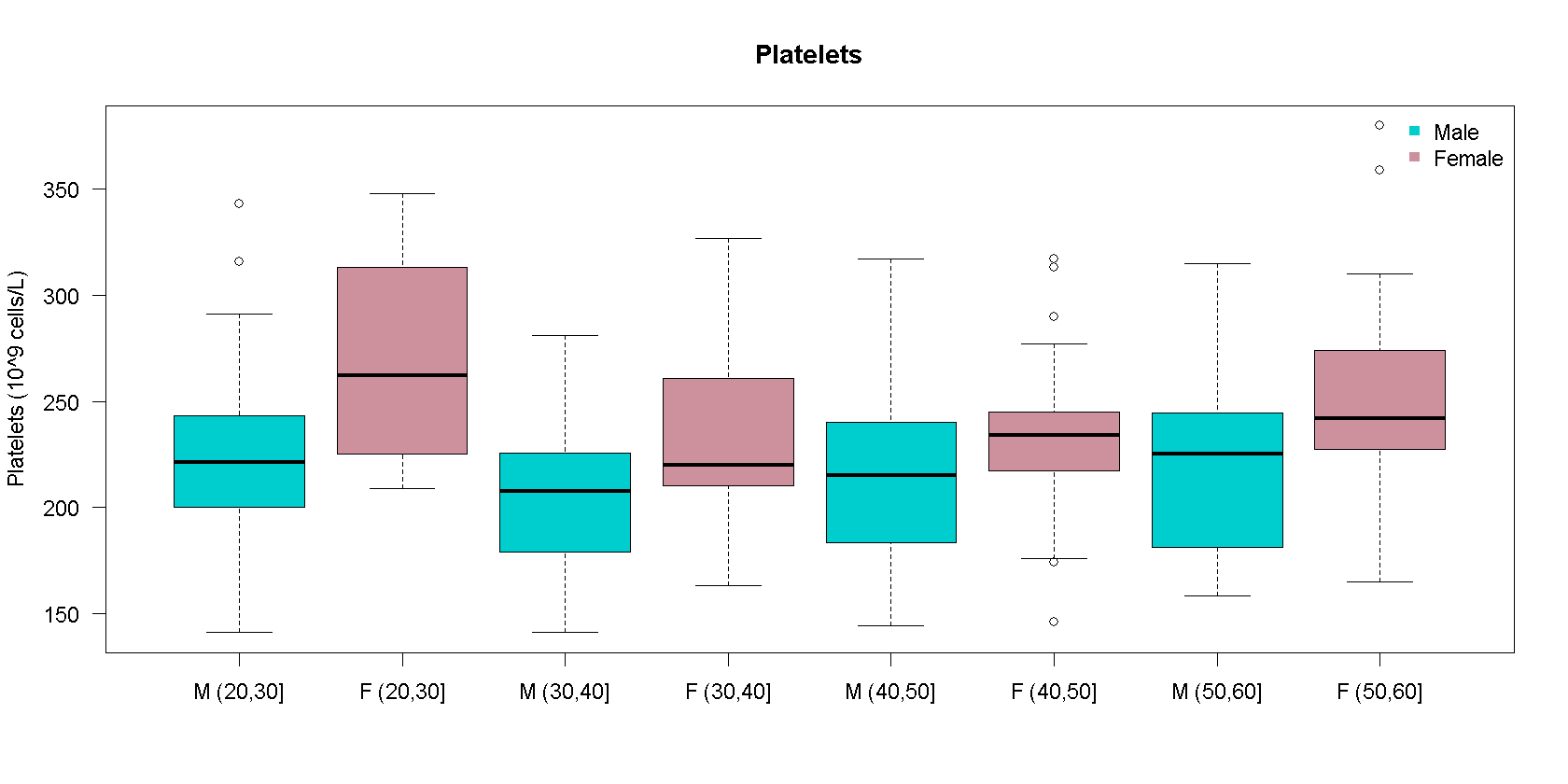


**Figure. Platelets**. Boxplot

**Table.** Mean value and standard deviation of Platelets variable.Platelets (150-400 10^9 cells/L)

|  | Male | Female |
| --- | --- | --- |
| (20,30] | 225$\pm$ (44.1) | 270$\pm$ (47.9) |
| (30,40] | 206$\pm$ (35.9) | 233$\pm$ (40.5) |
| (40,50] | 216$\pm$ (43.9) | 232$\pm$ (36.1) |
| (50,60] | 216$\pm$ (39.6) | 250$\pm$ (52.9) |

**Table.** Two-way ANOVA analysis for Platelets variable by gender and age. Platelets (150-400 10^9 cells/L)

|  | Df | Sum Sq | Mean Sq | F value | Pr(>F) |
| --- | --- | --- | --- | --- | --- |
| Age | 3 | 13598.58 | 4532.858 | 2.569643 | 0.0548099 |
| Gender | 1 | 52861.47 | 52861.473 | 29.966768 | 0.0000001 |
| Age:Gender | 3 | 6881.67 | 2293.890 | 1.300389 | 0.2748078 |
| Residuals | 255 | 449820.79 | 1764.003 |  |  |

#### Analysis of variable Mean platelet volume (MPV)


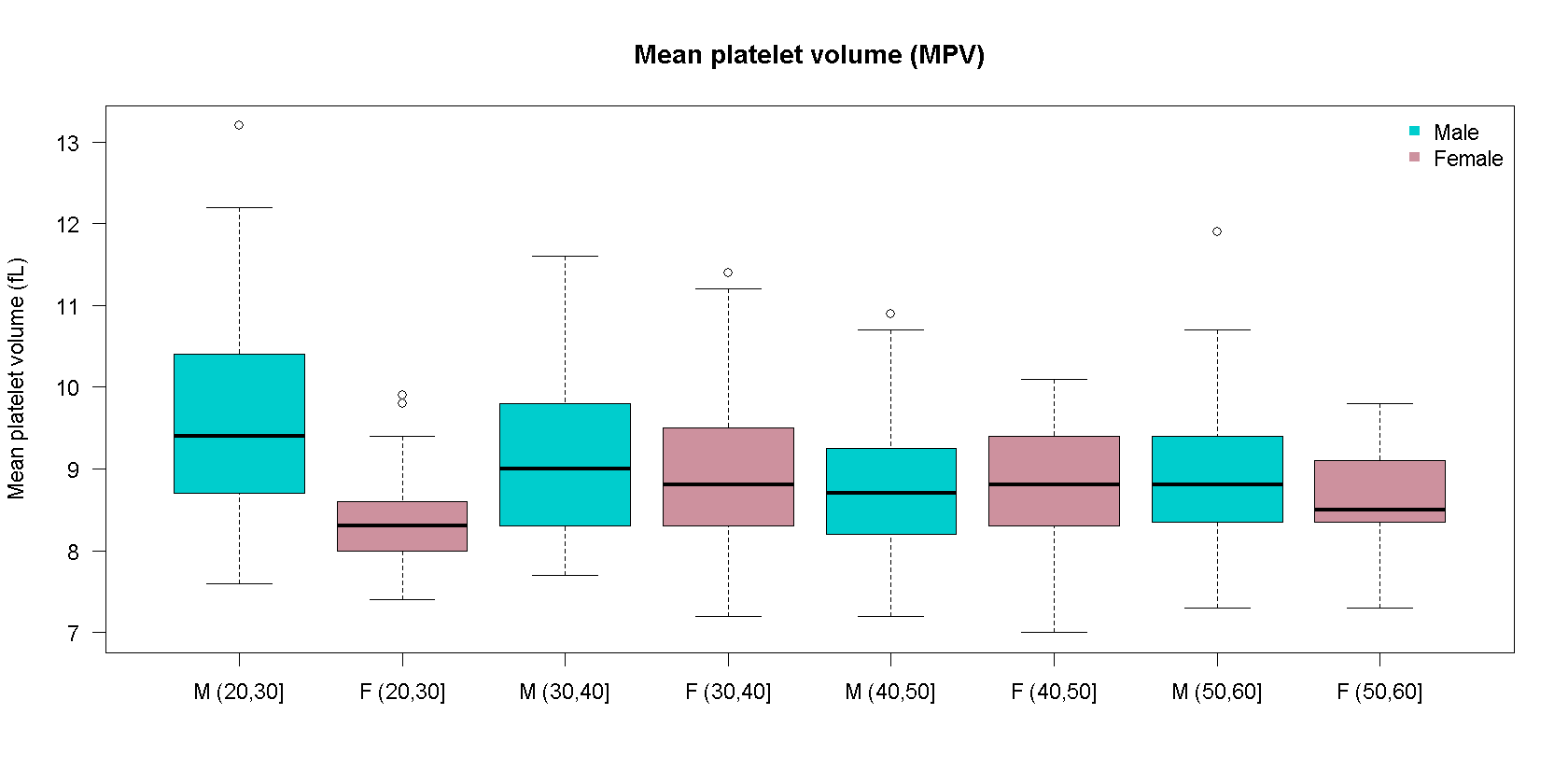


**Figure. Mean platelet volume (MPV)**. Boxplot

**Table.** Mean value and standard deviation of Mean platelet volume (MPV) variable. Mean platelet volume (MPV) (6.9-12 fL)

|  | Male | Female |
| --- | --- | --- |
| (20,30] | 9.66$\pm$ (1.35) | 8.44$\pm$ (0.729) |
| (30,40] | 9.14$\pm$ (1.01) | 9$\pm$ (1.04) |
| (40,50] | 8.77$\pm$ (0.877) | 8.83$\pm$ (0.717) |
| (50,60] | 8.88$\pm$ (0.946) | 8.65$\pm$ (0.716) |

**Table.** Two-way ANOVA analysis for Mean platelet volume (MPV) variable by gender and age. Mean platelet volume (MPV) (6.9-12 fL)

|  | Df | Sum Sq | Mean Sq | F value | Pr(>F) |
| --- | --- | --- | --- | --- | --- |
| Age | 3 | 9.591765 | 3.1972551 | 3.415424 | 0.0180186 |
| Gender | 1 | 5.989911 | 5.9899112 | 6.398640 | 0.0120240 |
| Age:Gender | 3 | 13.422799 | 4.4742664 | 4.779573 | 0.0029363 |
| Residuals | 255 | 238.711262 | 0.9361226 |  |  |

#### Analysis of variable Cholesterol


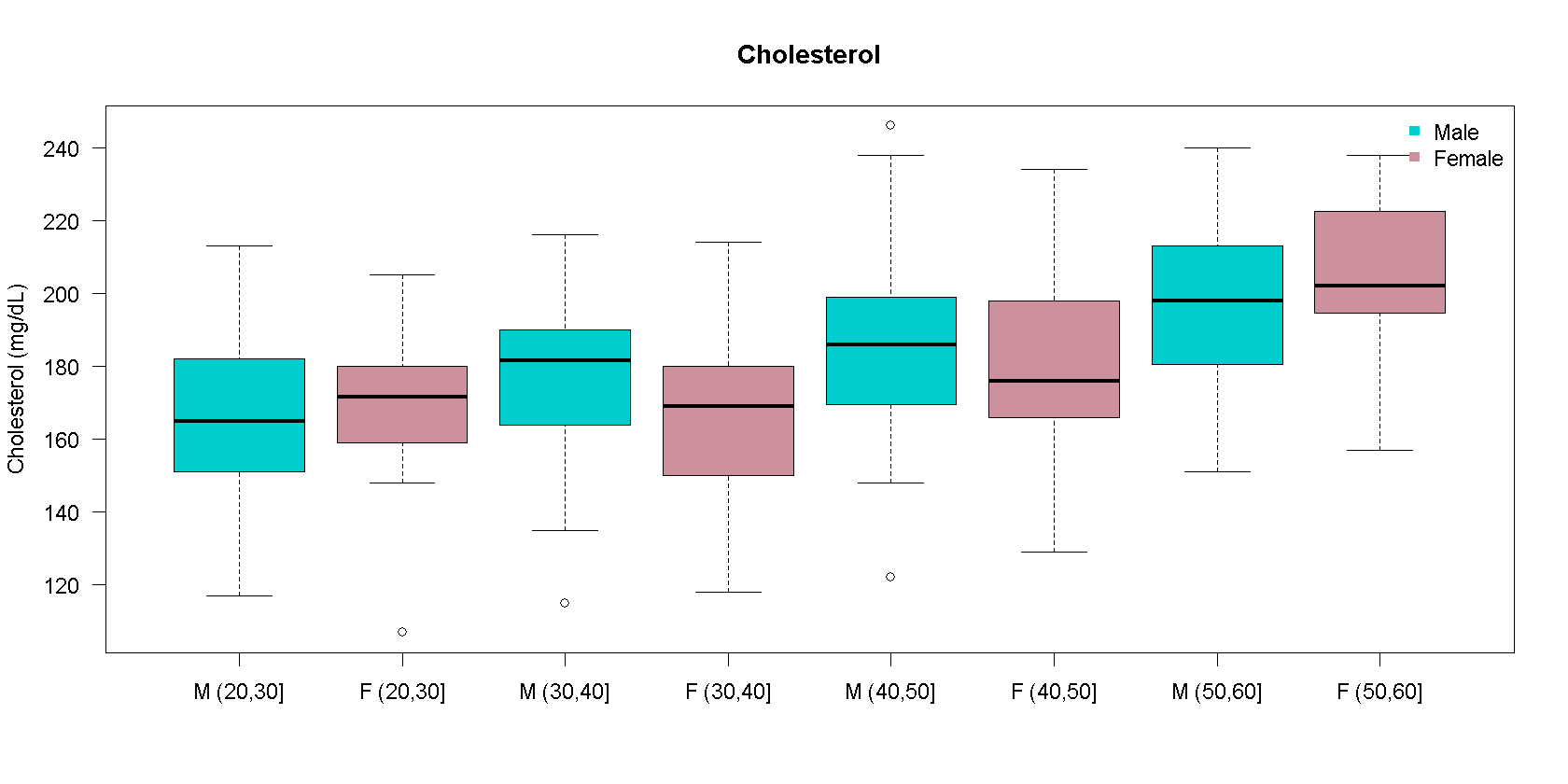


**Figure. Cholesterol**. Boxplot

**Table.** Mean value and standard deviation of Cholesterol variable. Cholesterol (<200 mg/dL)

|  | Male | Female |
| --- | --- | --- |
| (20,30] | 164$\pm$ (24.9) | 169$\pm$ (21.1) |
| (30,40] | 177$\pm$ (23.9) | 167$\pm$ (23.9) |
| (40,50] | 187$\pm$ (25.4) | 181$\pm$ (20.3) |
| (50,60] | 197$\pm$ (23) | 206$\pm$ (21.8) |

**Table.** Two-way ANOVA analysis for Cholesterol variable by gender and age. Cholesterol (<200 mg/dL)

|  | Df | Sum Sq | Mean Sq | F value | Pr(>F) |
| --- | --- | --- | --- | --- | --- |
| Age | 3 | 40606.1205 | 13535.3735 | 24.8550899 | 0.0000000 |
| Gender | 1 | 163.4442 | 163.4442 | 0.3001335 | 0.5842779 |
| Age:Gender | 3 | 3844.3588 | 1281.4529 | 2.3531399 | 0.0726322 |
| Residuals | 255 | 138865.7315 | 544.5715 |  |  |

#### Analysis of variable HDL Cholesterol


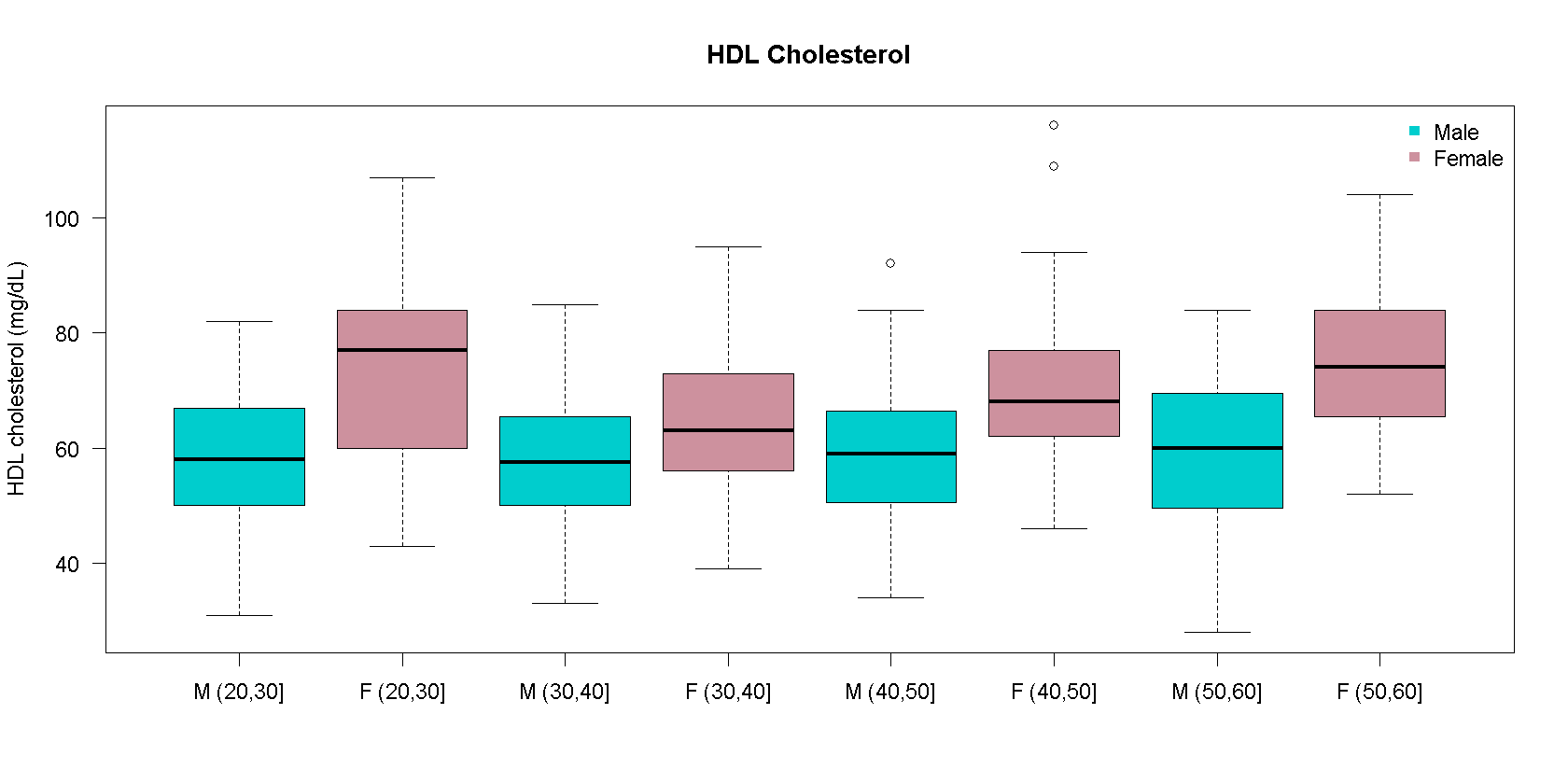


**Figure. HDL Cholesterol**. Boxplot

**Table.** Mean value and standard deviation of HDL Cholesterol variable. HDL cholesterol (>40 mg/dL)

|  | Male | Female |
| --- | --- | --- |
| (20,30] | 58$\pm$ (11.6) | 72.2$\pm$ (18.6) |
| (30,40] | 57.5$\pm$ (11.9) | 65.3$\pm$ (13.9) |
| (40,50] | 59.2$\pm$ (13) | 70.8$\pm$ (14.8) |
| (50,60] | 60.1$\pm$ (12.2) | 75.3$\pm$ (13.4) |

**Table.** Two-way ANOVA analysis for HDL Cholesterol variable by gender and age. HDL cholesterol (>40 mg/dL)

|  | Df | Sum Sq | Mean Sq | F value | Pr(>F) |
| --- | --- | --- | --- | --- | --- |
| Age | 3 | 884.9525 | 294.9842 | 1.6297064 | 0.1829371 |
| Gender | 1 | 8753.9921 | 8753.9921 | 48.3633997 | 0.0000000 |
| Age:Gender | 3 | 521.7760 | 173.9253 | 0.9608897 | 0.4117676 |
| Residuals | 255 | 46156.1428 | 181.0045 |  |  |

#### Analysis of variable Non-HDL Cholesterol


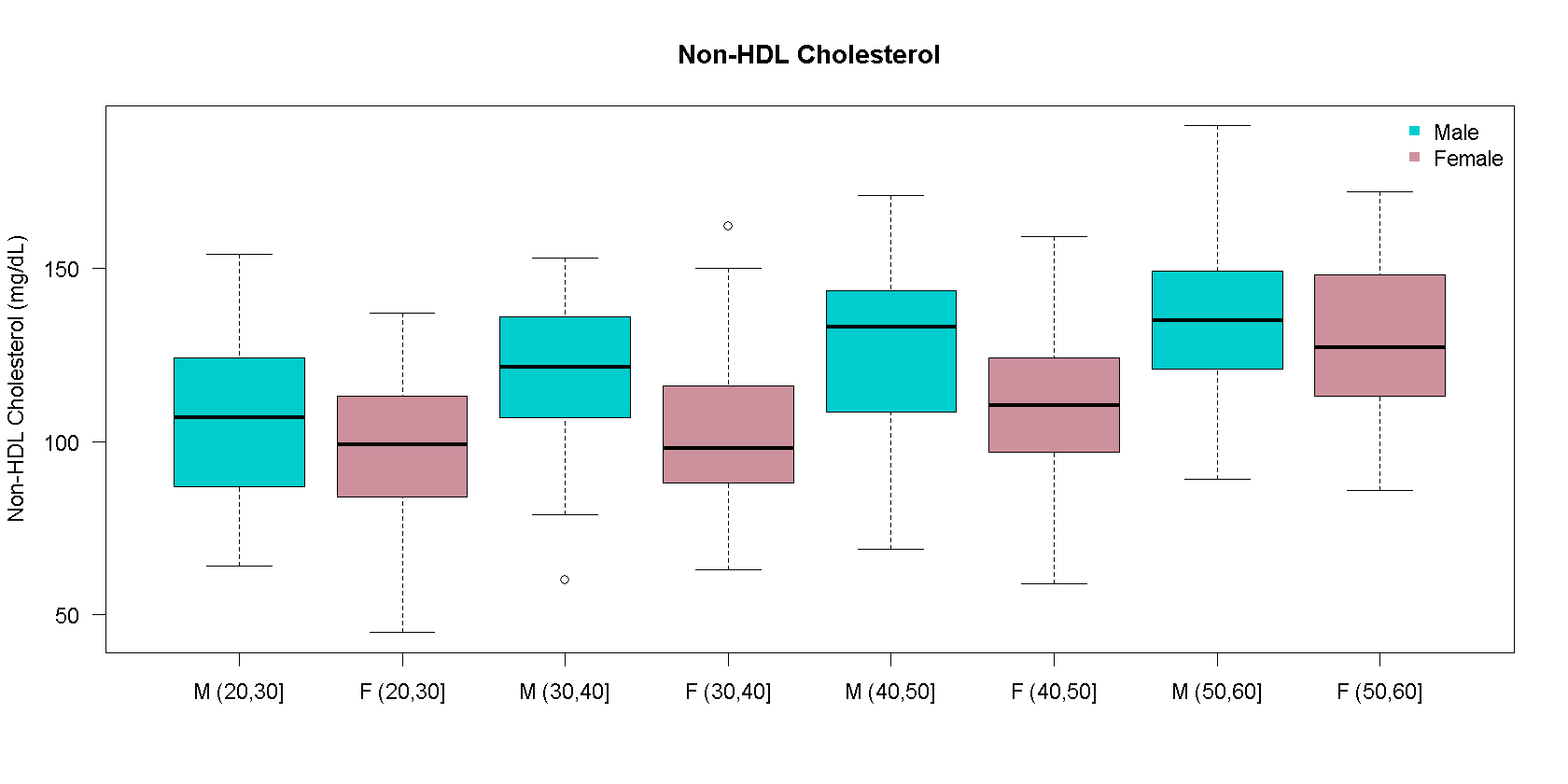


**Figure. Non-HDL Cholesterol**. Boxplot

**Table.** Mean value and standard deviation of Non-HDL Cholesterol variable. Non-HDL Cholesterol (<160 mg/dL)

|  | Male | Female |
| --- | --- | --- |
| (20,30] | 106$\pm$ (24.1) | 97.1$\pm$ (22.1) |
| (30,40] | 120$\pm$ (21.2) | 102$\pm$ (22) |
| (40,50] | 127$\pm$ (24.5) | 110$\pm$ (20.6) |
| (50,60] | 136$\pm$ (22.3) | 131$\pm$ (23.9) |

**Table.** Two-way ANOVA analysis for Non-HDL Cholesterol variable by gender and age. Non-HDL Cholesterol (<160 mg/dL)

|  | Df | Sum Sq | Mean Sq | F value | Pr(>F) |
| --- | --- | --- | --- | --- | --- |
| Age | 3 | 30782.730 | 10260.9100 | 20.110309 | 0.0000000 |
| Gender | 1 | 11309.653 | 11309.6532 | 22.165736 | 0.0000041 |
| Age:Gender | 3 | 1665.313 | 555.1044 | 1.087946 | 0.3547376 |
| Residuals | 255 | 130108.992 | 510.2313 |  |  |

#### Analysis of variable LDL Cholesterol


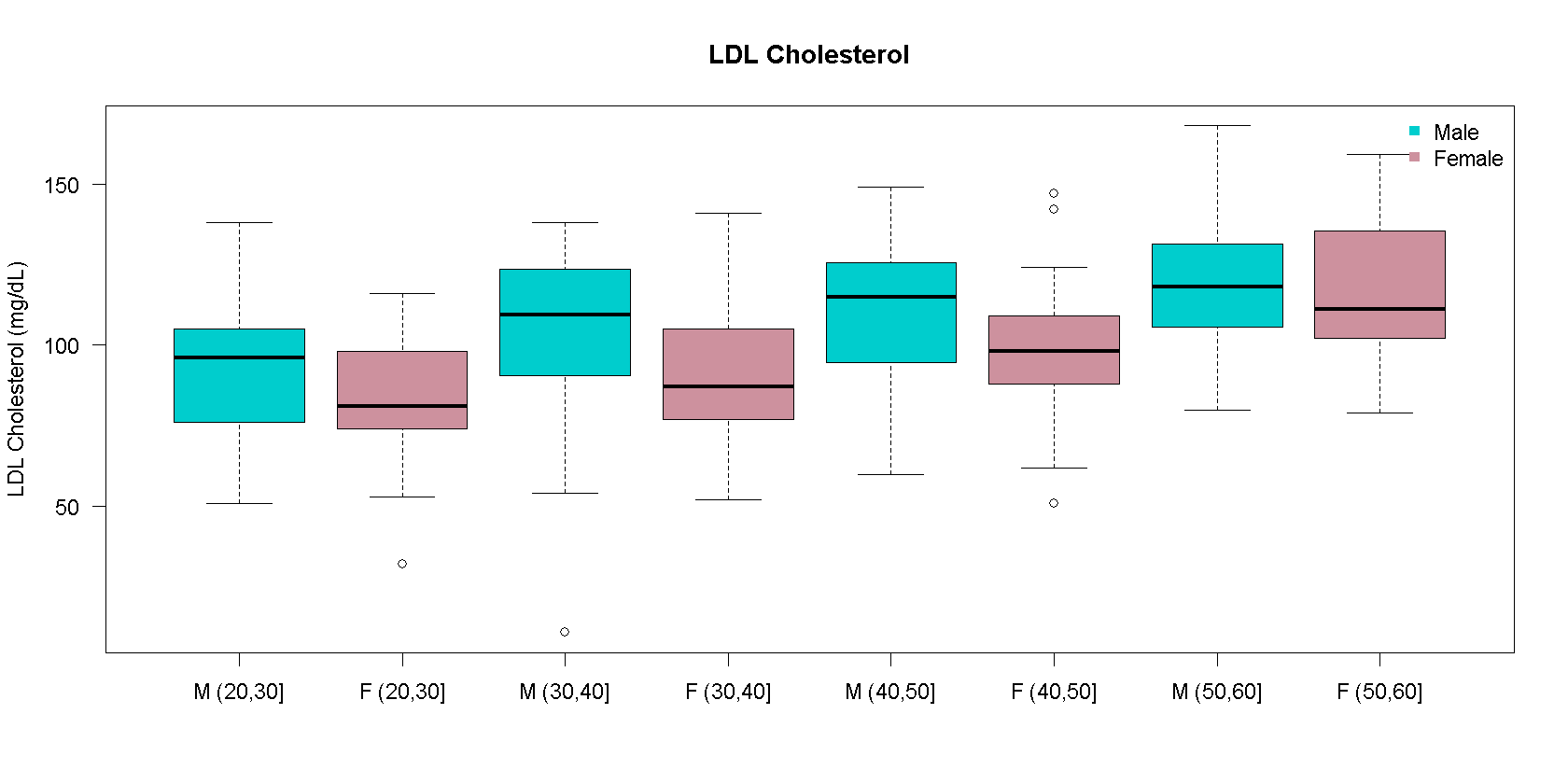


**Figure. LDL Cholesterol**. Boxplot

**Table.** Mean value and standard deviation of LDL Cholesterol variable. LDL Cholesterol (<130 mg/dL)

|  | Male | Female |
| --- | --- | --- |
| (20,30] | 92.8$\pm$ (23) | 83.2$\pm$ (20.6) |
| (30,40] | 103$\pm$ (26.2) | 90.2$\pm$ (20.4) |
| (40,50] | 111$\pm$ (21.2) | 98.3$\pm$ (19.6) |
| (50,60] | 120$\pm$ (19.5) | 118$\pm$ (23.2) |

**Table.** Two-way ANOVA analysis for LDL Cholesterol variable by gender and age. LDL Cholesterol (<130 mg/dL)

|  | Df | Sum Sq | Mean Sq | F value | Pr(>F) |
| --- | --- | --- | --- | --- | --- |
| Age | 3 | 28372.657 | 9457.5522 | 19.9015861 | 0.0000000 |
| Gender | 1 | 6115.974 | 6115.9739 | 12.8698820 | 0.0004002 |
| Age:Gender | 3 | 1312.877 | 437.6256 | 0.9208982 | 0.4312603 |
| Residuals | 255 | 121180.081 | 475.2160 |  |  |

#### Analysis of variable Triglycerides


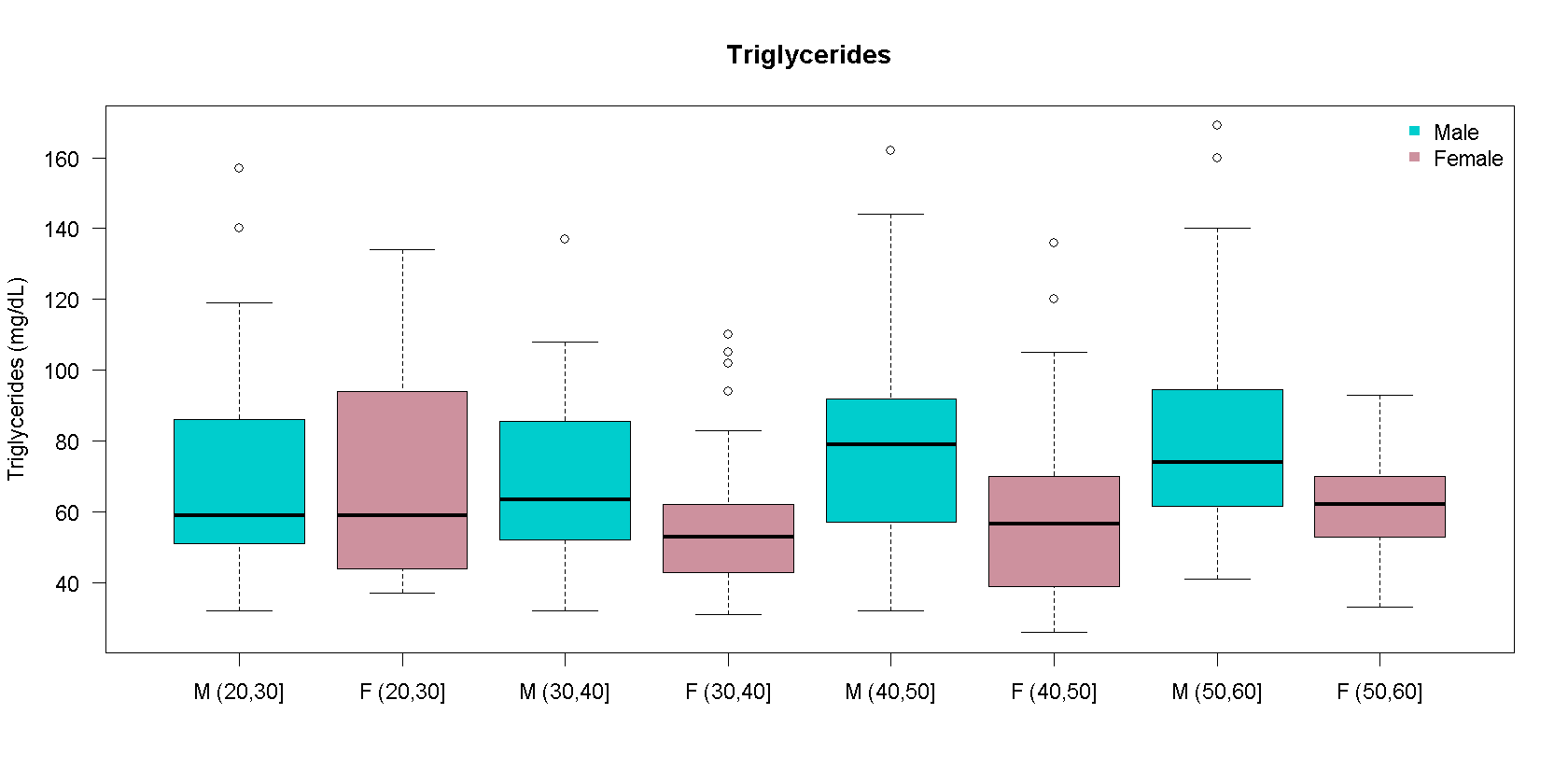


**Figure. Triglycerides**. Boxplot

**Table.** Mean value and standard deviation of Triglycerides variable. Triglycerides (<150 mg/dL)

|  | Male | Female |
| --- | --- | --- |
| (20,30] | 67.6$\pm$ (28.7) | 69.9$\pm$ (30.7) |
| (30,40] | 69.7$\pm$ (23.8) | 57.5$\pm$ (20.9) |
| (40,50] | 79.2$\pm$ (29.4) | 59.7$\pm$ (24.6) |
| (50,60] | 82.8$\pm$ (32) | 61.3$\pm$ (14.1) |

**Table.** Two-way ANOVA analysis for Triglycerides variable by gender and age. Triglycerides (<150 mg/dL)

|  | Df | Sum Sq | Mean Sq | F value | Pr(>F) |
| --- | --- | --- | --- | --- | --- |
| Age | 3 | 3826.413 | 1275.4710 | 1.851945 | 0.1382256 |
| Gender | 1 | 11870.972 | 11870.9718 | 17.236290 | 0.0000450 |
| Age:Gender | 3 | 4639.536 | 1546.5119 | 2.245488 | 0.0834876 |
| Residuals | 255 | 175623.513 | 688.7197 |  |  |

#### Analysis of variable Glucose


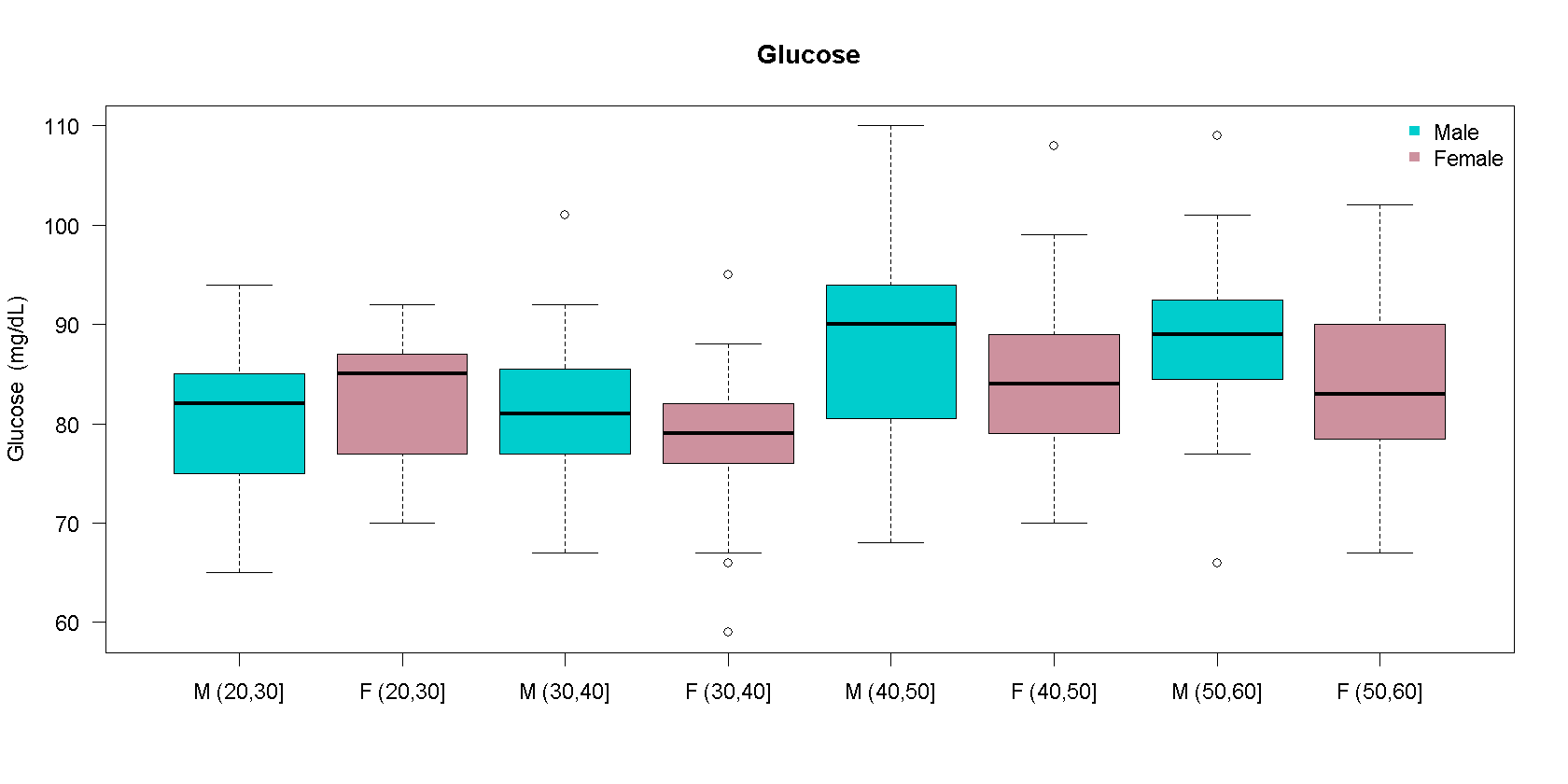


**Figure. Glucose**. Boxplot

**Table.** Mean value and standard deviation of Glucose variable. Glucose (50-110 mg/dL)

|  | Male | Female |
| --- | --- | --- |
| (20,30] | 80.7$\pm$ (7.55) | 83$\pm$ (6.19) |
| (30,40] | 81$\pm$ (6.99) | 79.2$\pm$ (6.95) |
| (40,50] | 87.8$\pm$ (9.08) | 84.2$\pm$ (8.12) |
| (50,60] | 89.2$\pm$ (7.94) | 84.7$\pm$ (8.55) |

**Table.** Two-way ANOVA analysis for Glucose variable by gender and age. Glucose (50-110 mg/dL)

|  | Df | Sum Sq | Mean Sq | F value | Pr(>F) |
| --- | --- | --- | --- | --- | --- |
| Age | 3 | 2472.9925 | 824.33082 | 13.582137 | 0.0000000 |
| Gender | 1 | 296.1921 | 296.19209 | 4.880227 | 0.0280554 |
| Age:Gender | 3 | 358.9486 | 119.64952 | 1.971413 | 0.1187208 |
| Residuals | 255 | 15476.5309 | 60.69228 |  |  |

#### Analysis of variable Urate


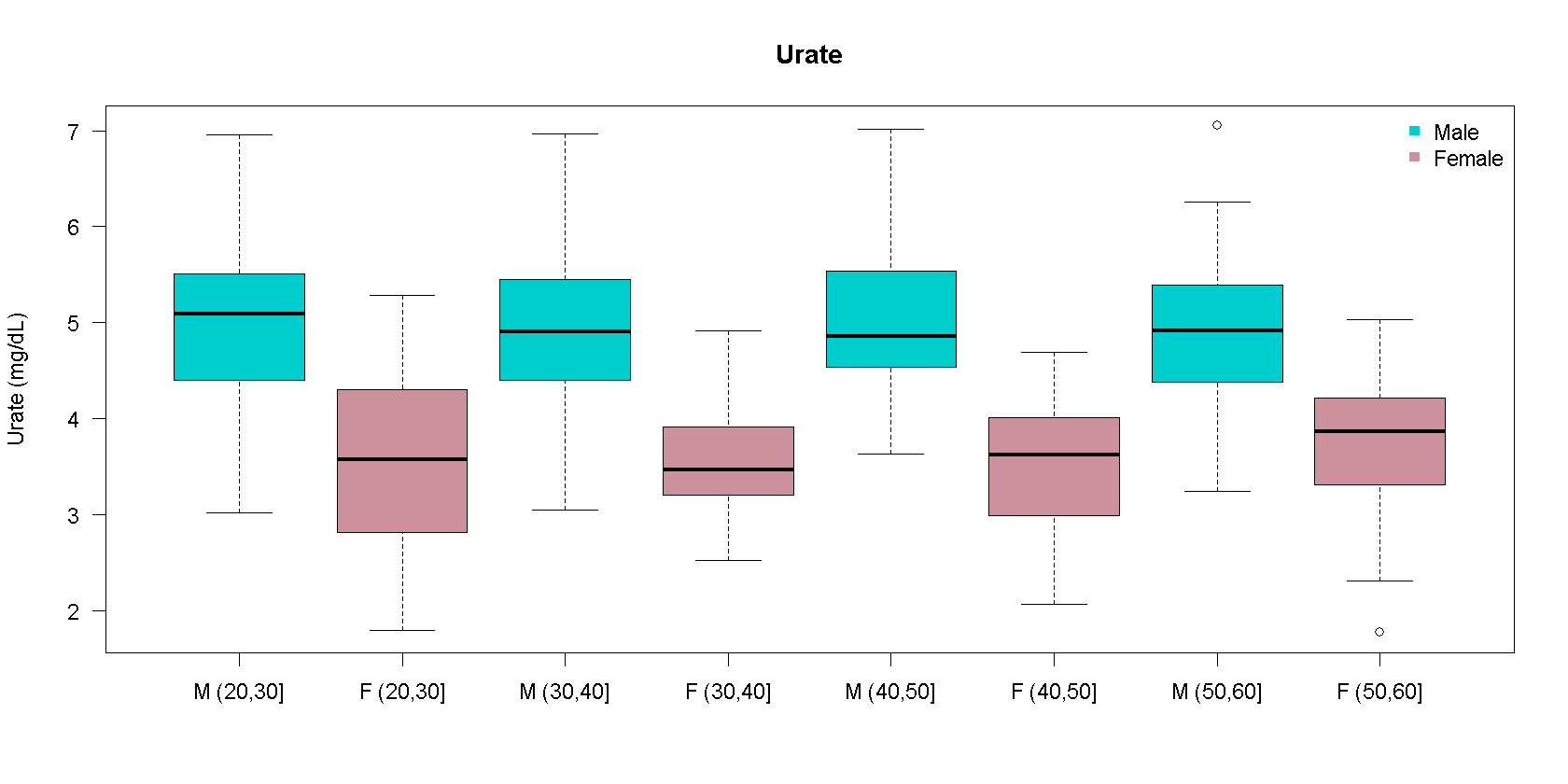


**Figure. Urate**. Boxplot

**Table.** Mean value and standard deviation of Urate variable. Urate Female (2.4-5.7 mg/dL) Male (3.4-7 mg/dL)

|  | Male | Female |
| --- | --- | --- |
| (20,30] | 5.02$\pm$ (0.914) | 3.6$\pm$ (0.928) |
| (30,40] | 4.94$\pm$ (0.877) | 3.57$\pm$ (0.54) |
| (40,50] | 5.05$\pm$ (0.764) | 3.53$\pm$ (0.652) |
| (50,60] | 4.91$\pm$ (0.838) | 3.76$\pm$ (0.786) |

**Table.** Two-way ANOVA analysis for Urate variable by gender and age. Urate Female (2.4-5.7 mg/dL) Male (3.4-7 mg/dL)

|  | Df | Sum Sq | Mean Sq | F value | Pr(>F) |
| --- | --- | --- | --- | --- | --- |
| Age | 3 | 3.797831 | 1.2659436 | 2.0504429 | 0.1073042 |
| Gender | 1 | 120.634255 | 120.6342553 | 195.3907272 | 0.0000000 |
| Age:Gender | 3 | 1.139817 | 0.3799390 | 0.6153854 | 0.6056002 |
| Residuals | 255 | 157.437027 | 0.6174001 |  |  |

#### Analysis of variable Creatinine


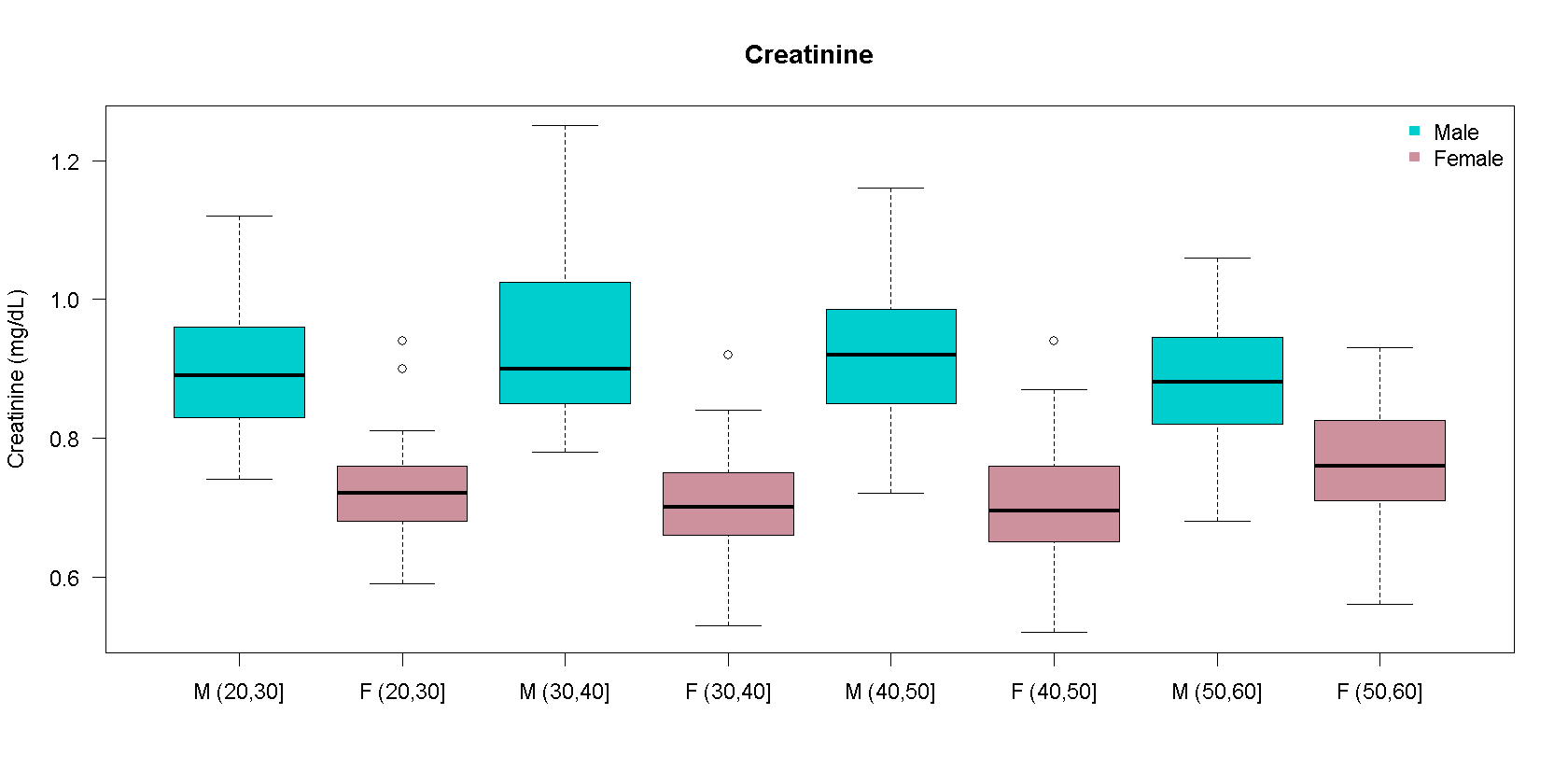


**Figure. Creatinine**. Boxplot

**Table.** Mean value and standard deviation of Creatinine variable. Creatinine Female (0.4-1.1 mg/dL) Male (0.4-1.3 mg/dL)

|  | Male | Female |
| --- | --- | --- |
| (20,30] | 0.904$\pm$ (0.0938) | 0.728$\pm$ (0.0885) |
| (30,40] | 0.943$\pm$ (0.113) | 0.701$\pm$ (0.0809) |
| (40,50] | 0.916$\pm$ (0.104) | 0.707$\pm$ (0.0933) |
| (50,60] | 0.882$\pm$ (0.0901) | 0.752$\pm$ (0.101) |

**Table.** Two-way ANOVA analysis for Creatinine variable by gender and age. Creatinine Female (0.4-1.1 mg/dL) Male (0.4-1.3 mg/dL)

|  | Df | Sum Sq | Mean Sq | F value | Pr(>F) |
| --- | --- | --- | --- | --- | --- |
| Age | 3 | 0.0390008 | 0.0130003 | 1.401229 | 0.2429175 |
| Gender | 1 | 2.4062648 | 2.4062648 | 259.358220 | 0.0000000 |
| Age:Gender | 3 | 0.1076870 | 0.0358957 | 3.869001 | 0.0098720 |
| Residuals | 255 | 2.3658302 | 0.0092778 |  |  |

#### Analysis of variable ALT/GPT


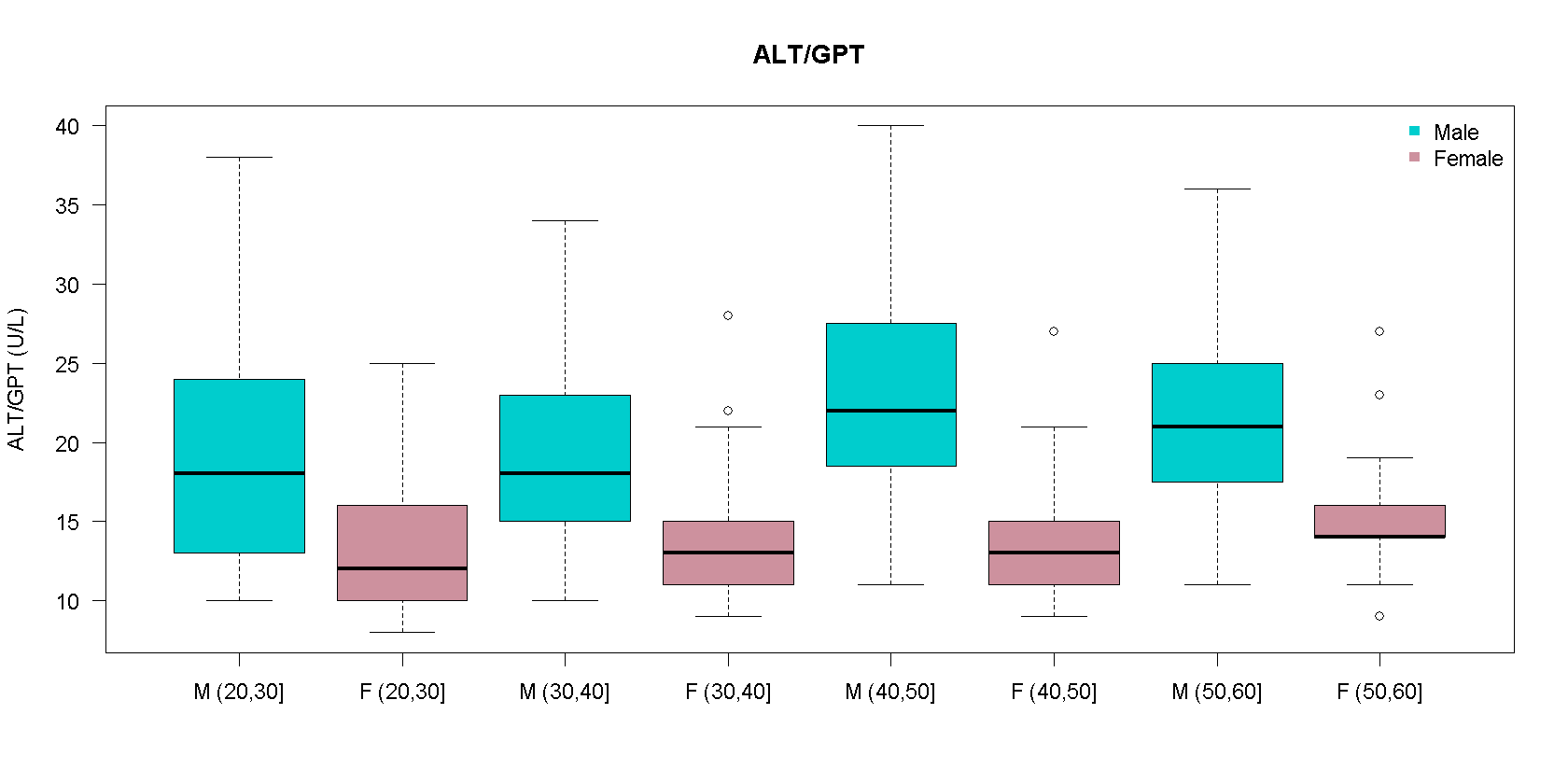


**Figure. ALT/GPT**. Boxplot

**Table.** Mean value and standard deviation of ALT/GPT variable. ALT/GPT Female (0-41 U/L) Male (0-31 U/L)

|  | Male | Female |
| --- | --- | --- |
| (20,30] | 19.3$\pm$ (6.99) | 13.2$\pm$ (4.53) |
| (30,40] | 19.6$\pm$ (5.91) | 13.9$\pm$ (4.32) |
| (40,50] | 23.1$\pm$ (7.71) | 14$\pm$ (4.27) |
| (50,60] | 22.1$\pm$ (6.8) | 15.7$\pm$ (4.12) |

**Table.** Two-way ANOVA analysis for ALT/GPT variable by gender and age. ALT/GPT Female (0-41 U/L) Male (0-31 U/L)

|  | Df | Sum Sq | Mean Sq | F value | Pr(>F) |
| --- | --- | --- | --- | --- | --- |
| Age | 3 | 320.5598 | 106.85326 | 3.069369 | 0.0284596 |
| Gender | 1 | 3105.2929 | 3105.29290 | 89.199814 | 0.0000000 |
| Age:Gender | 3 | 128.9847 | 42.99490 | 1.235032 | 0.2974724 |
| Residuals | 255 | 8877.2572 | 34.81277 |  |  |

#### Analysis of variable Gamma GT (GGT)


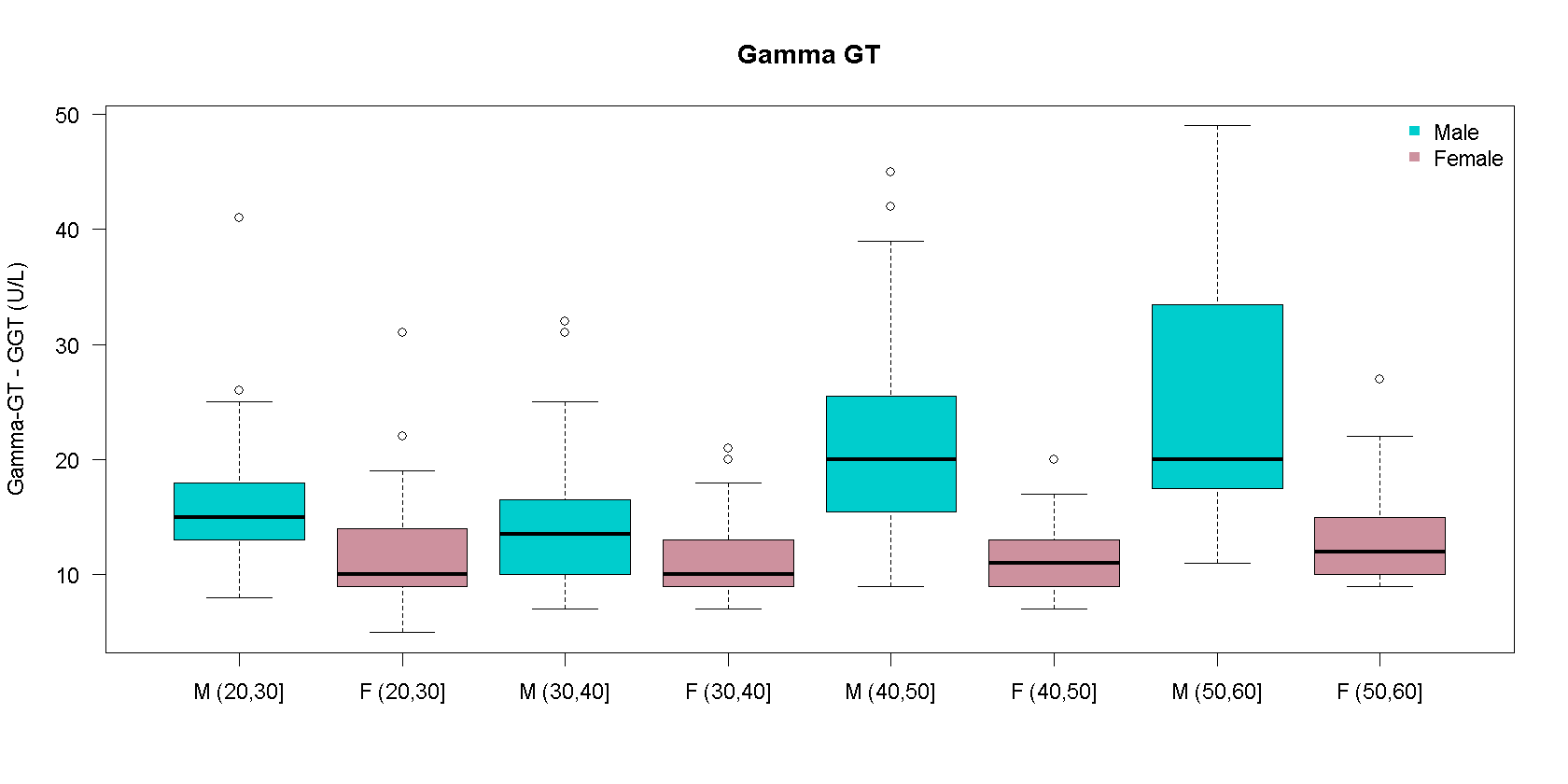


**Figure. Gamma GT - GGT**. Boxplot

**Table.** Mean value and standard deviation of Gamma GT variable. Gamma-GT - GGT Female (<40 U/L) Male (<60 U/L)

|  | Male | Female |
| --- | --- | --- |
| (20,30] | 16.7$\pm$ (6.04) | 12.3$\pm$ (6.34) |
| (30,40] | 14.6$\pm$ (5.79) | 11.4$\pm$ (3.68) |
| (40,50] | 21.5$\pm$ (8.52) | 11.5$\pm$ (2.99) |
| (50,60] | 24.4$\pm$ (10.4) | 13.6$\pm$ (4.74) |

**Table.** Two-way ANOVA analysis for GGT variable by gender and age. GGT - GGT Female (<40 U/L) Male (<60 U/L)

|  | Df | Sum Sq | Mean Sq | F value | Pr(>F) |
| --- | --- | --- | --- | --- | --- |
| Age | 3 | 1699.627 | 566.54229 | 13.25398 | 0.0000000 |
| Gender | 1 | 3250.860 | 3250.86014 | 76.05229 | 0.0000000 |
| Age:Gender | 3 | 715.773 | 238.59099 | 5.58172 | 0.0010078 |
| Residuals | 255 | 10899.992 | 42.74507 |  |  |
