# Supplementary material for: Data in support of enhancing metabolomics research through data mining
Source: Data Brief. 2015 Feb 27;3:155–64. doi: 10.1016/j.dib.2015.02.008 (PMC4510074; doi:10.1016/j.dib.2015.02.008)
Supplement: Supplementary file 2 — Supplementary Material [file mmc2.doc]

### Supplementary Material 2

### Univariate normality. Box-Cox transformations

For some statistical analysis, like MANOVA, it's necessary that some requirements or hypothesis are met.

The one-parameter Box-Cox transformations are defined as:

$${y_{i}}^{(\lambda)}=\left\{ \begin{matrix} \frac{{y_{i}}^{\lambda}y_{i}^{\lambda}-1}{\lambda\lambda};\mathrm{if}\lambda\lambda\neq0, \\ \ln(y_{i});\mathrm{if}\lambda\lambda=0, \end{matrix} \right.$$

The most common Box-Cox transformations are described in the following table.

| ***λ*** | **Y’** |
| --- | --- |
| *λ*=−2 | Y’ = 1/ Y^2^ |
| *λ*=−1 | Y’ = 1 / Y |
| *λ*=−0.5 | Y’ = 1 / sqrt(Y) |
| *λ*=0 | Y’ = log(Y) |
| *λ*=0.5 | Y’ = sqrt(Y) |
| *λ*=1 | Y’ = Y |
| *λ*=2 | Y’ = Y^2^ |

Common Box-Cox Transformations $\lambda\lambda=-2, -1,-0.5, 0, 0.5, 1, 2$.

Function tboxcox allows to determine the optimal value for $\lambda\lambda$.

tboxcox <- function(X = X, lambda = seq(-5, 5, 0.1), plot.it = FALSE) {
 X <- X[!is.na(X)]

 if (length(X)>=3) {
 CORXY <- c()
 for (i in lambda) {
 if (i == 0) {
 Y <- log(X)
 } else {
 Y <- (X^i - 1) / i
 }

 XN <- qqnorm(Y, plot.it = FALSE)
 CORXY <- c(CORXY, cor.test(XN$x,XN$y)$estimate)
 }

 bestlambda <- lambda[which.max(CORXY)]
 } else {
 bestlambda <- numeric(0)
 CORXY <- rep(0, length(lambda))
 }

 if (length(bestlambda)==0) {
 bestlambda <- 1
 correlacion <- format(max(0, na.rm = TRUE),digits = 3)
 } else {
 correlacion <- format(max(CORXY, na.rm = TRUE), digits = 3)
 }

 if (bestlambda == 0) {
 Y <- log(X)
 } else {
 Y <- (X^bestlambda - 1) / bestlambda
 }


 if (plot.it) {

 hist(X, main = "Original Data", col = "red", xlab = "",
 cex.axis = 0.7, yaxt = "n")
 axis(2, las = 2, cex.axis = 0.7)

 plot(lambda,CORXY, type = "l", main = "Box-Cox Normality Plot Y", lwd = 2,
 ylim = c(0,1), xlim = c(min(lambda),max(lambda)),
 xlab = substitute(
 paste(lambda, ": ", bl, " ",Correlation,": ",mc,sep=""),
 list(bl = bestlambda, mc = correlacion)),
 ylab = "Correlation Coefficient",
 cex.axis = 0.7, yaxt = "n")
 axis(2, las = 2, cex.axis = 0.7)
 abline(v = bestlambda)


 hist(Y, main = "Transformed Data",
 col = "green4",
 xlab = "",
 cex.axis = 0.7,
 yaxt = "n")
 axis(2, las = 2, cex.axis = 0.7)

 qqx <- qqnorm(X, plot.it = FALSE)
 qqy <- qqnorm(Y, plot.it = FALSE)

 qqnorm(Y, cex.axis = 0.7,
 xlim = range(c(qqx$x,qqy$x)),
 ylim = range(c(qqx$y,qqy$y)),
 main = "Normal Q-Q Plot",
 pch = 16,
 col = "green4",
 cex = 1.5, yaxt = "n")
 axis(2, las = 2, cex.axis = 0.7)
 qqline(Y, col = "green4")
 points(qqx$x, qqx$y, pch = 16, col = "red", cex = 1.5)
 qqline(X, col = "red")

 legend(x = "topleft",
 legend = c("Original Data", "Transformed Data"),
 bty = "n",
 pch = c(16,16),
 col = c("red","green4"),
 cex = 0.8)
 }
 return(list(X = X,
 Y = Y,
 bestlambda = bestlambda,
 lambda = lambda,
 cor = CORXY))
}
