# Supplementary material for: Data in support of enhancing metabolomics research through data mining
Source: Data Brief. 2015 Feb 27;3:155–64. doi: 10.1016/j.dib.2015.02.008 (PMC4510074; doi:10.1016/j.dib.2015.02.008)
Supplement: Supplementary file 3 — Supplementary Material [file mmc3.doc]

### Supplementary Material 3

How tboxcox works can be tested by generating values of a normal distribution and applying the inverse transformations on them.

set.seed(1234)
X <- rnorm(5000, mean = 10, sd = 2)

#### Example: Transformation 1 / X^2^. The optimal parameter is lambda = -0.5

XX <- X^(-2)
par(mfrow = c(2, 2))
AUX <- tboxcox(XX, lambda = seq(-2, 2, by = 0.5), plot.it = TRUE)


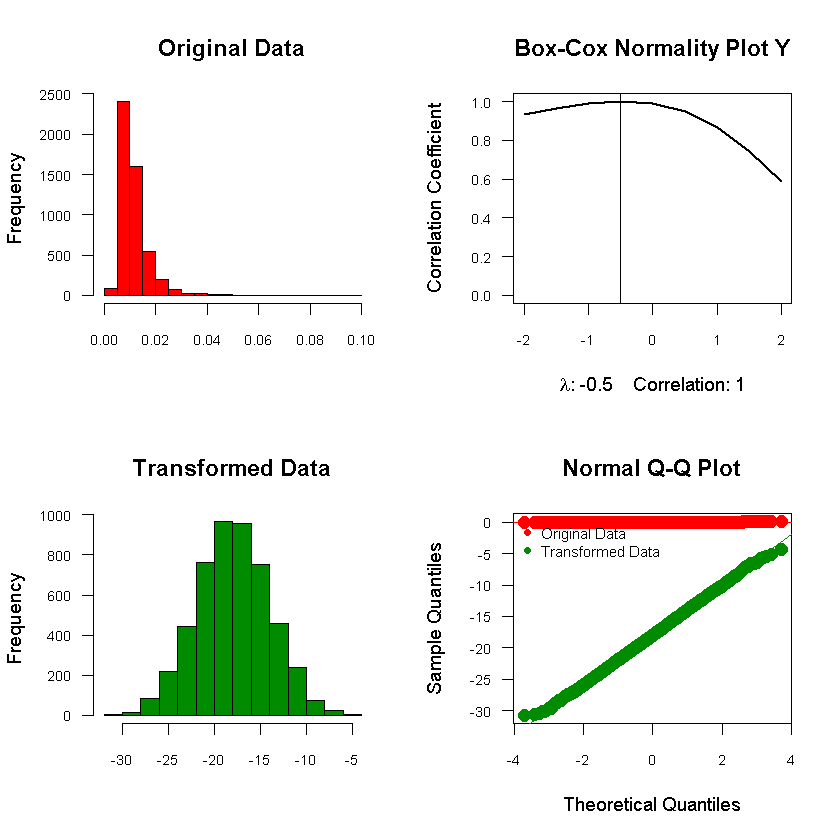


**Figure**: XX = 1 / X^2^. The optimal parameter is lambda = -0.5

#### Example: Transformation 1 / X. The optimal parameter is lambda = -1.0

XX <- X^(-1)
par(mfrow = c(2, 2))
AUX <- tboxcox(XX, lambda = seq(-2, 2, by = 0.5), plot.it = TRUE)


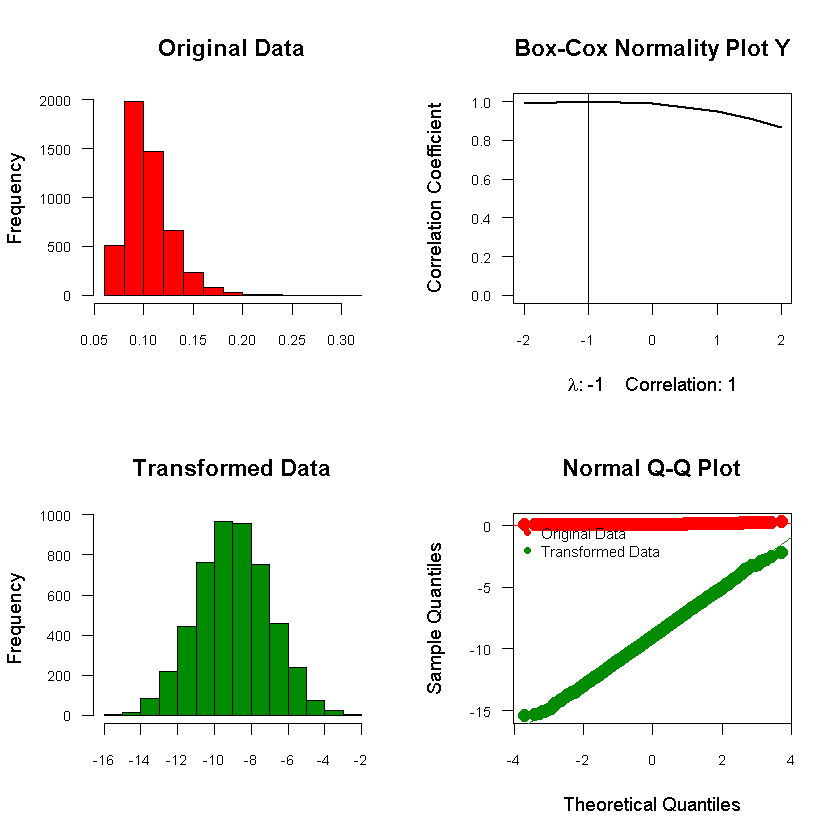


$XX$**Figure**: XX= 1 / X. The optimal parameter is lambda = -1.0

#### Example: Transformation 1 / sqrt(X). The optimal parameter is lambda = -2.0

XX <- X^(-0.5)
par(mfrow = c(2, 2))
AUX <- tboxcox(XX, lambda = seq(-2, 2, by = 0.5), plot.it = TRUE)


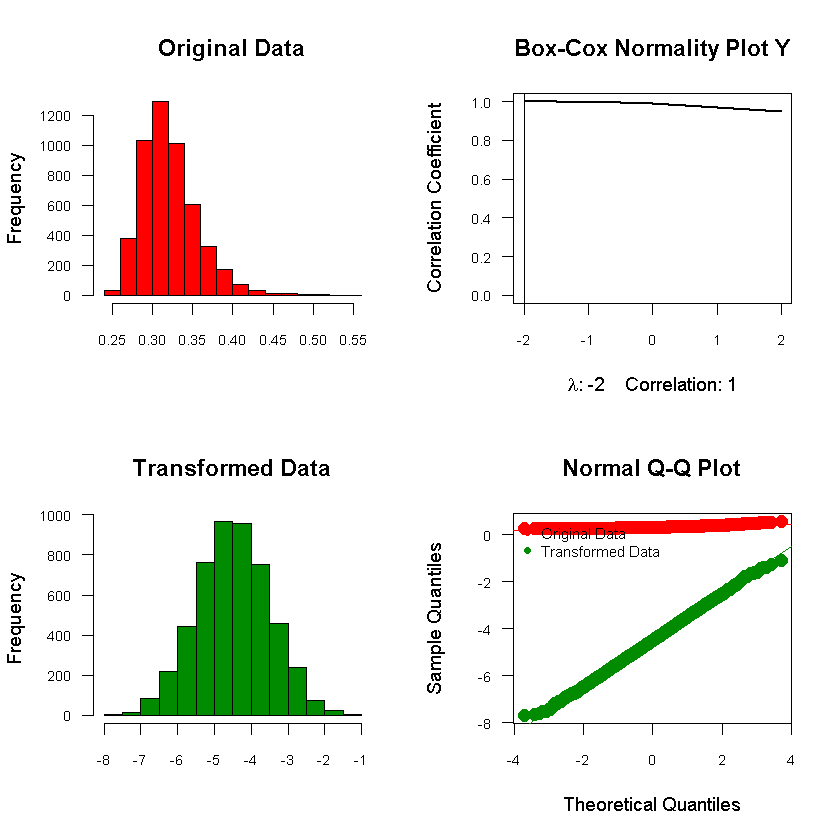


**Figure**: XX = 1 / sqrt(X). The optimal parameter is lambda = -2.0

#### Example: Transformation exp(X). The optimal parameter is lambda = 0.0

XX <- exp(X)
par(mfrow = c(2, 2))
AUX <- tboxcox(XX, lambda = seq(-2, 2, by = 0.5), plot.it = TRUE)


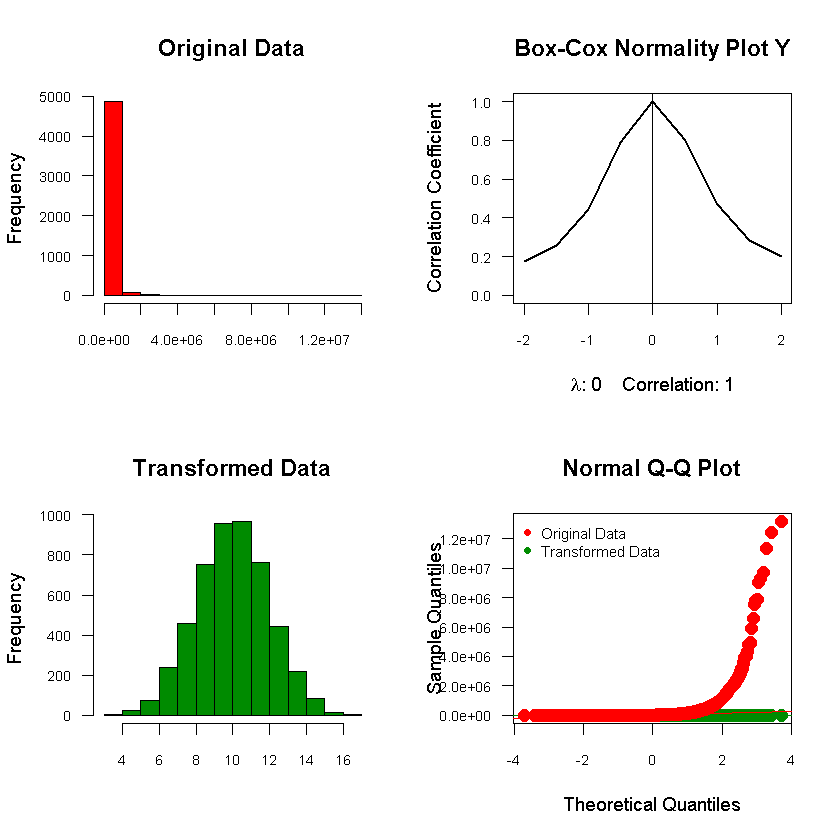


**Figure**: XX = exp(X). The optimal parameter is lambda = 0.0

#### Example: Transformation sqrt(X). The optimal parameter is lambda = 2.0

XX <- X^(0.5)
par(mfrow = c(2, 2))
AUX <- tboxcox(XX, lambda = seq(-2, 2, by = 0.5), plot.it = TRUE)


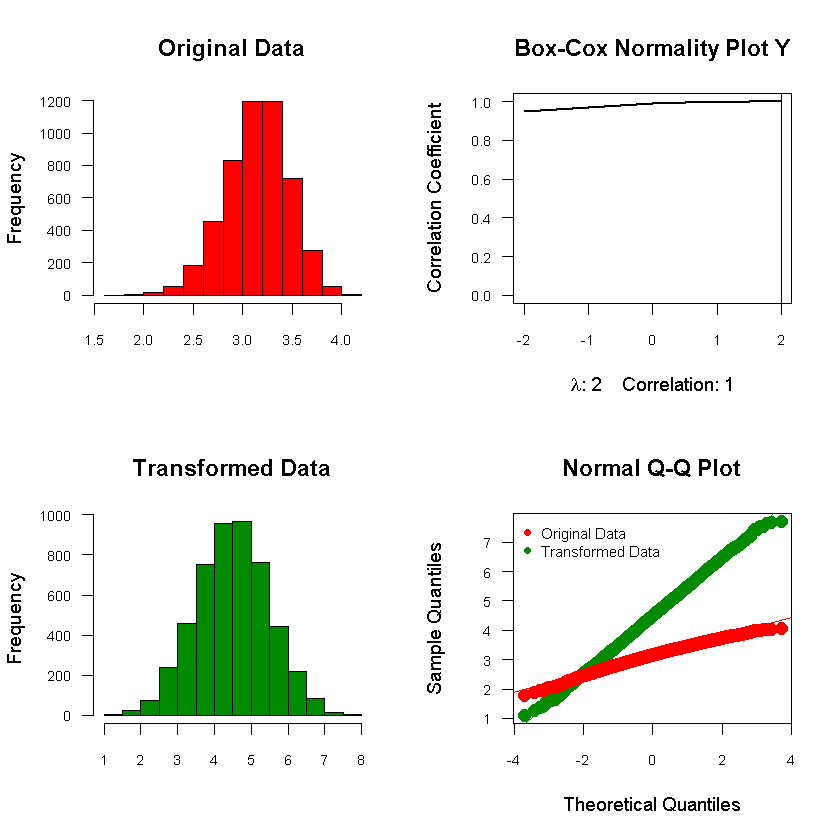


**Figure**: XX = sqrt(X). The optimal parameter is lambda = 2.0
