# Supplementary material for: Data in support of enhancing metabolomics research through data mining
Source: Data Brief. 2015 Feb 27;3:155–64. doi: 10.1016/j.dib.2015.02.008 (PMC4510074; doi:10.1016/j.dib.2015.02.008)
Supplement: Supplementary file 4 — Supplementary Material [file mmc4.doc]

## Supplementary Material 4

## levene_test()

levene_test <- function(x, g,
 alpha = 0.05,
 plot = TRUE,
 varname = "Y",
 border = "black",
 ylab = "",
 xlab = "",
 lwd = 2,
 cex.axis = 1.2) {

 k <- nlevels(g)
 N <- length(x)
 Ni <- table(g)
 med <- tapply(x, g, "median")
 Zij <- abs(x - med[g])
 Zi <- tapply(Zij, g, "mean")

 W.num <- (N - k) * sum(Ni * (Zi - mean(Zij))^2)
 W.den <- (k - 1) * sum((Zij - Zi[g])^2)
 W <- W.num / W.den

 cat(sprintf("\nLEVENE TEST OF HOMOGENEITY OF VARIANCES"))
 cat(sprintf("\nThe Levene test statistic is designed to test for equality of variances across groups against the alternative that variances are unequal for at least two groups.\n"))
 cat(sprintf("\n\tH0:\tsigma_{1}^{2} = sigma_{2}^{2} = ... = sigma_{k}^{2}"))
 cat(sprintf("\n\tHa:\tsigma_{i}^{2} != sigma_{j}^{2} for at least one pair (i,j)"))
 cat(sprintf("\n\nTest statistic: W = %.5f", W))
 cat(sprintf("\nDegrees of freedom (df): k - 1 = %i", k-1))
 cat(sprintf("\nSignificance level: alpha = %.3f", alpha))
 cat(sprintf("\nCritical value: %.5f", qf(alpha, k-1, N-k, lower.tail = FALSE)))
 cat(sprintf("\nCritical region: Reject H0 if W > %.5f", qf(alpha, k-1, N-k, lower.tail = FALSE)))
 cat(sprintf("\np-value: %e", pf(W, k-1, N-k, lower.tail = FALSE)))
 cat(sprintf("\n\n\n"))

 if (plot) {

 V <- vector("list", k)
 for (i in 1:k) {
 V[[i]] <- x[g == levels(g)[i]]
 }

 title <- vector("list", 3)
 title[[1]] <- varname
 title[[2]] <- paste(varname, " - median(", varname, ")", sep = "")
 title[[3]] <- paste("abs(", varname, " - median(", varname, "))", sep = "")

 par(mfrow = c(1,3))

 boxplot(V, main = title[[1]], xaxt = "n", yaxt = "n",
 cex.main = 1.5,
 col = rainbow(k),
 ylab = ylab,
 cex.lab = 1.5,
 border = border,
 lwd = lwd)
 axis(1, at = 1:k, label = levels(g), cex.axis = cex.axis)
 axis(2, las = 2, cex.axis = cex.axis)

 med <- tapply(x, g, FUN = "median")
 for (i in 1:k) {
 V[[i]] <- V[[i]] - med[i]
 }
 boxplot(V, main = title[[2]], xaxt = "n", yaxt = "n",
 cex.main = 1.5,
 col = rainbow(k),
 xlab = xlab,
 cex.lab = 1.5,
 border = border,
 lwd = lwd)
 axis(1, at = 1:k, label = levels(g), cex.axis = cex.axis)
 axis(2, las = 2, cex.axis = cex.axis)

 boxplot(lapply(V, "abs"), main = title[[3]], xaxt = "n", yaxt = "n",
 cex.main = 1.5,
 col = rainbow(k),
 border = border,
 lwd = lwd)
 axis(1, at = 1:k, label = levels(g), cex.axis = cex.axis)
 axis(2, las = 2, cex.axis = cex.axis)

 }

 names(W) <- "Bartlett's K-squared"
 df <- k-1
 names(df) <- "df"

 return(structure(list(statistic = W,
 parameter = df,
 p.value = pf(W, k-1, N-k, lower.tail = FALSE),
 method = "Levene Test of Homogeneity of Variances",
 data.name = deparse(substitute(x))),
 class="htest"))

}

## bartlett_test()

bartlett_test <- function(x, g,
 alpha = 0.05,
 plot = TRUE,
 varname = "Y",
 border = "black",
 ylab = "",
 xlab = "",
 lwd = 2,
 cex.axis = 1.2) {

 N <- length(x)
 Ni <- table(g)
 sigmai <- tapply(X = x, INDEX = g, FUN = "var")
 k <- nlevels(g)

 sigmap <- sum((Ni - 1)*sigmai / (N - k))

 bt.T <- (N - k) * log(sigmap) - sum((Ni - 1)*log(sigmai))
 A1 <- 1 / (3*(k - 1))
 A2 <- sum(1 / (Ni - 1)) - 1 /(N - k)
 bt.T <- bt.T / (1 + A1*A2)

 cat(sprintf("\nBARTLETT TEST OF HOMOGENEITY OF VARIANCES"))
 cat(sprintf("\nThe Bartlett test statistic is designed to test for equality of variances across groups against the alternative that variances are unequal for at least two groups.\n"))
 cat(sprintf("\n\tH0:\tsigma_{1}^{2} = sigma_{2}^{2} = ... = sigma_{k}^{2}"))
 cat(sprintf("\n\tHa:\tsigma_{i}^{2} != sigma_{j}^{2} for at least one pair (i,j)"))
 cat(sprintf("\n\nTest statistic: T = %.5f", bt.T))
 cat(sprintf("\nDegrees of freedom (df): k - 1 = %i", k-1))
 cat(sprintf("\nSignificance level: alpha = %.3f", alpha))
 cat(sprintf("\nCritical value: %.5f", qchisq(alpha, k-1, lower.tail = FALSE)))
 cat(sprintf("\nCritical region: Reject H0 if T > %.5f", qchisq(alpha, k-1, lower.tail = FALSE)))
 cat(sprintf("\np-value: %e", pchisq(bt.T, k-1, lower.tail = FALSE)))
 cat(sprintf("\n\n\n"))


 if (plot) {

 V <- vector("list", k)
 for (i in 1:k) {
 V[[i]] <- x[g == levels(g)[i]]
 }

 title <- vector("list", 3)
 title[[1]] <- varname
 title[[2]] <- paste(varname, " - median(", varname, ")", sep = "")
 title[[3]] <- paste("abs(", varname, " - median(", varname, "))", sep = "")

 par(mfrow = c(1,3))

 boxplot(V, main = title[[1]], xaxt = "n", yaxt = "n",
 cex.main = 1.5,
 col = rainbow(k),
 ylab = ylab,
 cex.lab = 1.5,
 border = border,
 lwd = lwd)
 axis(1, at = 1:k, label = levels(g), cex.axis = cex.axis)
 axis(2, las = 2, cex.axis = cex.axis)

 med <- tapply(x, g, FUN = "median")
 for (i in 1:k) {
 V[[i]] <- V[[i]] - med[i]
 }
 boxplot(V, main = title[[2]], xaxt = "n", yaxt = "n",
 cex.main = 1.5,
 col = rainbow(k),
 xlab = xlab,
 cex.lab = 1.5,
 border = border,
 lwd = lwd)
 axis(1, at = 1:k, label = levels(g), cex.axis = cex.axis)
 axis(2, las = 2, cex.axis = cex.axis)

 boxplot(lapply(V, "abs"), main = title[[3]], xaxt = "n", yaxt = "n",
 cex.main = 1.5,
 col = rainbow(k),
 border = border,
 lwd = lwd)
 axis(1, at = 1:k, label = levels(g), cex.axis = cex.axis)
 axis(2, las = 2, cex.axis = cex.axis)

 }

 names(bt.T) <- "Bartlett's K-squared"
 df <- k-1
 names(df) <- "df"

 return(structure(list(statistic = bt.T,
 parameter = df,
 p.value = pchisq(bt.T, k-1, lower.tail = FALSE),
 method = "Bartlett Test of Homogeneity of Variances",
 data.name = deparse(substitute(x))),
 class="htest"))

}
