# Supplementary material for: Data in support of enhancing metabolomics research through data mining
Source: Data Brief. 2015 Feb 27;3:155–64. doi: 10.1016/j.dib.2015.02.008 (PMC4510074; doi:10.1016/j.dib.2015.02.008)

### Supplementary material 5

### Homogeneity of variances. Homoscedasticity

#### Importance of the assumption of homogeneity of variances

**Example: Assumption of homogeneity of variances not accepted**

## A B C
## 2.575978 4.875193 6.235124

## A B C
## 0.8693823 7.1345421 4.1972638

##
## BARTLETT TEST OF HOMOGENEITY OF VARIANCES
## The Bartlett test statistic is designed to test for equality of variances across groups against the alternative that variances are unequal for at least two groups.
##
## H0: sigma_{1}^{2} = sigma_{2}^{2} = ... = sigma_{k}^{2}
## Ha: sigma_{i}^{2} != sigma_{j}^{2} for at least one pair (i,j)
##
## Test statistic: T = 55.59314
## Degrees of freedom (df): k - 1 = 2
## Significance level: alpha = 0.050
## Critical value: 5.99146
## Critical region: Reject H0 if T > 5.99146
## p-value: 8.474289e-13


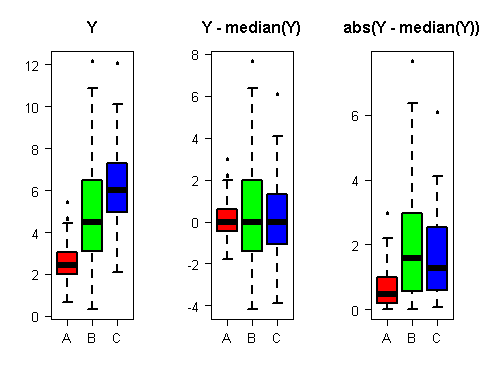


**Example: Assumption of homogeneity of variances accepted**

##
## Bartlett Test of Homogeneity of Variances
##
## data: DATA$B.N
## Bartlett's K-squared = 55.5931, df = 2, p-value = 8.474e-13

## Df Sum Sq Mean Sq F value Pr(>F)
## G 2 410.5 205.25 50.47 <2e-16 ***
## Residuals 177 719.9 4.07
## ---
## Signif. codes: 0 '***' 0.001 '**' 0.01 '*' 0.05 '.' 0.1 ' ' 1

## Tukey multiple comparisons of means
## 95% family-wise confidence level
##
## Fit: aov(formula = B.N ~ G, data = DATA)
##
## $G
## diff lwr upr p adj
## B-A 2.299214 1.4289455 3.169483 0.0000000
## C-A 3.659146 2.7888768 4.529414 0.0000000
## C-B 1.359931 0.4896625 2.230200 0.0008565

Assumption of homogeneity of variances accepted

tapply(DATA$B.Y, INDEX = DATA$G, FUN = "mean")

## A B C
## 3.108556 4.637981 5.989885

tapply(DATA$B.Y, INDEX = DATA$G, FUN = "var")

## A B C
## 1.3542702 0.8026193 0.9260304

bartlett_test(x = DATA$B.Y, g = DATA$G, varname = "Y")

##
## BARTLETT TEST OF HOMOGENEITY OF VARIANCES
## The Bartlett test statistic is designed to test for equality of variances across groups against the alternative that variances are unequal for at least two groups.
##
## H0: sigma_{1}^{2} = sigma_{2}^{2} = ... = sigma_{k}^{2}
## Ha: sigma_{i}^{2} != sigma_{j}^{2} for at least one pair (i,j)
##
## Test statistic: T = 4.40684
## Degrees of freedom (df): k - 1 = 2
## Significance level: alpha = 0.050
## Critical value: 5.99146
## Critical region: Reject H0 if T > 5.99146
## p-value: 1.104246e-01


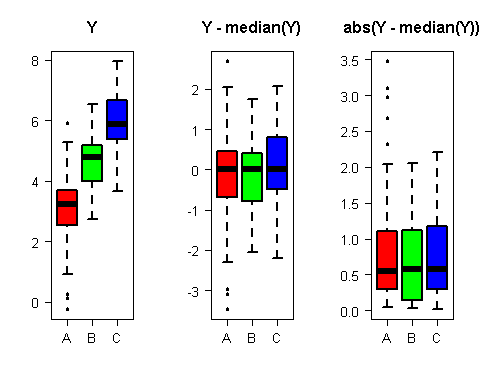

Supplement: Supplementary file 5 — Supplementary Material [file mmc5.doc]
