# Supplementary material for: Data in support of enhancing metabolomics research through data mining
Source: Data Brief. 2015 Feb 27;3:155–64. doi: 10.1016/j.dib.2015.02.008 (PMC4510074; doi:10.1016/j.dib.2015.02.008)
Supplement: Supplementary file 6 — Supplementary Material [file mmc6.doc]

## Supplementary Material 6

## Linear regression

#### Residual analysis

## Min. 1st Qu. Median Mean 3rd Qu. Max.
## -1.8480 -0.2731 -0.0385 0.0000 0.2892 1.2700

## Min. 1st Qu. Median Mean 3rd Qu. Max.
## -1.011000 -0.385800 0.007367 0.012590 0.300500 1.654000

##
## F test to compare two variances
##
## data: AUX.train$Y - yhat.train and AUX.set$Y - yhat.set
## F = 0.6743, num df = 209, denom df = 52, p-value = 0.0565
## alternative hypothesis: true ratio of variances is not equal to 1
## 95 percent confidence interval:
## 0.4249469 1.0108257
## sample estimates:
## ratio of variances
## 0.6743167

##
## Two Sample t-test
##
## data: AUX.train$Y - yhat.train and AUX.set$Y - yhat.set
## t = -0.1769, df = 261, p-value = 0.8597
## alternative hypothesis: true difference in means is not equal to 0
## 95 percent confidence interval:
## -0.1526972 0.1275215
## sample estimates:
## mean of x mean of y
## -2.744069e-16 1.258786e-02

There were no differences between the residuals distribution in estimation and validation sets.


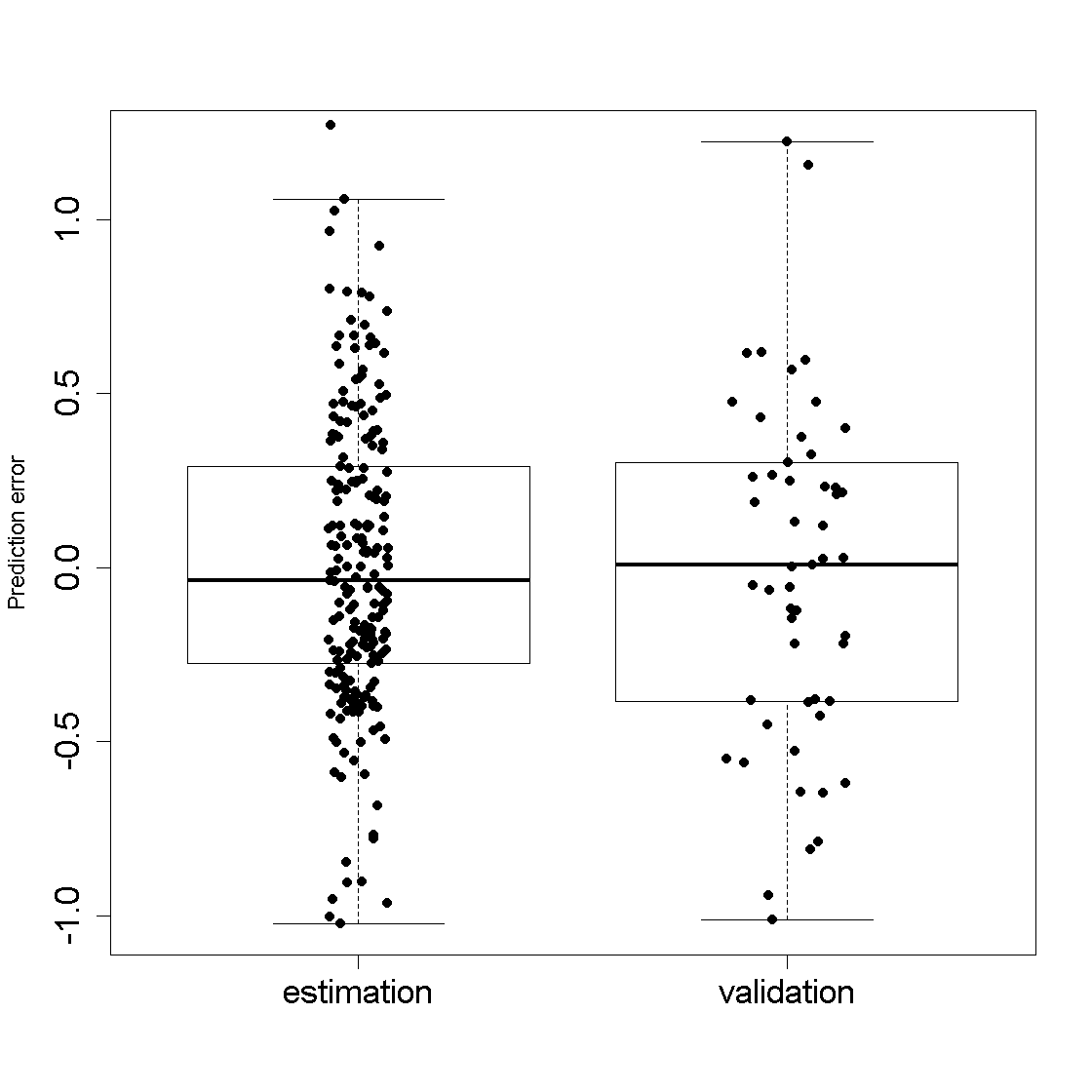


***Figure****: Residual boxplot in estimation and validation sets*
