# Supplementary material for: Data in support of enhancing metabolomics research through data mining
Source: Data Brief. 2015 Feb 27;3:155–64. doi: 10.1016/j.dib.2015.02.008 (PMC4510074; doi:10.1016/j.dib.2015.02.008)
Supplement: Supplementary file 7 — Supplementary Material [file mmc7.doc]

**Supplementary Table 1:** ANOVA analysis. Factors: age. Mean difference is significant at the 0.05 level.

| **Metabolite** | **Age**  **(F value)** | **Age**  **(Pr>F)** |
| --- | --- | --- |
| Glutamic Acid | 2.450195 | 0.063995 |
| Alanine | 6.971482 | 0.000158 |
| Serine | 0.111879 | 0.953109 |
| Proline | 1.171127 | 0.321219 |
| Leucine | 0.583781 | 0.626137 |
| Asparagine | 0.884493 | 0.449644 |
| Aspartic Acid | 1.053398 | 0.369494 |
| Lysine | 3.060765 | 0.028756 |
| Histidine | 0.481346 | 0.695533 |
| Phenylalanine | 0.814995 | 0.486563 |
| Arginine | 2.243859 | 0.083622 |
| Cystine | 8.435971 | 2.28E-05 |
| Gly-DL-Phe | 3.023013 | 0.030222 |
| DL-2-Aminoadipic acid | 1.78335 | 0.150731 |
| Kynurenine | 3.175516 | 0.024716 |
| 1-Methyl-L-histidine | 1.02275 | 0.383057 |
| Cer(d18:1/16:0) | 4.506347 | 0.004217 |
| Cer(d18:1/20:0) | 9.36681 | 6.71E-06 |
| Cer(d18:1/21:0) | 4.699918 | 0.003257 |
| Cer(d18:1/24:0) | 3.985972 | 0.008436 |
| Cer(d18:1/24:1)+Cer(d18:2/24:0) | 8.126981 | 3.42E-05 |
| Cer(d18:1/18:0) | 7.590373 | 6.95E-05 |
| Cer(d18:1/25:0) | 11.9537 | 2.35E-07 |
| CMH(d18:1/16:0) | 3.255826 | 0.022228 |
| CMH(d18:1/24:1) | 4.197257 | 0.006367 |
| CMH(d18:1/23:0) | 6.104754 | 0.0005 |
| Hydroxy cholesterol | 10.09468 | 2.6E-06 |
| ChoE(16:1) | 4.820769 | 0.002772 |
| ChoE(17:0) | 3.729786 | 0.011859 |
| ChoE(18:0) | 3.283395 | 0.021432 |
| ChoE(18:3) | 9.17893 | 8.58E-06 |
| ChoE(22:4) | 0.386678 | 0.762692 |
| ChoE(17:1) | 4.102351 | 0.007225 |
| PC(16:0/18:0) | 5.998631 | 0.000576 |
| PC(38:5) | 6.206614 | 0.000437 |
| PC(18:2/20:4) | 4.644523 | 0.003507 |
| PC(20:0/20:4) | 0.36207 | 0.780448 |
| PC(40:5) | 1.365646 | 0.25372 |
| PC(18:0/22:5) | 11.00045 | 8.02E-07 |
| PC(40:8) | 4.631279 | 0.00357 |
| PE(16:0/20:4) | 1.155215 | 0.327399 |
| PC(37:2) | 1.237812 | 0.296439 |
| PC(17:0/20:3) | 1.067859 | 0.36324 |
| PC(17:0/20:4) | 0.859835 | 0.462474 |
| PI(18:0/18:2) | 1.267413 | 0.286002 |
| PI(18:0/20:3) | 1.333631 | 0.263855 |
| PI(18:0/22:6) | 0.14965 | 0.929853 |
| DG(34:2) | 0.152572 | 0.927975 |
| DG(36:3) | 2.388902 | 0.069301 |
| DG(36:4) | 1.251299 | 0.291642 |
| 18:1n-9 amide | 0.889288 | 0.447183 |
| 20:0 amide | 1.159229 | 0.32583 |
| 21:1n-x amide | 0.682392 | 0.563519 |
| 22:0 amide | 0.338648 | 0.797402 |
| PC(O-16:0/14:0) | 0.74763 | 0.524593 |
| PC(P-16:0/14:0) | 0.305295 | 0.821557 |
| PC(O-34:0) | 0.12645 | 0.944382 |
| PC(O-18:1/18:2) | 0.352325 | 0.787498 |
| PC(O-16:0/20:3) | 2.914394 | 0.034867 |
| PC(P-36:2) | 0.239793 | 0.868545 |
| PC(P-16:0/20:3) | 1.401071 | 0.242924 |
| PC(O-38:4) | 1.250944 | 0.291767 |
| PC(P-18:0/20:4) | 5.710895 | 0.000845 |
| PC(O-18:2/20:4) | 2.653232 | 0.049107 |
| PC(O-18:1/22:4) | 0.289572 | 0.832918 |
| PC(O-40:5) | 0.413366 | 0.743542 |
| PC(O-42:6) | 0.31131 | 0.817205 |
| PC(O-22:0/20:4) | 0.840468 | 0.472759 |
| PE(P-18:0/18:1) | 0.060405 | 0.980519 |
| PE(P-16:0/20:4) | 0.374041 | 0.771801 |
| PE(P-20:0/18:2) | 0.807072 | 0.490921 |
| PE(P-16:0/22:6) | 4.904303 | 0.00248 |
| PC(P-17:0/20:4) | 0.145617 | 0.932429 |
| PE(P-20:0/20:4) | 2.228183 | 0.085332 |
| SM(d18:0/15:0) | 2.711693 | 0.045491 |
| SM(d18:0/16:0) | 10.95983 | 8.45E-07 |
| SM(d18:0/18:0) | 8.1343 | 3.39E-05 |
| SM(d18:2/16:0) | 10.99697 | 8.05E-07 |
| SM(d18:2/20:0) | 3.654269 | 0.013111 |
| SM(d16:1/24:1) | 12.75789 | 8.4E-08 |
| SM(42:1) | 7.496485 | 7.87E-05 |
| SM(d18:1/25:0) | 19.07127 | 3.32E-11 |
| SM(43:1) | 6.013346 | 0.000565 |
| SM(43:2) | 5.610892 | 0.000966 |
| TG(43:0) | 0.810743 | 0.488898 |
| TG(53:0) | 0.267437 | 0.848841 |
| TG(54:3) | 1.782851 | 0.150826 |
| TG(56:0) | 0.193504 | 0.900772 |
| TG(56:5) | 5.97936 | 0.000591 |
| TG(56:8) | 1.28535 | 0.279842 |
| TG(58:1) | 1.189367 | 0.314263 |
| TG(60:2) | 3.200397 | 0.023917 |
| TG(45:1) | 0.084103 | 0.968669 |
| AC(10:0) | 0.854058 | 0.465523 |
| AC(18:2n-6) | 2.873181 | 0.036808 |
| AC(10:1n-x) | 0.962786 | 0.410836 |
| AC(14:2n-x) | 4.374641 | 0.005026 |
| Taurochenodeoxycholic acid | 6.778075 | 0.000204 |
| Glycocholic acid | 4.82652 | 0.002751 |
| Glycochenodeoxycholic acid | 7.420787 | 8.7E-05 |
| 16:1n-7 | 3.463739 | 0.01688 |
| 16:1n-x | 1.680659 | 0.171568 |
| 18:0 | 2.802537 | 0.040385 |
| 20:1n-9 | 1.335052 | 0.263397 |
| 20:4n-6 | 0.93605 | 0.423761 |
| 20:4n-3 | 0.780709 | 0.505642 |
| 12:0 | 3.818556 | 0.01054 |
| 17:0 | 1.609944 | 0.187474 |
| 24:0 | 2.305008 | 0.077263 |
| 20:0 | 6.120062 | 0.00049 |
| 24:1n-9 | 1.6444 | 0.179557 |
| 18:4n-3 | 1.642466 | 0.179993 |
| 22:3n-x | 2.037122 | 0.109108 |
| 16:3n-x | 1.418944 | 0.23764 |
| x-HODE | 0.310226 | 0.81799 |
| x-HODE | 3.085275 | 0.027841 |
| PC(0:0/14:0) | 2.280453 | 0.079758 |
| PC(16:1/0:0) | 2.410448 | 0.067389 |
| PC(18:3/0:0) | 5.084476 | 0.00195 |
| PC(0:0/20:0) | 4.57771 | 0.003834 |
| PC(20:1/0:0) | 1.484229 | 0.219231 |
| PC(20:3/0:0) | 7.779013 | 5.42E-05 |
| PC(22:4/0:0) | 8.767119 | 1.47E-05 |
| PC(0:0/17:0) | 0.60556 | 0.611931 |
| LPC(22:0) | 0.556135 | 0.644461 |
| PC(0:0/17:1) | 0.176778 | 0.912071 |
| LPC(19:1) | 0.278238 | 0.841084 |
| PE(0:0/16:1) | 1.458754 | 0.226251 |
| PE(18:1/0:0) | 3.198934 | 0.023963 |
| LPE(20:5) | 10.59044 | 1.36E-06 |
| PE(22:5/0:0) | 1.990336 | 0.115841 |
| LPI(18:1) | 2.042691 | 0.108332 |
| LPI(18:2) | 3.949316 | 0.008857 |
| LPI(20:4) | 1.931562 | 0.124869 |
| PC(O-20:1/0:0) | 1.633379 | 0.182054 |
| PC(P-20:1/0:0) | 0.993685 | 0.396315 |
| PC(0:0/O-22:0) | 1.336111 | 0.263057 |
| PC(O-22:0/0:0) | 1.369308 | 0.252584 |
| PC(O-24:1/0:0) | 2.069603 | 0.104657 |
| PE(O-16:0/0:0) | 0.169354 | 0.917011 |
| PE(P-20:1/0:0) | 2.305787 | 0.077186 |
| NAE(16:0) | 1.620639 | 0.184982 |
| NAE(18:0) | 0.300667 | 0.824905 |
| NAE(20:4n-6) | 1.983162 | 0.116908 |
| Pregnenolone sulfate | 26.34183 | 6.65E-15 |
| isomer androsterone sulfate | 18.24042 | 9.08E-11 |
| isomer androsterone sulfate | 2.496505 | 0.060252 |
| isomer androsterone sulfate | 15.41868 | 2.93E-09 |
