# Supplementary material for: Data in support of enhancing metabolomics research through data mining
Source: Data Brief. 2015 Feb 27;3:155–64. doi: 10.1016/j.dib.2015.02.008 (PMC4510074; doi:10.1016/j.dib.2015.02.008)
Supplement: Supplementary file 8 — Supplementary Material [file mmc8.doc]

**Supplementary Table 2:** ANOVA analysis. Factors: age and gender. Mean difference is significant at the 0.05 level.

| **Metabolite** | **Age**  **(F value)** | **Age**  **(Pr>F)** | **Gender**  **(F value)** | **Gender**  **(Pr>F)** |
| --- | --- | --- | --- | --- |
| Glutamic Acid | 2.53138249 | 0.057584816 | 9.581947323 | 0.002181555 |
| Alanine | 7.024525301 | 0.000147374 | 2.970636369 | 0.085986499 |
| Serine | 0.115671138 | 0.950870512 | 9.779656985 | 0.001966727 |
| Proline | 1.387888416 | 0.246901376 | 48.93781622 | 2.267E-11 |
| Leucine | 0.778645212 | 0.506813231 | 87.45333553 | 4.32444E-18 |
| Asparagine | 0.882428898 | 0.450712567 | 0.395559754 | 0.529947787 |
| Aspartic Acid | 1.064042811 | 0.364888956 | 3.617357636 | 0.058293372 |
| Lysine | 3.178856178 | 0.024613586 | 10.99281272 | 0.00104601 |
| Histidine | 0.479979377 | 0.696486081 | 0.264789952 | 0.607288847 |
| Phenylalanine | 0.816325671 | 0.485838599 | 1.422904668 | 0.234021751 |
| Arginine | 2.246851801 | 0.083309423 | 1.345403315 | 0.24715559 |
| Cystine | 8.587443731 | 1.86923E-05 | 5.650504404 | 0.018180062 |
| Gly-DL-Phe | 3.026512205 | 0.030090598 | 1.299826766 | 0.25530216 |
| DL-2-Aminoadipic acid | 2.161463285 | 0.093009912 | 55.9141727 | 1.18384E-12 |
| Kynurenine | 3.406090654 | 0.018225518 | 19.80599547 | 1.27615E-05 |
| 1-Methyl-L-histidine | 1.106806786 | 0.346862318 | 22.28639091 | 3.85667E-06 |
| Cer(d18:1/16:0) | 4.582711744 | 0.003810737 | 5.389040048 | 0.021043082 |
| Cer(d18:1/20:0) | 9.339816871 | 6.96777E-06 | 0.253610673 | 0.614973797 |
| Cer(d18:1/21:0) | 4.68891163 | 0.003307447 | 0.393487308 | 0.531026735 |
| Cer(d18:1/24:0) | 4.14744507 | 0.006806974 | 11.49215492 | 0.000808389 |
| Cer(d18:1/24:1)+Cer(d18:2/24:0) | 8.261546249 | 2.87055E-05 | 5.288482581 | 0.022265828 |
| Cer(d18:1/18:0) | 7.580953124 | 7.05082E-05 | 0.678582438 | 0.410836116 |
| Cer(d18:1/25:0) | 12.36673993 | 1.38921E-07 | 9.949395855 | 0.001799573 |
| CMH(d18:1/16:0) | 3.248202895 | 0.022459054 | 0.393558098 | 0.530989815 |
| CMH(d18:1/24:1) | 4.182119642 | 0.006499772 | 0.065929656 | 0.79756274 |
| CMH(d18:1/23:0) | 6.097187265 | 0.000505666 | 0.678963012 | 0.410705109 |
| Hydroxy cholesterol | 10.16582848 | 2.37242E-06 | 2.825380826 | 0.093995058 |
| ChoE(16:1) | 4.858184595 | 0.002638895 | 3.010166438 | 0.083937977 |
| ChoE(17:0) | 3.716570467 | 0.012073924 | 0.082329899 | 0.774394787 |
| ChoE(18:3) | 9.197481734 | 8.39463E-06 | 1.523484634 | 0.218215996 |
| ChoE(22:4) | 0.385184761 | 0.763767 | 3.51832E-05 | 0.995271924 |
| ChoE(17:1) | 4.111514216 | 0.007140576 | 1.578516745 | 0.210110881 |
| PC(16:0/18:0) | 6.193872554 | 0.000444579 | 9.42985768 | 0.002363022 |
| PC(38:5) | 6.18525977 | 0.000449706 | 0.108904353 | 0.741662771 |
| PC(18:2/20:4) | 4.65508488 | 0.003460096 | 1.588973513 | 0.208611672 |
| PC(20:0/20:4) | 0.360672712 | 0.781458727 | 0.000139719 | 0.990578123 |
| PC(40:5) | 1.380751108 | 0.249073975 | 3.864658924 | 0.050385538 |
| PC(18:0/22:5) | 10.97863406 | 8.27131E-07 | 0.486279898 | 0.486219367 |
| PC(40:8) | 4.614332464 | 0.003653373 | 0.05229673 | 0.819295082 |
| PE(16:0/20:4) | 1.208977884 | 0.306943157 | 13.05372546 | 0.000363769 |
| PC(37:2) | 1.264741632 | 0.286939577 | 6.634694811 | 0.01055842 |
| PC(17:0/20:3) | 1.084919761 | 0.355988572 | 5.137835911 | 0.024238099 |
| PC(17:0/20:4) | 0.861490385 | 0.461609509 | 1.498494643 | 0.222019787 |
| PI(18:0/18:2) | 1.304923248 | 0.273266509 | 8.665312236 | 0.003538769 |
| PI(18:0/20:3) | 1.328760239 | 0.265438779 | 0.054006608 | 0.816416854 |
| PI(18:0/22:6) | 0.149403613 | 0.93001076 | 0.574031702 | 0.449352695 |
| DG(36:3) | 2.484740733 | 0.061191809 | 11.3906925 | 0.000851763 |
| DG(36:4) | 1.314714727 | 0.270025937 | 14.12608193 | 0.000211459 |
| 18:1n-9 amide | 0.889253419 | 0.447206634 | 0.989966458 | 0.320682813 |
| 20:0 amide | 1.155144053 | 0.327434775 | 0.0873917 | 0.767757414 |
| 21:1n-x amide | 0.680833148 | 0.564476912 | 0.40827572 | 0.523412833 |
| 22:0 amide | 0.337439339 | 0.798277836 | 0.075451552 | 0.783778497 |
| PC(O-16:0/14:0) | 0.753584895 | 0.521146212 | 3.062921444 | 0.081286303 |
| PC(P-16:0/14:0) | 0.308568713 | 0.819188751 | 3.777233641 | 0.053041877 |
| PC(O-34:0) | 0.130683028 | 0.941785494 | 9.669952785 | 0.002083117 |
| PC(O-18:1/18:2) | 0.367886587 | 0.776244686 | 12.43946592 | 0.000497382 |
| PC(O-16:0/20:3) | 2.907405514 | 0.035196377 | 0.378953557 | 0.53870646 |
| PC(P-36:2) | 0.256528253 | 0.856646045 | 19.07547266 | 1.82109E-05 |
| PC(P-16:0/20:3) | 1.400158237 | 0.243207273 | 0.831205231 | 0.362775322 |
| PC(O-38:4) | 1.261958056 | 0.287909425 | 3.280441077 | 0.071273015 |
| PC(P-18:0/20:4) | 5.691783209 | 0.000867933 | 0.13324998 | 0.715384918 |
| PC(O-18:2/20:4) | 2.682862581 | 0.047249205 | 3.89244265 | 0.04957128 |
| PC(O-18:1/22:4) | 0.290094785 | 0.832540265 | 1.467602385 | 0.226832775 |
| PC(O-40:5) | 0.417367708 | 0.740683086 | 3.507499377 | 0.062222432 |
| PC(O-42:6) | 0.324194916 | 0.807873345 | 11.71988579 | 0.000719038 |
| PC(O-22:0/20:4) | 0.86031952 | 0.46222512 | 7.117454075 | 0.008116959 |
| PE(P-18:0/18:1) | 0.06018602 | 0.980620812 | 0.061307598 | 0.804637974 |
| PE(P-16:0/20:4) | 0.376230652 | 0.770220948 | 2.516167411 | 0.113908991 |
| PE(P-20:0/18:2) | 0.806944892 | 0.490995606 | 0.95910071 | 0.328330977 |
| PE(P-16:0/22:6) | 4.914851358 | 0.002446741 | 1.557055619 | 0.21322819 |
| PC(P-17:0/20:4) | 0.145393634 | 0.932570403 | 0.602166617 | 0.438463313 |
| PE(P-20:0/20:4) | 2.242075978 | 0.083824995 | 2.61487789 | 0.107087654 |
| SM(d18:0/15:0) | 2.738779279 | 0.043913899 | 3.587097164 | 0.05934842 |
| SM(d18:0/16:0) | 11.63867219 | 3.53254E-07 | 17.042119 | 4.93973E-05 |
| SM(d18:0/18:0) | 8.108539839 | 3.51209E-05 | 0.179772584 | 0.671922047 |
| SM(d18:2/16:0) | 11.91111702 | 2.48964E-07 | 22.52986535 | 3.43218E-06 |
| SM(d18:2/20:0) | 4.269117491 | 0.005788485 | 44.5780111 | 1.49041E-10 |
| SM(d16:1/24:1) | 13.25838316 | 4.46334E-08 | 11.16065969 | 0.000959088 |
| SM(42:1) | 7.596611079 | 6.90627E-05 | 4.459324768 | 0.035672808 |
| SM(d18:1/25:0) | 19.06133345 | 3.3875E-11 | 0.865110047 | 0.35318207 |
| SM(43:1) | 6.10131024 | 0.000502897 | 4.788707717 | 0.029543916 |
| SM(43:2) | 5.604422131 | 0.00097516 | 0.701345352 | 0.403107101 |
| TG(43:0) | 0.822913743 | 0.48224251 | 4.888057429 | 0.027920508 |
| TG(53:0) | 0.281768505 | 0.838542383 | 14.87919612 | 0.000144844 |
| TG(54:3) | 1.815527973 | 0.144724884 | 5.747087644 | 0.017227691 |
| TG(56:0) | 0.19559732 | 0.899342718 | 3.80131376 | 0.052295666 |
| TG(56:5) | 6.163220416 | 0.000463099 | 8.964055809 | 0.003020847 |
| TG(56:8) | 1.283575048 | 0.280455234 | 0.642259305 | 0.42363131 |
| TG(58:1) | 1.263232125 | 0.287465148 | 17.08513094 | 4.83593E-05 |
| TG(60:2) | 3.342970291 | 0.019813699 | 12.53812044 | 0.000472958 |
| TG(45:1) | 0.086338032 | 0.967476842 | 7.882274405 | 0.005373426 |
| AC(10:0) | 0.853083342 | 0.46604446 | 0.704421837 | 0.402078871 |
| AC(18:2n-6) | 3.127207134 | 0.026349959 | 23.898969 | 1.7864E-06 |
| AC(10:1n-x) | 1.02787087 | 0.380768911 | 18.5086002 | 2.40224E-05 |
| AC(14:2n-x) | 4.520899955 | 0.004138161 | 9.659216689 | 0.002094878 |
| Taurochenodeoxycholic acid | 6.752243938 | 0.000211553 | 0.012960993 | 0.909448131 |
| Glycocholic acid | 5.011558516 | 0.002150559 | 10.92951584 | 0.001080837 |
| Glycochenodeoxycholic acid | 8.153977228 | 3.30783E-05 | 26.58976615 | 5.01407E-07 |
| 16:1n-7 | 3.592011208 | 0.01424479 | 10.59150262 | 0.001287861 |
| 16:1n-x | 1.67777896 | 0.172200479 | 0.556176759 | 0.456483718 |
| 18:0 | 2.798521542 | 0.040606101 | 0.628864658 | 0.428500715 |
| 20:1n-9 | 1.338256872 | 0.262377804 | 1.621675495 | 0.204004444 |
| 20:4n-6 | 0.932710631 | 0.425405249 | 0.076119604 | 0.782847413 |
| 20:4n-3 | 0.78189555 | 0.504976652 | 1.393575533 | 0.238889403 |
| 12:0 | 3.825654747 | 0.010444852 | 1.481491134 | 0.224653528 |
| 17:0 | 1.607637121 | 0.188025926 | 0.628860087 | 0.428502391 |
| 24:0 | 2.355217395 | 0.072406642 | 6.641709003 | 0.010517991 |
| 20:0 | 6.122550843 | 0.00048887 | 1.10533393 | 0.294082227 |
| 24:1n-9 | 1.66708139 | 0.174529822 | 4.572355718 | 0.033431401 |
| 18:4n-3 | 1.652984348 | 0.17764496 | 2.65862095 | 0.104210136 |
| 22:3n-x | 2.048351449 | 0.107559358 | 2.427689965 | 0.12043424 |
| x-HODE | 3.207802684 | 0.023690446 | 11.2858856 | 0.000899057 |
| PC(16:1/0:0) | 2.525476168 | 0.058029798 | 13.35967563 | 0.000311463 |
| PC(18:3/0:0) | 5.075493669 | 0.001974713 | 0.542443231 | 0.462091084 |
| PC(0:0/20:0) | 4.5606109 | 0.003924726 | 0.032554183 | 0.856958391 |
| PC(20:1/0:0) | 1.488390934 | 0.21811372 | 1.726257737 | 0.190056573 |
| PC(22:4/0:0) | 8.989360844 | 1.10268E-05 | 7.56551994 | 0.006370789 |
| PC(0:0/17:0) | 0.604048378 | 0.612912673 | 0.353285512 | 0.552779996 |
| LPC(22:0) | 0.57528382 | 0.631737179 | 9.917804069 | 0.001829547 |
| PC(0:0/17:1) | 0.182767728 | 0.908050936 | 9.774963093 | 0.001971569 |
| LPC(19:1) | 0.293595738 | 0.830013253 | 15.29585958 | 0.000117588 |
| PE(0:0/16:1) | 1.483456209 | 0.219451018 | 5.385866198 | 0.021080586 |
| PE(18:1/0:0) | 3.280201088 | 0.021528904 | 7.579706 | 0.006322288 |
| LPE(20:5) | 10.56997175 | 1.40378E-06 | 0.499456931 | 0.480376611 |
| PE(22:5/0:0) | 2.128314536 | 0.097063799 | 18.95502177 | 1.93133E-05 |
| LPI(18:1) | 2.042632246 | 0.108350682 | 0.99261277 | 0.320038118 |
| LPI(18:2) | 3.936885132 | 0.009008811 | 0.184752859 | 0.667678902 |
| LPI(20:4) | 1.952879258 | 0.121529654 | 3.858454906 | 0.050569294 |
| PC(O-20:1/0:0) | 1.636966663 | 0.18124832 | 1.568816203 | 0.211513148 |
| PC(P-20:1/0:0) | 0.992228863 | 0.396995826 | 0.620478764 | 0.431592502 |
| PC(O-22:0/0:0) | 1.366623099 | 0.253426599 | 0.492182993 | 0.483587472 |
| PC(O-24:1/0:0) | 2.068366709 | 0.104833712 | 0.845313154 | 0.358740401 |
| PE(O-16:0/0:0) | 0.169375109 | 0.916996323 | 1.032622971 | 0.31049539 |
| PE(P-20:1/0:0) | 2.310922863 | 0.076683975 | 1.576945933 | 0.210337198 |
| NAE(16:0) | 1.683652652 | 0.170934068 | 11.07036762 | 0.001004892 |
| NAE(18:0) | 0.305871915 | 0.821140038 | 5.48398842 | 0.019952675 |
| NAE(20:4n-6) | 2.104895265 | 0.100030472 | 16.89837178 | 5.3033E-05 |
| Pregnenolone sulfate | 29.74333978 | 1.50669E-16 | 34.44453189 | 1.34208E-08 |
| isomer androsterone sulfate | 25.69228466 | 1.41481E-14 | 106.8107594 | 3.55391E-21 |
| isomer androsterone sulfate | 2.775404448 | 0.041855908 | 29.93441774 | 1.05675E-07 |
| isomer androsterone sulfate | 23.38732088 | 2.01184E-13 | 134.8556956 | 2.33147E-25 |
